# Hierarchical Environmental Exposure Transforms Zeolitic Imidazolate Framework‑8 and Increases Toxicity in *Daphnia magna*

**DOI:** 10.1021/acsnano.6c01107

**Published:** 2026-05-26

**Authors:** Swaroop Chakraborty, Iuliia Mikulska, Rhiannon Boseley, Sang Pham, Prathmesh Bhadane, Pankti Dhumal, Santanu Majumder, Jajati Mandal, Tina Geraki, Superb K. Misra, Christian Pfrang, Iseult Lynch

**Affiliations:** † School of Geography, Earth & Environmental Sciences, 1724 University of Birmingham, Edgbaston, Birmingham B15 2TT, U.K.; ‡ Diamond Light Source, Harwell Science and Innovation Campus, Didcot OX11 0DE, U.K.; § Facility of Electron Microscopy, University of Birmingham, Edgbaston, Birmingham B15 2TT, U.K.; ∥ 242275 Materials Engineering, Indian Institute of Technology, Gandhinagar 382355, India; ⊥ School of Life and Environmental Sciences, 6657 Bournemouth University (Talbot Campus), Fern Barrow, Poole BH125BB, U.K.; # School of Science, Engineering and Environment, 7046 University of Salford, Salford M5 4WT, U.K.

**Keywords:** metal−organic frameworks, ZIF-8, Daphnia magna, safe-and-sustainable-by-design, environmental transformation, ecotoxicology

## Abstract

Metal–organic frameworks (MOFs) are increasingly deployed in environmental technologies, yet their fate and hazard under realistic multistep exposure scenarios remain poorly constrained. Here, we track hierarchical transformations of nanoscale ZIF-8 (Zeolitic Imidazolate Framework-8) across an exposure cascade spanning atmospheric aging (air and reactive gases O_3_/NO_2_), aqueous aging in environmentally and biologically relevant media, and ingestion by the freshwater crustacean *Daphnia magna*. Synchrotron Zn K-edge X-ray absorption spectroscopy (XAS), micro-X-ray fluorescence (μ-XRF), X-ray photoelectron spectroscopy (XPS), and electron microscopy show that gas-phase exposure produces only minor surface perturbations, whereas aqueous contact drives pronounced medium-dependent restructuring, including nitrogen depletion and oxygen enrichment at the surface and time-resolved dissolved Zn release with chemistry-imposed plateaus. In vivo, Zn speciation diverges from the pristine Zn–N fingerprint; an unexposed endogenous Zn baseline and linear combination fitting (LCF) indicate a mixture of endogenous Zn with transformed Zn pools dominated by O/P/S-type coordination environments. Acute ecotoxicity assay demonstrates strong concentration dependence (48 h immobilization EC_50_ ≈0.5 μg mL^–1^), and chronic exposure at 0.10 μg mL^–1^ reduces cumulative brood production with increased adult mortality over 24 days. Mechanistically, fractionated toxicity assays show that washed aged particles/precipitates and whole aged suspensions are more potent than particle-free filtrates, indicating that particle-associated transformed Zn pools contribute substantially beyond dissolved Zn alone. Together, these results show that ZIF-8 risk emerges from its sequential transformation trajectory rather than its pristine state, motivating tiered aging protocols coupled to in vivo speciation and fractionated hazard testing for MOF safety assessment.

## Graphical abstract

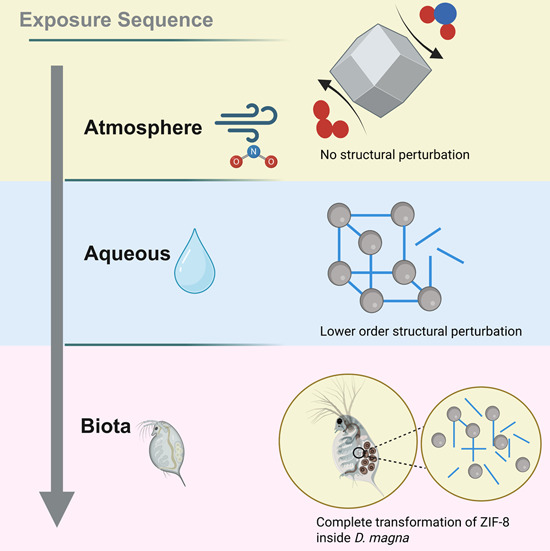

## Introduction

Metal–organic frameworks (MOFs) are a modular class of porous crystalline materials whose internal surface chemistry can be engineered for selective adsorption, separations, and catalysis. Their rapid expansion into water-facing applications (e.g., contaminant adsorption, catalytic degradation, and wastewater treatment) is driven by high sorption capacity and tunable functionality.^1^ Yet translation from laboratory performance to real environmental benefit depends on a more basic question: what do MOFs become once they leave controlled reactors, and how do those transformation products shape bioavailability and hazard?

A broad literature now shows that MOF “stability” is strongly context-dependent, governed by metal–ligand bond character, node connectivity/defects, and the chemistry of the surrounding matrix (pH, ionic strength, carbonate alkalinity, phosphate, and competing ligands/biomolecules).^2,3^ Even frameworks regarded as robust can undergo ligand exchange, linker loss, partial amorphization, and secondary-phase formation without immediate collapse in bulk crystallinity, particularly in buffered and ligand-rich environments. A key practical implication is that the identity of the medium can be decisive: systematic “MOFs vs buffers” studies demonstrate that common aqueous chemistries—especially phosphate-containing systems—can rapidly redirect apparent stability and speciation outcomes.^4^

To move beyond single-material assumptions, it is essential to test whether transformation–hazard relationships are general across MOF classes and where they diverge with metal center and linker chemistry. UiO-66 (Zr^4+^–carboxylate) is widely treated as a benchmark for hydrolytic robustness owing to strong Zr–O bonding and high node connectivity.^5^ In our recent work, abiotically stable nanoscale UiO-66 nonetheless underwent profound in vivo respeciation after ingestion by *Daphnia magna* (*D. magna*), forming Zr-hydroxide-like products and producing chronic reproductive toxicity—highlighting that abiotic robustness does not guarantee biotic inertness.^6^ ZIF-8 (Zn^2+^–imidazolate) represents a chemically orthogonal archetype (Zn–N coordination; distinct aqueous Zn speciation and precipitation pathways), for which transformation end points are expected to differ fundamentally from Zr-carboxylate systems. Demonstrating how a Zn-imidazolate framework behaves within the same hierarchical logic therefore constitutes more than an incremental extension: it provides a chemistry-dependent test of the framework and exposes divergent “commit points” that are directly actionable for safe-and-sustainable design.

ZIF-8 (Zeolitic Imidazolate Framework-8; Zn­(2-methylimidazolate)_2_) is extensively studied and proposed for environmental separations and adsorption owing to its high porosity and perceived chemical stability. However, independent studies have shown that ZIF-8 is not indefinitely stable in water under ambient conditions, with dissolution releasing Zn and imidazolate species in a manner dependent on solid-to-liquid ratio and exposure conditions.^7^ In phosphate-buffered saline, ZIF-8 undergoes pronounced structural and chemical change, consistent with phosphate-driven transformation pathways and zinc phosphate-rich products.^8^ Moreover, ZIF-8 degrades in cell media, serum and some common laboratory buffers, emphasizing that biologically relevant matrices and biomolecules can substantially alter speciation trajectories relative to simple waters.^9^ Collectively, existing work establishes that ZIF-8 can transform across aqueous and biological matrices, but it remains unclear how sequential preaging across compartments shapes (i) the identity of transformed Zn pools, (ii) their localization/retention in organisms, and (iii) the resulting organism-level effects.

Ecotoxicological evidence further motivates a transformation-aware approach. ZIF-8 and related ZIF nanoparticles have been reported to elicit adverse responses in primary producers and aquatic organisms, and mixture contexts can amplify outcomes (e.g., coexposure with surfactants).^10–12^ Plant and algal studies also show that ZIF-8/ZIF-67 can drive measurable biological effects at low mg L^–1^, with recovery dynamics suggesting nontrivial interactions between exposure form and organismal response.^13^ More broadly, recent syntheses of nanoscale MOF safety emphasize that hazard cannot be inferred from pristine structure alone because dissolution, aging, corona formation, and secondary-phase generation can shift bioavailability and mode of action. This is especially relevant for water-treatment MOFs, where environmental release routes plausibly include discharge into carbonate-/phosphate-containing freshwaters, co-occurrence with natural organic matter and other contaminants, and subsequent entry into food webs.

Quantitative predicted environmental concentrations (PECs) for MOFs such as ZIF-8 are not yet established, because production volumes, product formats, and use-phase release rates remain poorly constrained. In water-facing deployments, however, several plausible release routes exist: (i) handling and transfer losses of powders during manufacture/processing; (ii) attrition and detachment of fines from sorbent beds, granules, or MOF–polymer composites during operation and regeneration/backwash; (iii) release from MOF-modified membranes/coatings during shear and cleaning; and (iv) end-of-life disposal and leaching of spent materials.^1,14^

In the broader engineered-nanomaterial literature, probabilistic material-flow and fate models typically predict regional surface water concentrations in the ng L^–1^–low μg L^–1^ range while emphasizing that localized point sources (e.g., wastewater treatment plant discharges, industrial outfalls, episodic releases) can generate transient hotspots that dominate exposure and risk.^15–17^

We therefore frame our exposure window as a scenario-based bounding assessment: environmentally realistic media chemistries (Table S1a) combined with ecotoxicology spanning low-dose chronic effects through higher concentrations used to resolve transformation mechanisms and establish concentration–response behavior.

In this study, we use *D. magna* as the biotic tier (in vivo processing in *D. magna*) because it uniquely aligns the hierarchical exposure-cascade logic with regulatory and ecological relevance. *D. magna* underpins standardized freshwater hazard testing (OECD TG 202 acute immobilization; OECD TG 211 chronic reproduction), enabling transformation trajectories to be anchored to decision-grade end points.^18,19^ Mechanistically, *D. magna* is a filter-feeding grazer with high probability of ingesting suspended particles; gut processing provides a realistic internal environment in which partially aged materials can undergo further in vivo respeciation and retention, a recognized driver of nanomaterial bioavailability and effects.^20^ Finally, as a key node in freshwater food webs, *D. magna* represents an ecologically meaningful entry point for particle-associated contaminants, and dietary transfer from *D. magna* to fish has been demonstrated for nanoparticles supporting the relevance of this tier for pathway-level risk thinking.^21^

Here, we integrate a hierarchical exposure cascade (air → aqueous media → *D. magna*) with synchrotron-based Zn K-edge X-ray absorption spectroscopy (XAS), μ-X-ray fluorescence (XRF) mapping, and acute/chronic ecotoxicology to determine how ZIF-8 transforms across compartments and how the resulting Zn pools control realized hazard. By linking dissolved Zn release, particle-associated transformed pools, and in vivo localization/speciation to organism-level outcomes, this work provides a transferable framework for evaluating transformation-dependent bioavailability and toxicity of MOFs intended for water-facing applications.

## Results and Discussion

We designed the study as an exposure cascade to emulate a plausible trajectory for water-facing MOFs: atmospheric conditioning during handling/storage (primary tier), followed by aqueous aging across a chemistry gradient that controls Zn speciation (secondary tier), and finally biotic processing in the filter-feeding freshwater crustacean *D. magna* (Tertiary Tier). Accordingly, we first establish the pristine reference state, then test whether air/reactive gases meaningfully perturb ZIF-8, quantify medium-dependent restructuring and dissolved Zn release in representative waters/biological fluids, and finally determine whether transformed Zn pools become bioaccessible in vivo and drive acute and chronic outcomes.

### Physicochemical Characterization of Nanoscale ZIF-8 MOFs

Pristine ZIF-8 nanoparticles were synthesized using an established room-temperature precipitation route. Electron microscopy confirms nanoscale particles with a mean diameter of 47 ± 4 nm (*n* = 50). Powder X-ray Diffraction (PXRD) matches the sodalite ZIF-8 phase with no detectable byproducts, and Fourier Transformed Infrared Spectroscopy (FTIR) confirms coordination of 2-methylimidazole.^22^ These baseline data sets provide the reference state for tracking subsequent coordination and surface chemistry changes during hierarchical aging; full synthesis details are provided in Supporting Information Section S1, and baseline characterization is provided in Section S2 and Figure S1.

### Primary Transformation in Atmospheric Conditions

Zn K-edge X-ray absorption near edge structure (XANES) and Fourier transformation (FT) of extended X-ray absorption fine-structure (EXAFS) confirm that ZIF-8 retains its characteristic local structure after activation, storage in air and short-term exposure to oxidizing/reactive gases (Figure 1, Figures S2–S4). Comparison of Zn K-edge XANES spectra measured on pristine and activated ZIF-8 (Figures 1a,b and S2) shows nearly identical spectral profiles, with only slight intensity differences near the white line and the feature at 9667.7 eV. In EXAFS magnitude |χ­(*R*)|, both materials exhibit a dominant first-shell peak at ∼1.5 Å (phase-uncorrected) and similar higher-shell features extending to ∼4.5 Å. Only a small decrease in the amplitude of the first shell is observed upon activation, consistent with slight increases in static disorder and removal of guest molecules from the pores rather than any change in the Zn (II) oxidation state or Zn–N coordination environment. Transmission Electron Microscopy (TEM) (Figures S1 and 4a) likewise shows that activation leaves particle morphology and crystallinity essentially unchanged, with faceted nanocrystals and sharp diffraction rings.

**Figure 1:**
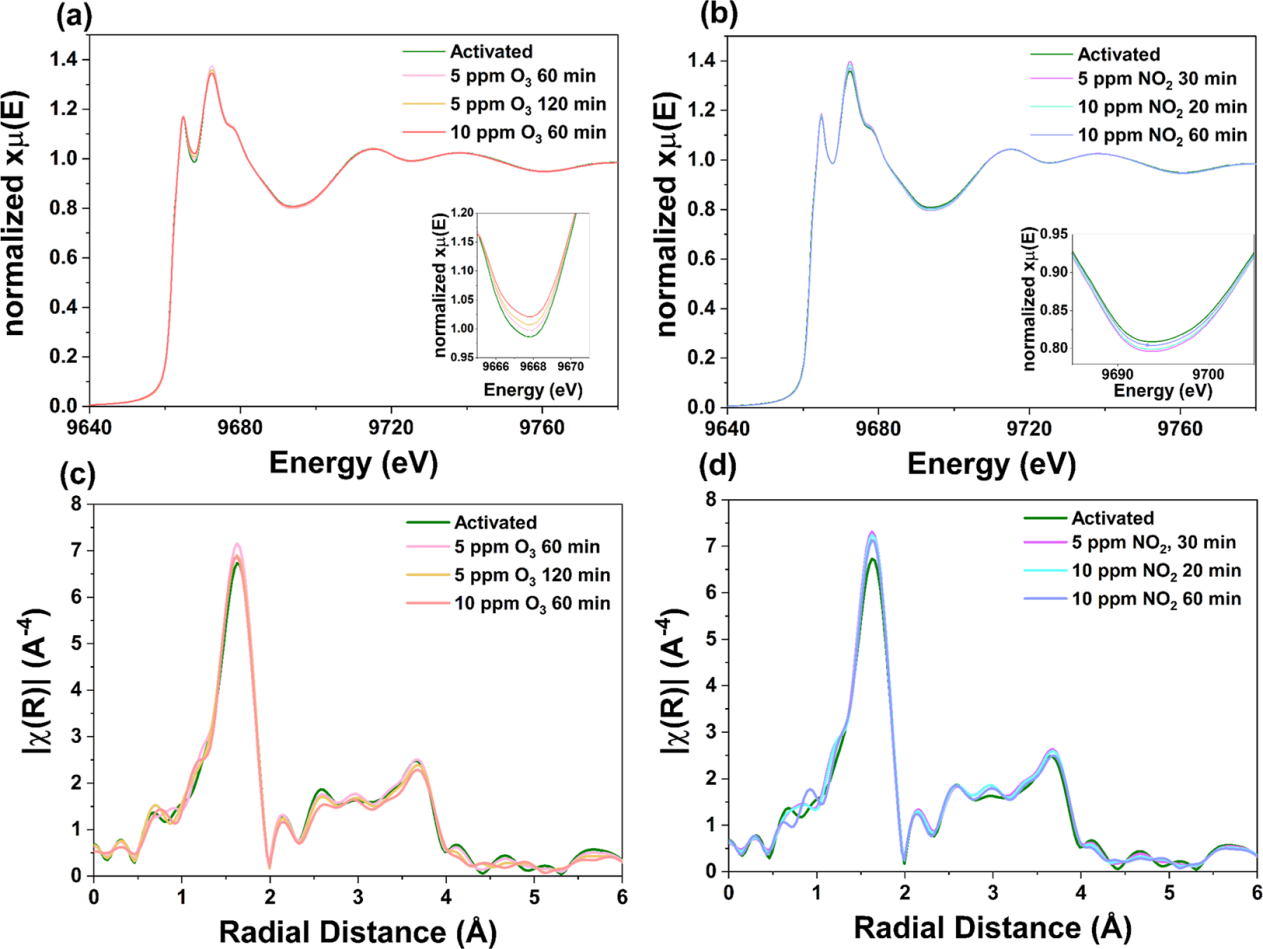
Primary gas-phase transformations of ZIF-8 probed by Zn K-edge XAS. (a,b) Normalized Zn K-edge XANES spectra of activated ZIF-8 and samples exposed to ozone (O_3_) (a) or nitrogen dioxide (NO_2_) (b) at the indicated mixing ratios and exposure times. Insets highlight the evolution of the spectral feature, showing a progressive increase of the feature at ∼9667.7 eV with increasing O_3_ dose and a nonmonotonic change in intensity of spectral feature at 9694 eV for NO_2_, with the strongest enhancement at 5 ppm for 30 min. (c,d) Corresponding Fourier-transformed EXAFS spectra (magnitude of χ­(*R*)) for O_3_- (c) and NO_2_-exposed (d) samples. Gas exposure induces subtle but systematic perturbations of the local Zn environment, including an outward shift of the second coordination shell and damping of higher-shell amplitudes, while the overall Zn (II) edge position and first-shell distance remain essentially unchanged, indicating limited modification of the ZIF-8 framework under these oxidative atmospheres.

When the activated material is exposed to ozone (O_3_) at 5 or 10 ppm for up to 120 min, the XANES spectra remain highly similar to the activated reference. The inset reveals a modest, monotonic increase in the spectral feature at 9667.7 with increasing O_3_ dose, while the edge energy and overall line shape remain fixed. The FT-EXAFS data (Figure 1c) show that all O_3_-treated samples preserve a strong first-shell peak at ∼1.5 Å; subtle changes are confined to a slight consistent intensity diminishment and outward shift of the FT peaks at 2.6 Å and 3.6 Å (∼3 Å) with prolonged gas exposure. Nitrogen dioxide (NO_2_) exposure yields a slightly different pattern (Figure 1b,d). At 5 ppm of NO_2_ for 30 min, the white-line intensity is marginally higher than that of the activated sample, but further exposures at 10 ppm for 20- and 60 min lead to a small consistent decrease in the white-line height (inset, Figure 1b), while the edge position remains constant. In |χ­(R)| (Figure 1d), the first-shell Zn–N peak again remains centered at ∼1.5 Å for all conditions, while the medium-range features at 3 Å exhibit higher intensity in samples exposed to NO_2_ compared to the activated sample, suggesting subtle local structure changes around Zn atoms associated with NO_2_ molecules within the ZIF-8 pores.

The robustness of the Zn local environment under these gas-phase conditions is highlighted by comparison with Zn reference compounds (Figure S4). XANES and FT- EXAFS spectra for ZnO, ZnSO_4_, Zn­(NO_3_)_2_, and ZnF_2_ differ strongly from all ZIF-8-based samples in both XANES profiles and FT magnitudes. These data show that gas-phase aging produces, at most, very mild primary transformations in ZIF-8. The invariance of the edge position and the persistence of a sharp first-shell peak at ∼1.5 Å across pristine, activated, air-exposed and gas-exposed samples demonstrate that Zn remains tetrahedrally coordinated, with Zn–N distances characteristic of the sodalite-type framework.^22–24^ The small changes in Zn K-edge XANES profiles and subtle modulation of the second-shell region in FT magnitudes point to low-level rearrangements in the local ligand field rather than wholesale changes in the oxidation state or coordination number. Crucially, however, none of these changes are large enough to indicate framework collapse or conversion to a ZnO-like phase, which would produce a dramatically different EXAFS pattern. The spectra of gas-aged ZIF-8 remain clearly distinct from those of ZnO and Zn salts (Figure S4), and TEM/Selected area electron diffraction (SAED) shows that crystals remain well-defined and highly crystalline in air-exposed samples (Figure 4f). This behavior is consistent with broader assessments of ZIF-8 stability, which emphasize its tolerance to dry gases and identify water, acidic conditions, or strongly coordinating anions as the main drivers of degradation.^24,25^ We note that sunlight/UV irradiation may further influence ZIF-8 aging and could accelerate hydrolysis or ligand–metal bond disruption under coupled humidity conditions. Published studies report photoaccelerated hydrolysis of ZIF-8 in water under UVA/UVB/near-UV visible irradiation and UV-induced partial disconnection in ZIF-8 crystals/films; photochemical aging under controlled solar-simulated conditions will therefore be explored in future work.^25,26^

### Secondary Transformation in Aqueous Conditions

The air-exposed ZIF-8 was then transferred to a range of aqueous environmental and biological media as discussed in Box [Boxed-text box1] and detailed in Table S1. These media span a gradient of pH, ionic strength, and organic content, enabling a systematic evaluation of ZIF-8’s stability under environmentally and biologically relevant conditions. Notably, ZIF-8 is a zinc-imidazolate framework known to be generally stable in dry air but susceptible to slow hydrolysis in water. Prolonged contact with water can break Zn–N imidazolate bonds, releasing Zn^2+^ ions and free 2-methylimidazole ligands into solution.^7^ Even at room temperature, measurable dissolution of ZIF-8 has been reported, with the extent depending on factors like pH, ionic strength, and exposure duration. By subjecting ZIF-8 to the diverse chemistries, we aimed to capture a range of possible secondary transformation pathways—from minimal changes in simple water to accelerated framework degradation in harsher matrices. For instance, high-salinity conditions (as in ASW) can favor deprotonation of the imidazole linker and Zn–OH formation, while organics and biomolecules in WW or CCM might chelate Zn or otherwise destabilize the framework. In contrast, the BHW scenario offers insight into how ZIF-8 behaves in natural freshwaters that contain carbonate and calcium (which could precipitate zinc or compete for coordination sites).

1 Operational Definition of Hierarchical Transformation Used in This Study

Hierarchical transformation (this study) refers to sequence-defined aging across compartments, where a material undergoes transformations under distinct boundary conditions in a defined order. We operationalize three tiers:

Primary transformation (atmosphere): aging under air exposure (and, separately, an accelerated oxidative boundary-condition stress test with O_3_/NO_2_). This tier tests whether dry atmospheric conditions can precondition the framework prior to water contact.

Secondary transformation (aqueous media): aging in a set of aqueous environments spanning low-complexity electrolyte, marine ionic strength, natural hard freshwater (BHW), wastewater-like organic/nutrient complexity, and protein-rich biological medium. This tier captures hydrolysis, ligand exchange, complexation, and secondary-phase formation that control Zn speciation once released from the lattice.

Tertiary transformation (biotic): exposure to and processing by *D. magna*, representing ingestion-driven transformation and retention (gut-mediated respeciation) that can diverge from abiotic aging outcomes.

The tiers are termed “hierarchical” because the order of exposure can precondition subsequent transformations (e.g., modest surface changes formed in air may influence hydrolysis/complexation in water, while aqueous aging can determine the form that enters biota).

Upon transfer from air to aqueous environments, ZIF-8 undergoes markedly stronger structural reorganization than under gas-phase aging (Figure 2). After 7 days in 1 mM NaNO_3_, Artificial Seawater (ASW), Borehole water (BHW), simulated wastewater (WW), or complete cell culture media (CCM), the Zn K-edge XANES spectra deviate substantially from the pristine framework (Figure 2a). The corresponding FT-EXAFS spectra (Figure 2b) reinforce this picture. All aqueous-aged samples retain a dominant first-shell peak near ∼1.5 Å, but the peak shape and the distribution of intensity at higher radial distances differ strongly from the pristine material. There are significant intensity changes of spectral features in the second-shell region (2.5–4 Å), consistent with a partial cleavage of Zn–N-imidazolate bonds, possibly due to formation of Zn–O­(H) species and increased structural disorder. The extent of this restructuring is medium-dependent: samples aged in ASW, BHW, and WW display broadly similar distortions, whereas those aged in CCM show the largest departures from the pristine pattern, suggesting that both simple electrolytes and complex, ligand-rich solutions promote extensive hydrolysis and recoordination of Zn. This medium dependence is consistent with the differing water chemistries used here, which span low-to high-ionic-strength matrices and include varying phosphate and organic matter contents (Table S1b). These observations align with previous reports that ZIF-8 is only metastable in water and buffered solutions, gradually releasing Zn^2+^ and linker, and that anions and organic ligands can accelerate dissolution and drive formation of new Zn–O-rich phases.^2^ Thus, secondary transformation in aqueous media represents a major inflection point in the hierarchical pathway: a nominally “water-stable” framework evolves into a family of medium-specific, disordered Zn complexes and secondary solids long before it encounters biological systems. These observations agree well with independent reports that ZIF-8 is not indefinitely water-stable. In neutral water and buffered solutions, ZIF-8 crystallites hydrolyze to release Zn^2+^ and imidazolate, followed by condensation into new Zn-containing phases upon solvent removal or under phosphate-rich conditions.^27,28^ Phosphate and other anions can strongly accelerate degradation and drive formation of zinc phosphate-like solids, even at near-neutral pH.^29,30^

**Figure 2:**
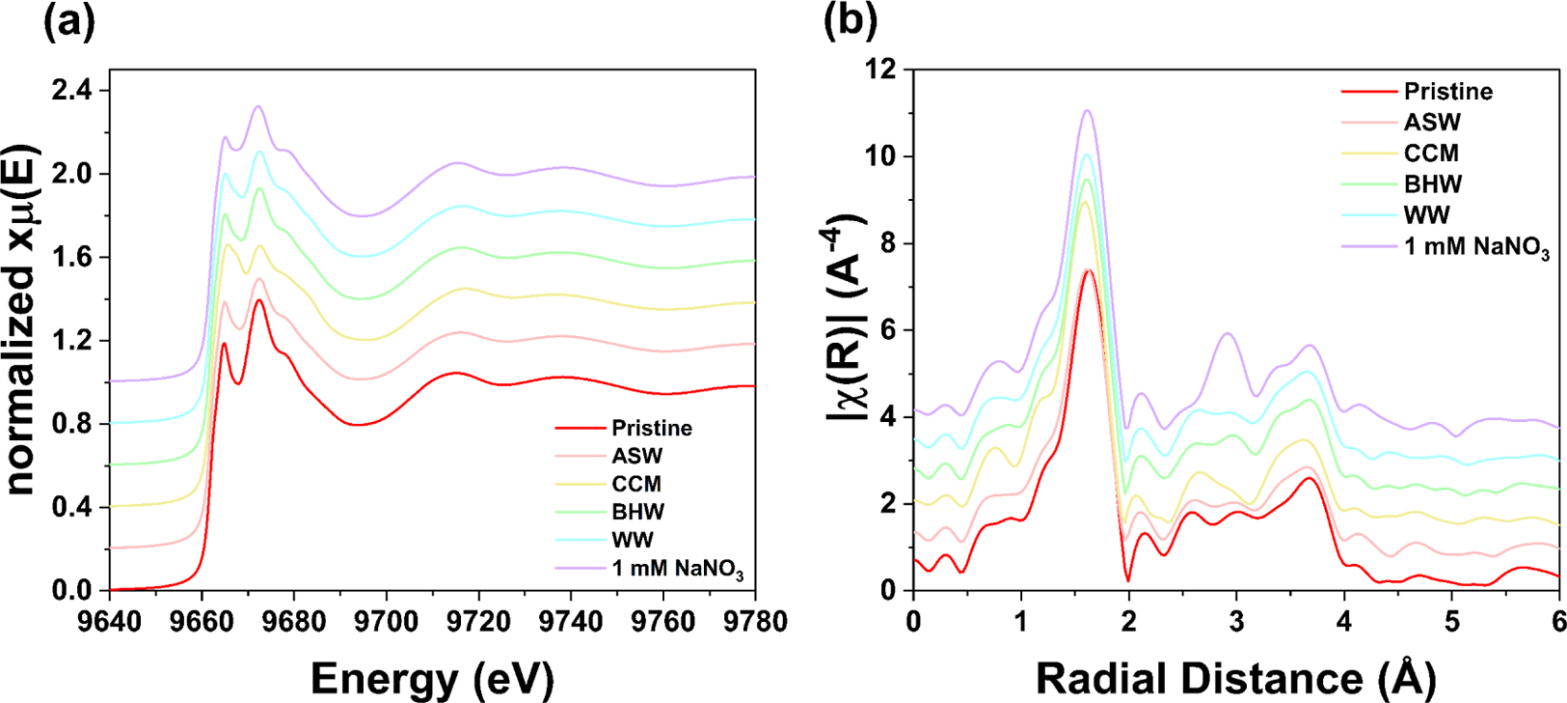
Secondary aqueous transformations of ZIF-8 revealed by Zn K-edge XAS. (a) Normalized Zn K-edge XANES spectra of pristine ZIF-8 and samples aged for 7 days in different aqueous media: artificial seawater (ASW), Dulbecco’s modified Eagle’s medium (DMEM) containing 10% fetal bovine serum also known as complete culture medium (CCM), borehole water (BHW), simulated wastewater (WW), and 1 mM NaNO_3_. All liquid-aged samples show marked changes in the white-line region relative to the pristine material, consistent with partial loss of Zn-imidazolate coordination and increasing Zn–O/N environments. (b) Corresponding Fourier-transformed EXAFS (|χ­(*R*)|) spectra. Aqueous aging leads to a reorganization of the second shell, with the most pronounced deviations in complex, nutrient-, and phosphate-rich media (CCM, WW), indicating substantial hydrolysis and medium-dependent restructuring of the ZIF-8. Zn K-edge XANES and FT EXAFS spectra are shifted vertically for clarity.

The contrast between ZIF-8 and our previously reported UiO-66 illustrates how metal-center identity and linker class can govern the hierarchy of transformations.^31^ ZIF-8 comprises Zn^2+^ nodes coordinated by imidazolate linkers (Zn–N). In aqueous environments containing strong O-donor ligands (e.g., phosphate and bicarbonate/carbonate) or complexing biomolecules, Zn can be displaced from Zn–N coordination and rebound into Zn–O and Zn–O–P environments, accelerating restructuring and, in some cases, dissolution. The chemistry of the tested media is consistent with this interpretation: ASW is dominated by Cl^–^ and SO_4_^2–^ at high ionic strength, BHW contains HCO_3_^–^ together with Cl^–^, SO_4_^2–^, NO_3_^–^, and trace phosphate, 1 mM NaNO_3_ provides a simple nitrate-only baseline, while WW and CCM both contain phosphate together with substantially greater organic complexity. These differences in anion composition, ionic strength, and organic matter are likely to contribute to the distinct restructuring behavior observed across the five media and are summarized in Table S1b.^9,30^ UiO-66, by contrast, is built from Zr^4+^–carboxylate bonds (Zr–O) within highly connected Zr_6_ nodes, which contributes to high kinetic stability in many conditions; nevertheless, systematic studies on UiO-66 MOFs interactions with buffers show that phosphate buffers can drive rapid terephthalate release and amorphization, demonstrating that Zr-MOFs can also transform under environmentally and biologically relevant anion chemistries.^32^

These differences motivate using metal-center/linker chemistry as a screening axis for SSbD: Zn-imidazolates may preferentially form Zn–O/P/C secondary phases in phosphate-/carbonate-bearing waters, while Zr-carboxylates may retain crystallinity longer yet still undergo defect generation and linker loss in specific chemistries.^6^

FTIR spectra provide a complementary view of how aqueous aging reshapes the chemical environment of ZIF-8 (Figure 3a). Pristine ZIF-8 exhibits the expected vibrational features of the 2 methylimidazolate (mIm) linker: C–H stretching modes in the 3100–2900 cm^–1^ region (Figure S1d), strong C=N and C–N stretching bands between ∼1600 and 1350 cm^–1^, and ring-breathing and Zn–N-related modes below 1200 cm^–1^, in good agreement with the literature.^33^ After 7 d in the different media, all spectra show broadening and intensity redistribution across the 1800–1000 cm^–1^ window. The main linker bands remain visible, indicating that a fraction of the Zn-imidazolate framework persists, but their relative intensities decrease and shoulders develop on both the low- and high-frequency sides. In 1 mM NaNO_3_, ASW, and BHW, new or enhanced bands appear around ∼1400–1500 cm^–1^ and ∼1050–1100 cm^–1^, which can be attributed to coordinated and adsorbed carbonate/bicarbonate species and Zn–O–H vibrations, signaling partial hydrolysis of Zn–N bonds and binding of inorganic anions at the surface. Changes are most pronounced for WW and especially CCM, which contain organic matter, phosphate, and multivalent ions. These samples show broader features in the 1650–1550 cm^–1^ region—consistent with amide and carboxylate vibrations—as well as increased intensity in the 1200–1000 cm^–1^ range associated with C–O stretching and phosphate groups. Key ZIF-8 fingerprint bands annotated in Figure 3a (∼1580 cm^–1^, ∼1145 cm^–1^, and ∼995 cm^–1^) remain identifiable across all media, enabling direct visual comparison of band broadening and intensity redistribution after aging. The progressive evolution from a sharp, linker-dominated spectrum (pristine) to broader, composite spectra in complex media is therefore consistent with partial linker loss, formation of Zn–O­(H) environments, and adsorption of organic/inorganic corona components onto the ZIF-8 surface. Together with the XAS data, this supports a dissolution-restructuring pathway in which the MOF is gradually decorated and partially replaced by medium-derived species.

**Figure 3:**
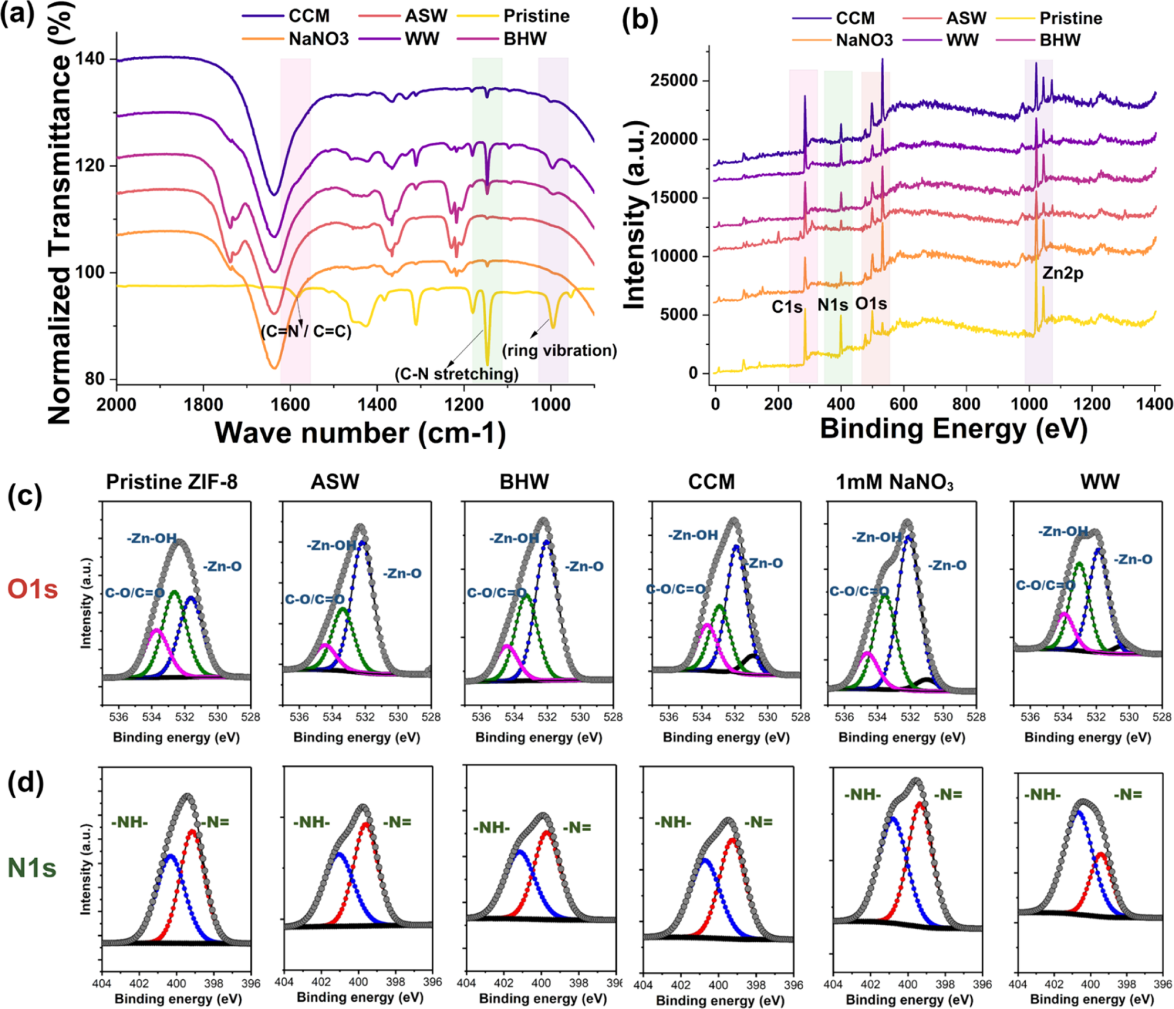
FTIR and XPS signatures of aqueous aging of ZIF-8. (a) Stacked FTIR spectra of pristine ZIF-8 and samples aged for 7 d in 1 mM NaNO_3_, artificial seawater (ASW), borehole water (BHW), simulated wastewater (WW), and complete culture medium (CCM; DMEM + 10% fetal bovine serum). The pristine spectrum shows characteristic 2-methylimidazolate (mIm) linker bands (e.g., ∼1580 cm^–1^, ∼1145 cm^–1^, and ∼995 cm^–1^), while aged samples display medium-dependent band broadening and intensity redistribution consistent with surface conditioning by oxygenated/inorganic and organic components. (b) Stacked XPS survey spectra for the same series highlighting the C 1s, N 1s, O 1s, and Zn 2p regions. Bottom panels: high-resolution O 1s (top row) and N 1s (bottom row) spectra with peak deconvolution. O 1s components are assigned to Zn–O/Zn–OH­(H_2_O) and C–O/C=O environments, and N 1s components to imidazolate (−*N*=) and –NH-/amide-type nitrogen, indicating progressive nitrogen depletion and oxygen enrichment consistent with aqueous aging and (in CCM) biomolecular adsorption/corona formation.

To elucidate the oxidation state and chemical composition of the prepared ZIF-8 nanoparticle, the X-ray photoelectron spectroscopy (XPS) analysis was performed, and the survey spectrum provides clear evidence for the presence of Zn, O, N, and C, elements in the synthesized pristine and medium-transformed ZIF-8 (Figure 3b). XPS analysis of the O 1s and N 1s regions (Figure 3c,d) provides detailed insights into the chemical environments of ZIF-8 following exposure to various media. The O 1s spectra across all samples revealed three principal deconvoluted peaks: the first at approximately 531.5 eV, assigned to lattice oxygen and Zn–O, which shifted to lower energy (530.8 eV) in CCM, indicating increased Zn–O coordination.^34^ The second peak, centered around 532.8 eV, corresponds to Zn–OH bonds and remains a prominent feature in most samples, while the third peak at 533.6 eV becomes more intense in WW and CCM due to enhanced adsorption of organics and carbonate species. These peak shifts and intensity variations reflect the impact of ionic strength, organic content, and multivalent cations on ZIF-8 surface chemistry. Many studies on ZnO-/ZIF-derived systems show a distinct O 1s feature at ∼531 eV arising from chemisorbed water or surface oxygen passivated with hydrogen (Zn–OH–H, strongly H-bonded H_2_O), sitting between lattice Zn–O (∼530 eV) and higher-binding energy adsorbed carbonates/organics (∼532–533 eV) in the treatment with NaNO_3_ and especially CCM promotes partial hydrolysis, surface hydroxylation, and coordination of medium components to Zn sites, so this new 530.8–531 eV peak can reasonably be assigned to newly formed Zn–OH/chemisorbed H_2_O (or related strongly bound oxygenated species) on the degraded/modified ZIF-8 surface.^35^

The N 1s spectra of pristine ZIF-8 display two well-defined peaks at 399 and 400 eV, attributed to imidazolate C–N and amine/amide nitrogen functionalities, respectively.^36^ Notably, in WW- and CCM-treated samples, the higher binding energy N 1s peak shifts up to 401.5 eV and increases in width, consistent with significant incorporation of nitrogen-rich organics, ammonium ions, and bioderived functionalities at the MOF surface. Samples exposed to ASW and BHW show minor broadening and energy shifts in both O 1s and N 1s envelopes, suggesting moderate influence from dissolved minerals and cations. From the Zn 2p (Figure 3b) spectrum, it can be inferred that distinct peaks at binding energies of 1022 and 1045 eV in all the samples are ascribed to Zn^2+^ 2p_3/2_ and Zn^2+^ 2p_1/2_ electronic states with an energy difference of approximately 23 eV, confirming the presence of zinc in its anticipated oxidation state.^37^ In the high-resolution XPS, C 1s spectrum (Figure S5), the curve fitting reveals that peaks corresponding to C–C (284.7 eV) can be assigned to sp^2^ carbon in the imidazole ring, while C–N/C=N (286.2 eV) is assigned to carbon in the imidazole ring bonded to nitrogen. The extra C–O (287.7 eV) peak is assigned to surface oxidation present in all of the samples. In the case of the CCM, a ZIF-8 peak at 290.0 eV corresponding to carbonate formation was also observed. However, peaks around 288.5 to 288.8 eV, corresponding to the formation of O–C=O, were obtained in all the modified samples but absent in the pristine (activated) ZIF-8.

In addition to the observed shifts and modifications in C 1s, O 1s, and N 1s spectra, the XPS analysis revealed extra peaks corresponding to medium-specific ions in treated ZIF-8 samples. Spectra for Cl 2p in CCM and ASW indicate chloride adsorption on the ZIF-8 surface, directly tied to the presence of Cl^–^ in these solutions. Similarly, Na 1s peaks were detected in samples exposed to CCM, ASW, and WW, confirming sodium ion incorporation consistent with the medium’s composition. These observations collectively demonstrate that the surface chemical environment of ZIF-8 evolves dynamically depending on medium composition, with organic- and nutrient-rich environments driving pronounced shifts in oxygen and nitrogen functional groups, while the core framework maintains its primary coordination.

TEM imaging provides a structural correlate to these spectroscopic signatures (Figure 4). Pristine ZIF-8 consists of sharp, faceted nanocrystals with uniform contrast and spot-like SAED pattern characteristic of a highly crystalline framework (Figure 4a). After 7 day aging in the various media, particles become progressively rounded and aggregated; edges appear etched, and in CCM and WW particularly (Figure 4d), nanocrystals are coated with amorphous or low-contrast layers consistent with adsorbed organic/inorganic coronas (Figure 4d). In some cases (like simple waters ASW and BHW), SAED patterns exhibit weaker (Figure 4c,e) and more diffuse diffraction, indicative of a loss of long-range order. Taken together, the XAS, FTIR, XPS, and TEM data show that immersion in realistic waters drives ZIF-8 along a dissolution–restructuring–possibly corona-formation pathway: Zn–N bonds are partially hydrolyzed and replaced by Zn–O­(H) coordination, while the particle surface acquires a medium-specific organic/inorganic overlayer (eco-/biocorona). Medium composition controls the extent of these changes, with complex, nutrient-rich media (CCM/WW) pushing the material furthest from its pristine ZIF-8 state. These findings echo broader observations that MOF “water stability” is highly context-dependent and can break down in the presence of competing ligands and anions.^28^

**Figure 4:**
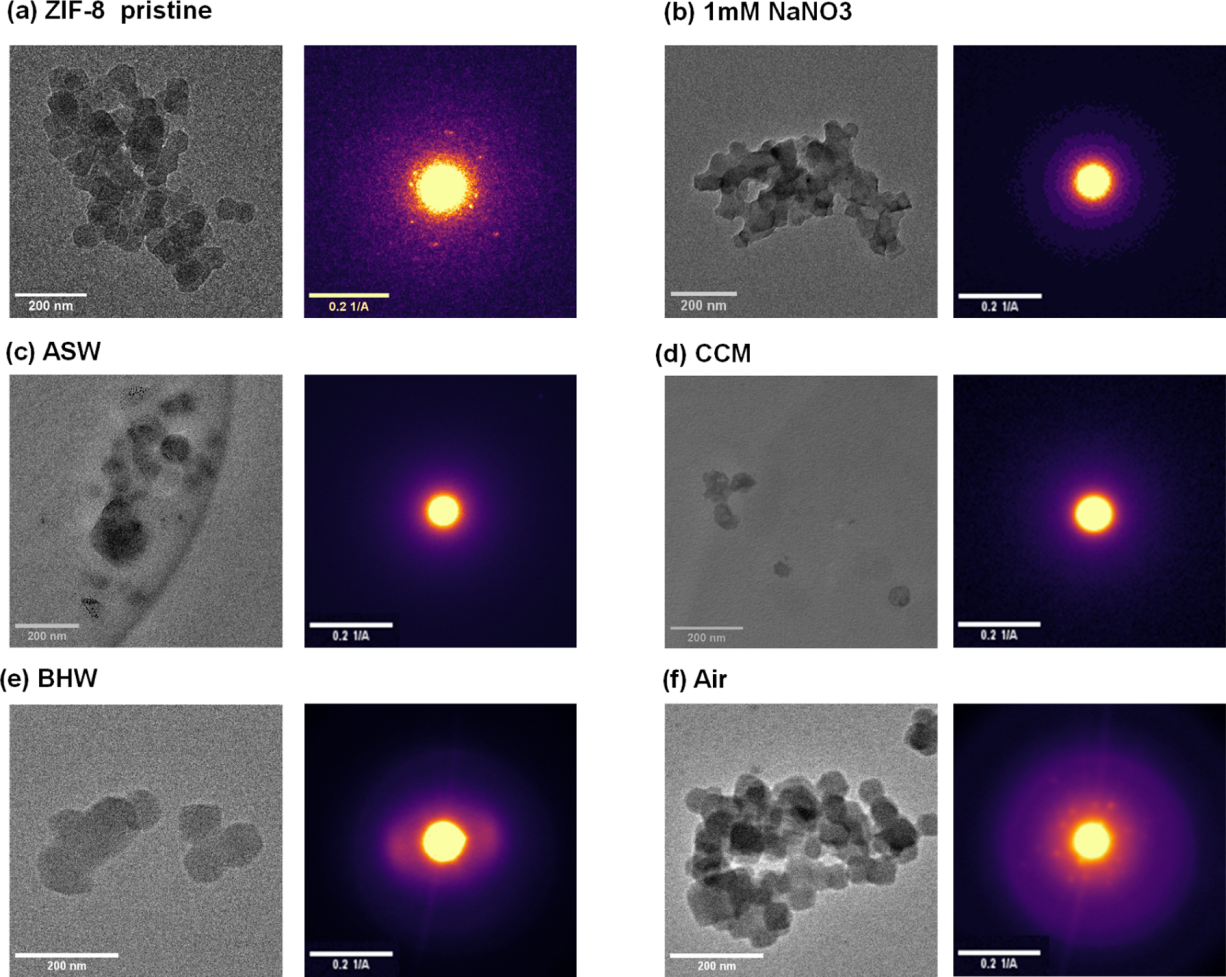
Representative bright-field TEM images (left in each pair) and corresponding selected-area electron diffraction (SAED) patterns (right in each pair) for ZIF-8 under different conditions: pristine as-synthesized ZIF-8, air-exposed ZIF-8, and ZIF-8 aged for 7 days in four aqueous media ((1 mM NaNO_3_, ASW, BHW, and CCM); order as labeled in the panel). Pristine and air-exposed samples show faceted nanocrystals that form dense aggregates and yield sharp, ring-like diffraction patterns characteristic of a highly crystalline sodalite framework. In contrast, medium-aged samples display progressively more rounded and coalesced aggregates, and their SAED patterns exhibit broader rings and increased diffuse scattering, indicating partial loss of long-range order and medium-dependent structural disordering of the ZIF-8. TEM scale bars: 200 nm; SAED scale bars: 0.2 Å^–1^.

To benchmark these aqueous-aged spectra against plausible Zn end-products, we compared the 7 day aged samples directly with Zn reference compounds ((Zn­(NO_3_)_2_, ZnO, ZnSO_4_, Zn_3_(PO_4_)_2_, and ZnF_2_) (Figure S6). In terms of Zn K-edge energy, all medium-aged ZIF-8 samples exhibit edge positions between those of pristine ZIF-8 and the hydrated Zn-salt references, indicating that aging does not alter the Zn valence state, which remains Zn^2+^. This spectroscopic picture closely parallels the Visual MINTEQ predictions (Figure S11), which indicate that Zn released from ZIF-8 in these matrices partitions into mixtures of hydroxo-, carbonato-, and phosphato-complexes and approaches saturation with respect to basic Zn carbonates and Zn phosphates rather than forming a single dominant Zn mineral, supporting the conclusion that secondary aqueous transformation produces families of disordered Zn–O/N species rather than a simple ZnO or Zn-salt end point.

Equilibrium speciation calculations with Visual MINTEQ v3.1 were used to contextualize the aqueous transformations (Figure S11) inferred from XAS, FTIR, XPS, and TEM. In the model, ZIF-8 was represented by its constituent Zn fraction, 25 wt % Zn, with total ZIF-8 loadings of 100 and 200 μg mL^−1^ used as thermodynamic upper-bound scenarios for release in ASW, 1 mM NaNO_3_, and BHW. Ionic composition and pH were taken from the experimentally used media. In 1 mM NaNO_3_ at pH ∼7, MINTEQ predicts that dissolved Zn is dominated by free Zn^2+^ with minor contributions from ZnOH^+^ and Zn­(OH)_2_(aq), and that the solution is undersaturated with respect to common Zn carbonate and hydroxide solids (negative saturation indices for ZnCO_3_(s), Zn­(OH)_2_(s), and hydrozincite). This is consistent with the relatively modest structural changes observed for NaNO_3_-aged ZIF-8: XAS retains a ZIF-8-like first shell, FTIR shows only weak broad O–H bands, and XPS reveals limited growth of Zn–O and carbonate components. Together, these data suggest that in simple, low-complexity electrolyte, the framework undergoes partial surface hydrolysis, releasing Zn^2+^ into solution but without extensive secondary precipitation.

In contrast, speciation in ASW and BHW is strongly controlled by inorganic ligands. The presence of carbonate, bicarbonate, and, in BHW, orthophosphate drives Zn into ZnCO_3_(aq), ZnHCO_3_^+^, and ZnHPO_4_^0^ complexes, and MINTEQ indicates that these systems approach or exceed saturation with respect to ZnCO_3_(s) and basic Zn carbonates while remaining close to saturation for Zn_3_(PO_4_)_2_-type phases under the upper-bound Zn loads. These predictions align with the experimental observations: FTIR spectra of ASW- and BHW-aged samples display pronounced carbonate bands and broad O–H features, XPS shows an increased contribution from Zn–O/Zn–OH and oxidized C–O/C=O components, and TEM/SAED reveals rounded particles with weakened diffraction, indicative of partial framework dissolution and reprecipitation of Zn–O/C phases at or near the surface. Importantly, MINTEQ does not explicitly represent the ZIF-8 lattice or its dissolution kinetics; it instead describes thermodynamic end points for Zn once released. Within that limitation, the model nevertheless captures the same qualitative hierarchy emerging from the experimental data: 1 mM NaNO_3_ provides little thermodynamic drive for secondary Zn solids, favoring mobile ionic Zn, whereas natural and saline waters (BHW, ASW) offer multiple pathways for Zn sequestration into carbonate and phosphate surface complexes or precipitates. This helps rationalize why XAS and TEM show more advanced restructuring in BHW and ASW than in 1 mM NaNO_3_ and provides a mechanistic bridge between our molecular-scale observations and longer-term fate scenarios in environmentally realistic waters.

To quantify “medium-dependent restructuring”, we combined time-resolved dissolved Zn release (Figure S15a–d; Table S13) with XPS-derived surface restructuring indices (Figure S15e; Table S14). Dissolution exhibited strong medium dependence in both early time release and late-time extent. At 72 h, dissolved Zn concentrations were highest in CCM (100 μg mL^–1^: 15.80 ± 1.40 μg mL^–1^; 200 μg mL^–1^: 27.80 ± 2.09 μg mL^–1^), followed by 1 mM NaNO_3_ (11.24 ± 1.34; 18.12 ± 2.36 μg mL^–1^) and then BHW (7.38 ± 1.03; 10.44 ± 2.10 μg mL^–1^), whereas ASW remained at a low dissolved-Zn plateau (2.76 ± 0.92; 5.29 ± 0.19 μg mL^–1^) (Table S13). At long times (336 h), dissolved Zn remained lowest in ASW (2.92 ± 0.46; 5.01 ± 0.24 μg mL^–1^), increased to moderate plateaus in BHW (11.93 ± 1.23; 13.59 ± 3.88 μg mL^–1^), and reached the highest long-time values in NaNO_3_ (14.86 ± 0.40; 19.58 ± 3.01 μg mL^–1^) (Table S13). Together these results provide a clear dissolution extent ranking: CCM > NaNO_3_ > BHW ≫ ASW. Complementarily, XPS showed marked surface conversion away from Zn–imidazolate chemistry. Relative to pristine ZIF-8 (O/N = 0.18), the strongest N depletion and O enrichment occurred in NaNO_3_ (O/N = 3.32; ΔN = −17.08 at %; ΔO = +22.48 at %), CCM (O/N = 2.56; ΔN = −16.48 at %; ΔO = +17.86 at %), and ASW (O/N = 2.41; Δ*N* = −16.13 at %; ΔO = +17.43 at %), whereas BHW and WW showed more moderate surface oxygenation (Table S14). Notably, dissolution extent and surface oxygenation do not rank identically (e.g., ASW shows strong surface oxygenation but low dissolved Zn), indicating that surface restructuring and dissolved release can decouple depending on medium chemistry.^25^

The combined dissolution and XPS data sets show that transformation is controlled by medium chemistry and that neither dissolved Zn release nor surface conversion alone captures the full trajectory. In ASW, dissolved Zn remains low while surface oxygenation and N depletion are large (Tables S13 and S14), consistent with a rapid shift toward O-rich surface environments under high ionic strength. Visual MINTEQ provides equilibrium speciation context indicating substantial inorganic complexation of dissolved Zn in seawater-like chemistry (e.g., chloride/sulfate/hydroxide complexes), which is consistent with the observed low-dissolved-Zn plateau (Figure S11). In BHW, dissolution proceeds more gradually to a higher plateau, consistent with carbonate/bicarbonate and trace phosphate providing competing sinks and saturation control; dissolved organic matter can buffer dissolved Zn through complexation and is represented in MINTEQ as a humic interaction term as a bounding assumption. In 1 mM NaNO_3_, the comparatively simple electrolyte background permits a larger dissolved Zn pool and strong surface oxygenation, consistent with a reduced competition from complexing ligands. Finally, CCM produces the greatest dissolved Zn release and strong surface restructuring, aligning with published evidence that ZIF-8 degrades in cell media/serum and phosphate-containing physiological buffers, where bicarbonate/phosphate chemistry and biomolecular interactions can accelerate transformation relative to simple water.^38^ Together, these quantified trends support the central conclusion of the paper: hazard should be interpreted as an outcome of the transformation trajectory rather than pristine composition. The media that promote strong Zn release and/or strong O-rich surface conversion are those most likely to generate transformed Zn pools that persist into the biotic tier and contribute to toxicity, consistent with our in vivo localization/speciation evidence.

### Tertiary Transformation in *D. magna*

To examine tertiary transformation in *D. magna*, we conducted μ-XRF mapping and Zn K-edge XANES on an unexposed control and two daphnids exposed for 12 h to ZIF-8 that had been preaged in BHW for 7 days (exposure concentration to 10 μg mL^–1^ and 25 μg mL^–1^). We therefore interpret the detected Zn species as the outcome of sequential transformation, with initial abiotic restructuring in the BHW medium, followed by additional biological processing after ingestion. Consistent with this, the Zn K-edge XANES spectra collected from exposed daphnids differ not only from the control but also from the 7 d BHW-aged ZIF-8 reference, indicating further in vivo respeciation beyond that produced in the medium alone. Whole-organism μ-XRF mapping reveals that ingestion by *D. magna* drives ZIF-8 (preaged in BHW for 7 days) along a tertiary transformation pathway that is distinct from both gas- and solution-phase aging (Figures 5, S9). These in vivo μ-XRF and Zn K-edge XANES measurements were obtained after a 12 h exposure of neonate *D. magna* to BHW-aged ZIF-8 at 10 or 25 μg mL^–1^. During the 12 h exposure, neonates at 10 μg mL^–1^ remained fully mobile, while at 25 μg mL^–1^, neonates showed impaired swimming but were not visibly dead prior to isolation for synchrotron measurements. This short exposure duration was selected to capture early in vivo respeciation while retaining intact organisms for XRF mapping and XAS measurements. Resolving the exact Zn speciation of this dilute, the biologically active BHW exposure system at the 12 h time point would require a dedicated high-sensitivity synchrotron kinetics experiment, beyond the scope of this work; accordingly, we interpret the current data set as evidence for sequential abiotic-plus-biotic transformation and identify early time in vivo speciation kinetics as an important next step.

**Figure 5:**
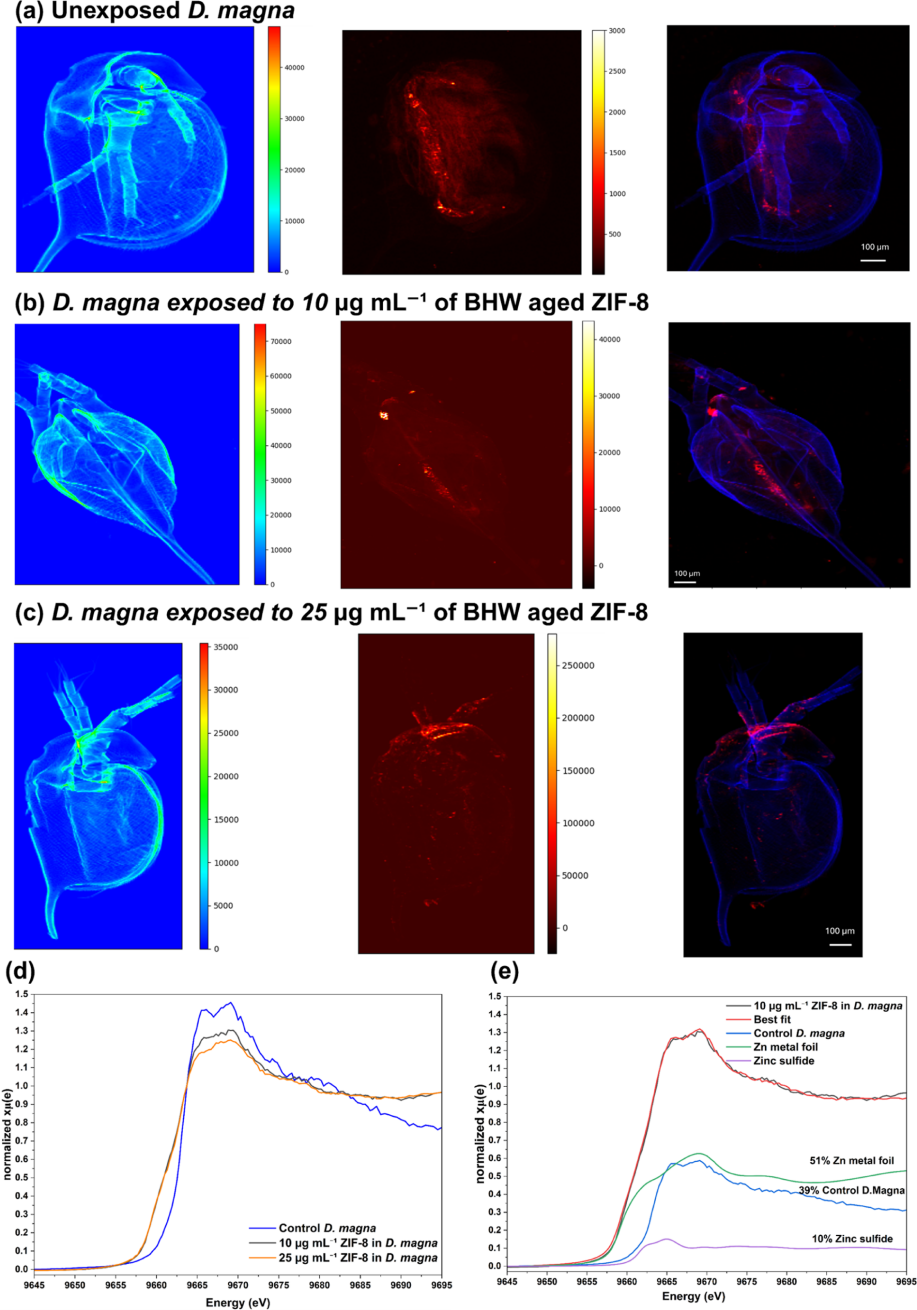
Tertiary transformation and in vivo respeciation of ZIF-8 in *D. magna*. Micro–X-ray fluorescence (μ-XRF) maps of Ca/Zn overlay (right), Ca (left), and Zn (middle) individual elements collected on a (a) whole unexposed *D. magna* and on *D. magna* exposed to the (b) 10 μg mL^–1^ and (c) 25 μg mL^–1^ BHW-aged ZIF-8 media (scale bars: 100 μm). Ca delineates the carapace and appendages, while Zn is concentrated in the digestive tract and selected internal regions. The intensity bars depict the range of elemental XRF counts; these demonstrate that the accumulation of Zn in *D. magna* is progressively higher with higher concentrations of Zn in the BHW media, indicating dietary uptake and internal redistribution of ZIF-8-derived Zn. The control organism also contains endogenous Zn but in much lower levels than those grown in ZIF-8 treated BHW media. (d) Normalized Zn K-edge XANES spectra of Zn-rich regions inside control *D. magna* and those that were exposed to the 10 μg mL^–1^ and 25 μg mL^–1^ ZIF-8 in BHW. The spectrum measured on control differs strongly from both 10 μg mL^–1^ and 25 μg mL^–1^ BHW-aged ZIF-8, demonstrating complete respeciation of the Zn environment during biological processing. (e) Linear combination fitting of the Zn K-edge XANES spectrum collected on *D. magna* exposed to 10 μg mL^–1^ BHW-aged ZIF-8 using Zn metal foil, unexposed *D. magna*, and zinc sulfide as reference components.

Ca maps delineate the carapace, appendages, and shell glands, while Zn maps show intense, spatially heterogeneous signal concentrated in the digestive tract and selected internal regions (Figure 5a, b, c). In the Ca/Zn overlay, Zn hotspots are clearly superimposed on Ca-rich anatomical structures rather than appearing as a diffuse signal, as would be expected for fully dissolved Zn. Thus, the species detected in *D. magna* should not be interpreted as arising exclusively in the medium or exclusively in the organism but rather as products of a cascade in which medium-aged ZIF-8 is subsequently modified during gut-associated biological processing. This pattern is consistent with ZIF-8-derived material (and/or its aqueous transformation products) adhering to the carapace and accumulating along mineralized and gut-associated tissues, rather than remaining solely as freely dissolved species in the medium. The Zn XRF signal increases progressively from the control group (no exposure to ZIF-8) to organisms exposed to 10 μg mL^–1^ and 25 μg mL^–1^ ZIF-8 aged in BHW. In control *D. magna*, the maximum Zn signal is approximately 3k XRF counts, whereas exposure to 10 μg mL^–1^ ZIF-8 in BHW results in roughly 40k counts, and exposure to 25 μg mL^–1^ leads to about 250k counts. This pattern demonstrates a concentration-dependent accumulation of Zn in *D. magna*, consistent with dietary uptake and internal redistribution of Zn originating from ZIF-8. Zn K-edge XANES collected from Zn-rich regions in the daphnids differs markedly from control *D. magna*, pristine, and BHW-aged ZIF-8 (Figures 5d, S7). The characteristic multipeak ZIF-8 fingerprint is absent; instead, the in vivo spectrum is featureless beyond the edge, with a broadened white line and strongly damped oscillations. The edge energy position shifts to lower energy, indicating a change in the Zn valence state and the postedge region lacks the oscillatory structure seen in either pristine ZIF-8 or the 7 d BHW-aged material, indicating that the tetrahedral Zn–imidazolate environment has been completely replaced by new, more disordered coordination motifs. The spectra collected from daphnids exposed to BHW-aged ZIF-8 are largely comparable, with only minor variations in white-line intensity. Notably, the organism exposed to 25 μg mL^–1^ ZIF-8 shows a reduced white-line intensity (Figures 5d, S7). This decrease arises from self-absorption effects, which become significant at higher Zn concentrations. Consistent with this interpretation, the transmission signal for the 25 μg mL^–1^ sample displayed a strong edge jump (∼0.1), and direct comparison of the fluorescence and transmission spectra measured on the same specimen confirms that the XAS spectrum from the 25 μg mL^–1^ BHW-aged ZIF-8 exposure is affected by self-absorption (Figure S7).

To distinguish endogenous Zn present in unexposed *D. magna* from Zn originating from transformed ZIF-8 in exposed organisms, we performed linear combination fitting (LCF) analysis of the Zn K-edge XANES spectra using a suite of reference compounds. In total, approximately ten standards were tested (Figure S9), and excellent fits were obtained using the Zn K-edge XANES profiles of unexposed *D. magna*, Zn metal foil, and zinc sulfide. We note that an equally good fit was achieved when zinc phosphate (Zn_3_(PO_4_)_2_) was used in place of zinc sulfide (Figure S8), and the two solutions could not be differentiated within the accuracy of the method; we therefore refer to this contribution conservatively as a Zn–O/P/S-type transformed pool rather than uniquely assigning a single mineral phase.

Ex situ TEM of particulate material recovered from depuration water provides structural corroboration of this in vivo respeciation (Figure S12). High-Angle Annular Dark-Field Scanning Transmission Electron Microscopy (HAADF-STEM) imaging shows irregular, worm-like aggregates with bright contrast regions indicative of high-Z elements embedded in a lower-Z organic matrix (Figure S12). STEM–Energy-dispersive X-ray spectroscopy (EDS) mapping confirms that these bright domains are enriched in Ca and colocalize with P, S, Mg, and O, while C forms a more diffuse background. The signal mapping of Zn–Kα shows the localization at some areas where faceted features are enveloped inside the Ca- and O-rich particles. The associated EDS spectrum shows recognizable peaks for Zn–Kα, albeit slightly weak, together with Ca, P, and S, consistent with the presence of mixed Zn–Ca phosphate/sulfate phases or Zn bound to phosphorus- and sulfur-containing biomolecules. No regions exhibit the homogeneous, sharp-edged contrast typical of pristine ZIF-8 nanocrystals imaged under identical conditions and selected-area diffraction from these aggregates is dominated by diffuse halos rather than discrete rings (not shown), confirming extensive loss of long-range order.

These observations together demonstrate that ingestion by *D. magna* leads to complete in vivo respeciation of ZIF-8 rather than simple physical accumulation of intact nanoparticles. At the organism scale, co-localization of Zn with Ca in the carapace and gut suggests at least two Zn sinks: (i) adsorption or incorporation of ZIF-8-derived Zn into Ca-rich exoskeletal structures and (ii) retention or transformation of Zn within the digestive system. The former is consistent with the known capacity of crustaceans to sequester metals in the exoskeleton and shell glands,^39^ while the latter implicates the gut microenvironment—low pH, digestive enzymes, dissolved organic matter, and microbiota—as a key driver of MOF breakdown/transformation.

The XANES data indicate that the Zn coordination environment inside *D. magna* is no longer recognizable as ZIF-8 or as the partially hydrolyzed Zn–O­(H)/Zn–N states observed after abiotic aqueous aging. Instead, the in vivo spectrum resembles that of metallic zinc and a disordered mixture of Zn–S and Zn–O/P environments, consistent with Zn bound to phosphates, carboxylates, and other bioligands. This is a more advanced transformation state than any reached in the abiotic media, highlighting that biological milieus open reaction channels—such as enzymatic hydrolysis, ligand exchange with intracellular phosphates and organics, and biomineralization—that are inaccessible in simple electrolyte or natural waters.

At the particle scale, HAADF–STEM/EDS mapping reinforces this interpretation. The close spatial overlap of Zn with P and Ca suggests formation of zinc-rich biominerals, likely mixed Zn–Ca phosphate or Zn-phosphate/carbonate phases, while the co-occurrence of Zn and S points to additional complexation by sulfur-containing biomolecules (e.g., thiol-rich proteins or small molecules). The absence of crystalline diffraction from these aggregates indicates that the original ZIF-8 lattice has been fully dismantled, leaving behind amorphous or nanocrystalline secondary phases embedded in an organic matrix. In other words, by the time material is excreted, the MOF has traversed a full transformation trajectory from a crystalline Zn–imidazolate framework to biologically processed Zn–O/P/S complexes. HAADF–STEM/EDS of pristine and BHW-aged ZIF-8 (Figure S10) shows Zn and N colocalized within faceted particles, with only modest O enrichment, whereas excreted aggregates from *D. magna* display Zn intimately associated with P, S, and Ca (Figure S12). This clear shift in elemental neighborhood from Zn–N/O to Zn–O/P/S/Ca provides direct nanoscale evidence that the tertiary, in vivo transformation produces new Zn-rich biominerals and complexes that cannot be generated by abiotic aging alone.

This behavior mirrors our earlier findings for UiO-66, where a Zr–carboxylate framework that appeared structurally intact after 7 d in water underwent complete conversion to disordered Zr–hydroxide species inside *D. magna*.^6^ In that system, in vivo transformation was temporally aligned with delayed but pronounced reproductive toxicity. The ZIF-8 data fit the same conceptual model: abiotic exposures “pre-condition” the material, but the decisive transformation steps occur in vivo, generating new Zn species with altered solubility, bioavailability, and interaction with biological targets. This provides a plausible mechanistic bridge between the modest chronic toxicity observed at low (discussed in following section), nominally sublethal ZIF-8 concentrations and the extensive structural changes documented here. Whole-organism μ-XRF provides organism-level localization of Zn but cannot unambiguously assign signal to specific organs (e.g., gut lumen vs epithelium) or to intracellular compartments. Future work will therefore use microtome/cryo-sectioning of exposed *D. magna,* followed by μ-XRF/μ-XAS mapping of cross sections to quantify Zn partitioning across carapace, gut lumen, and gut epithelium, enabling a more definitive assessment of retention versus tissue translocation.

### Ecotoxicological Implications of Hierarchical Transformation of ZIF-8 in *D. magna*

Quantitative predicted environmental concentrations (PECs) for MOFs such as ZIF-8 are not yet established, because production volumes and release rate during use remain poorly constrained. In the broader engineered-nanomaterial literature, probabilistic material-flow and fate models for widely used materials commonly predict regional surface water concentrations in the ng L^–1^ to low μg L^–1^ range while emphasizing that localized point sources (e.g., wastewater treatment plant discharges and industrial outfalls) and episodic releases can yield substantially higher short-term concentrations than regional averages.^15^ We therefore used a tiered experimental window: (i) low-dose chronic testing at 0.10 μg mL^–1^ (OECD TG 211 end-point framework) to capture sustained sublethal impacts and (ii) an acute range down to 0.01 μg mL^–1^ to bracket environmentally plausible concentrations while still resolving concentration–response behavior for hazard characterization.

Neonates exposed to pretransformed ZIF-8 for 24 and 48 h showed clear, sigmoidal concentration–response relationships for both mortality (organism with no movement) and immobilization (organism unable to swim with 15 s of gentle agitation) (Figure 6a). Immobilization was used as the primary acute end point in accordance with OECD Test Guideline 202, and the resulting concentration–response curves were used to derive 24 and 48 h EC_50_ values and the 48 h EC_10_ subsequently applied in the chronic assay (Figure 6a). Immobilization increased sharply with concentration and, at 48 h, was significantly higher than the control from 0.05 μg mL^–1^ onward (0.05 and 0.10 μg mL^–1^, *p* <0.05; 0.50 μg mL^–1^, *p* <0.01; 1–10 μg mL^–1^, *p* <0.001), whereas 0.01 μg mL^–1^ did not differ from the control (*p* >0.05), demonstrating a pronounced, time-dependent intensification of effect. Control immobilization remained ≤10%, confirming that the tests met OECD validity criteria and that the observed responses were treatment driven. The OECD immobilization test was designed for soluble chemicals in which immobilization is a good proxy for mortality, but in the case of nanomaterials, physical interaction of particles with the daphnid exoskeleton (carapace) can result in limited mobility in the initial 15 s after gentle agitation but the organisms remaining alive as evidenced by subsequent vertical migration of the daphnids. Dead daphnids are unable to swim upward, while some daphnids that appeared immobilized in the initial 15 s post agitation subsequently showed vertical migration upward. Thus, as an additional end point beyond the OECD 202 requirement, we also measured mortality as the total loss of vertical (upward) migration to more fully characterize hazard and to enable LC_50_ determination (Figure 6b). At 24 h, mortality was negligible at concentrations ≤0.1 μg mL^–1^ but increased sharply between 0.1 and 1 μg mL^–1^, approaching ∼70–80% at 10 μg mL^–1^. By 48 h, mortality remained absent up to 0.50 μg mL^–1^ (*p* >0.05 versus control) but increased sharply at ≥1 μg mL^–1^, where mortality was significantly higher than the control (1 and 10 μg mL^–1^, *p* <0.001; 5 μg mL^–1^, *p* <0.01), highlighting clear time-dependent toxicity. Four-parameter Hill fits (eq 1) yielded 24 and 48 h LC_50_ values in the low μg mL^–1^ range. As expected for a functional end point, the 48 h EC_50_ for immobilization was lower than the corresponding LC_50_, consistent with immobilization capturing earlier sublethal impairment of swimming that precedes overt mortality. Immobilization was used as the primary acute end point (OECD TG 202), giving a 48 h EC_50_ of 0.525 μg mL^–1^ and a 48 h EC_10_ of 0.10 μg mL^–1^; the 48 h EC_10_ was used to select the chronic exposure concentration (0.10 μg mL^–1^).

**Figure 6:**
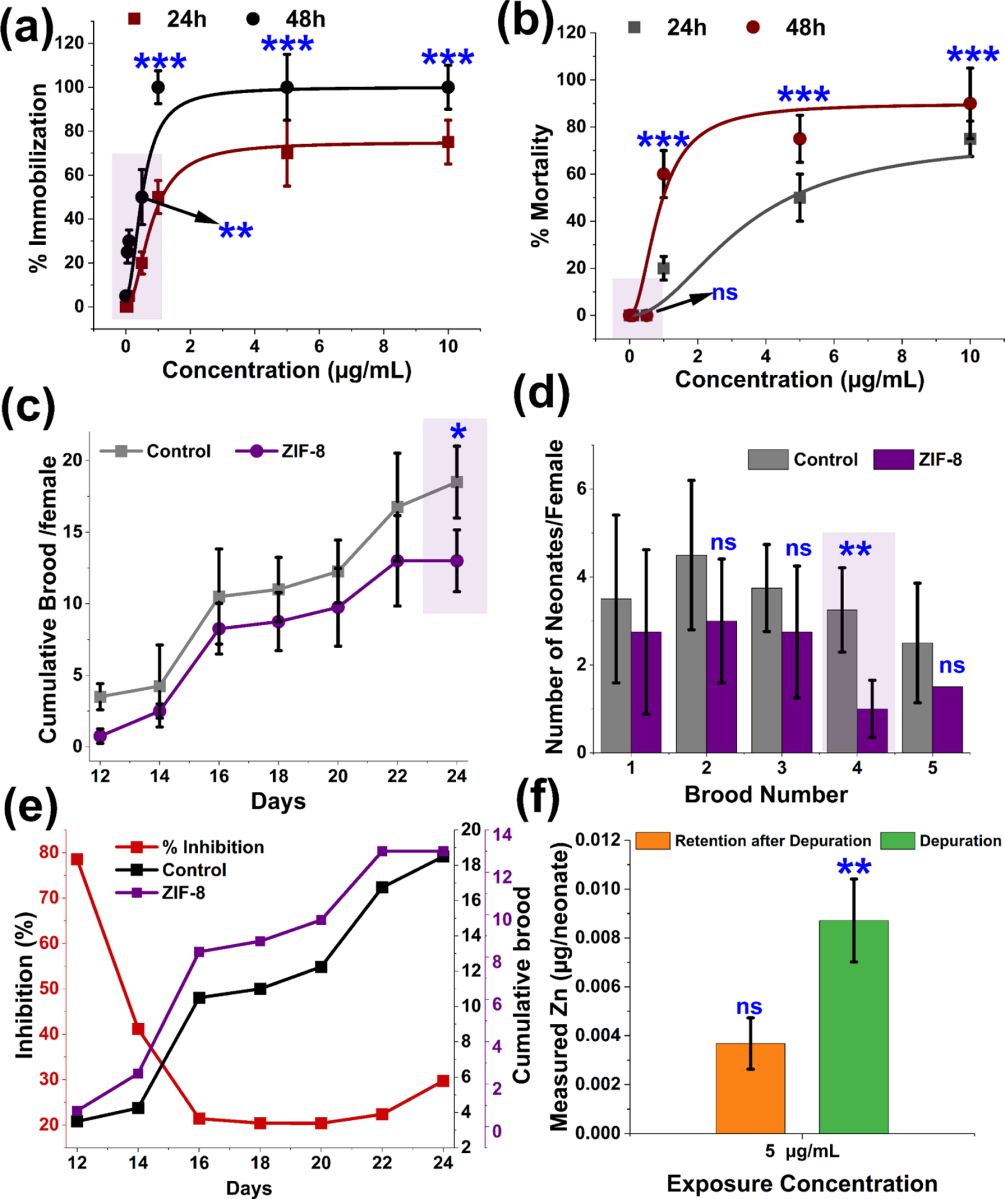
Acute toxicity, chronic reproduction, and short-term uptake/depuration of ZIF-8 in *D. magna*. (a) 24 and 48 h immobilization under the same exposure conditions; 48 h data were used to derive EC_50_ and EC_10_ values. (b) 24 and 48 h mortality of neonates exposed to ZIF-8 (0–10 μg mL^–1^); solid lines show four-parameter logistic fits. (c) Cumulative brood size per female over 24 days in controls and at 0.1 μg mL^–1^ ZIF-8. (d) Number of neonates per brood (broods 1–5) in control and ZIF-8 treatments. (e) Reproduction inhibition (%) and cumulative brood size over time. (f) Zn retained after depuration and Zn depurated from neonates after 12 h exposure to 5 μg mL^–1^ ZIF-8, expressed as μg Zn per neonate. Data are mean ± SD. For panels a and b, *n* = 4 beakers with 5 neonates per beaker; for panels c and d, *n* = 4 females; for panel f, *n* = 3 biological replicates. Significance symbols indicate unpaired two-tailed Student’s *t* tests versus control: ns (not significant), *p* >0.05; *p <0.05; **p <0.01; ***p <0.001.

To probe longer-term effects, a 24 day chronic exposure (OECD TG 211-based, extended to capture delayed broods) was conducted at 0.10 μg mL^–1^ pretransformed ZIF-8 (diluted from stock of 100 μg mL^–1^ ZIF-8 in BHW), corresponding to the 48 h EC_10_ from the acute assay. Cumulative brood size per female increased over time in both treatments but remained consistently lower in the ZIF-8 group (Figure 6c). Differences were small in the early phase (days 12–14), but from approximately day 16 onward, the gap widened, with control organisms producing on average 2–4 additional neonates per female by day 24; at the final time point, cumulative brood size was significantly lower in the ZIF-8 treatment than in the control (*p* <0.05). Brood-resolved data (Figure 6d) show that the first brood size was only modestly affected, whereas mean neonate numbers per brood were generally lower in the ZIF-8 treatment for later broods; this difference was most pronounced and statistically significant for brood 4 (control: 3.25 ± 0.96 versus ZIF-8:1.00 ± 0.65 neonates per brood, *p* <0.01), while the other brood-wise comparisons were not significant (*p* >0.05). When expressed as percentage inhibition relative to the control (Figure 6e), reproductive inhibition started around ∼40% at the onset of reproduction and then stabilized at ∼20–30% from midtest onward, even though adult survival remained high, with only one female death in ZIF-8 exposed daphnid on day 18. The overlaid cumulative brood curves emphasize that this persistent, moderate inhibition translates into a substantial reduction in total offspring produced over a full reproductive cycle. Depuration–retention experiments with 24 h neonates at 5 μg mL^–1^ ZIF-8 showed measurable accumulation of Zn during exposure and incomplete elimination during the subsequent 12 h depuration period (Figure 6f). Following exposure, neonates depurated 8.71 ± 1.70 × 10^–3^ μg Zn per neonate over 12 h, significantly higher than the unexposed control (2.61 ± 0.57 × 10^–3^ μg Zn per neonate, *p* <0.005), but still retained 3.68 ± 1.05 × 10^–3^ μg Zn per neonate after 12 h of depuration; retained Zn was not significantly different from the control (*p* >0.05), whereas the depurated fraction remained significantly higher than the retained fraction (*p* <0.005), indicating partial elimination but incomplete clearance of ZIF-8-derived Zn from the digestive tract and carapace. These values are fully consistent with the μ-XRF/XAS evidence for Zn localization in the gut and carapace and with the formation of Zn–P/S/Ca-rich aggregates in depuration media. Representative micrographs on Day 24 (extended to capture the delays in brood release due to ZIF-8 exposure) (Figure S13) revealed additional sublethal effects at 0.1 μg mL^–1^ ZIF-8. Exposed adults were slightly smaller than controls (mean body length 4.0 ± 0.5 mm vs 4.6 ± 0.3 mm for controls, *n* = 3 per group) and two females exhibited partial loss of the posterior tail spine. Although overall adult survival in the ZIF-8 treatment remained within OECD validity limits, one female died on Day 18. These qualitative observations suggest that chronic exposure to low ZIF-8 levels can subtly affect somatic growth and morphology, complementing the quantitative evidence for reduced reproductive output.

### Fractionated Acute Toxicity Separates Dissolved vs Particle-Associated Contributions

To directly test whether acute effects are dominated by dissolved Zn species or by particle-associated transformation products, we compared 48 h immobilization in *D. magna* for three fractions derived from the same 7 day aged suspension: filtrate-only, washed particles/precipitates, and the whole aged suspension (Figure S14c; Table S15). Nominal concentrations refer to the parent whole aged suspension, and equal exposure volumes of filtrate and washed/resuspended particle fractions were used to ensure consistent handling and comparability across the fractions. The washed particle/precipitate fraction and whole aged suspension produced higher immobilization than the particle-free filtrate across the tested concentration range, indicating that toxicity is not attributable to dissolved species alone and implying that particle-associated transformed Zn pools are major contributors. The fractionation assay provides a direct functional test of whether dissolved Zn species alone can explain the acute effects. Because nominal concentrations were defined relative to the same parent whole aged suspension and equal exposure volumes were used for filtrate, washed particles/precipitates, and whole suspension, the observed potency differences between fractions cannot be attributed to differences in exposure volume or handling. Instead, the higher immobilization observed for washed particles/precipitates and the whole aged suspension relative to the particle-free filtrate supports a dominant contribution from particle-associated transformation products (secondary phases and/or surface-transformed particles), consistent with the transformed Zn pools indicated by the in vivo speciation/localization data sets.

To disentangle the roles of the individual ZIF-8 constituents, we first assessed the acute toxicity of dissolved Zn using Zn­(NO_3_)_2_·6H_2_O at Zn-equivalent levels matching the ZIF-8 exposures (0.01–10 μg mL^–1^) ZIF-8; 0.00287–2.873 μg mL^–1^ Zn_eq; Figure S14a. Immobilization and mortality remained negligible up to 0.0287 μg mL^–1^ Zn_eq (≤0.10 μg mL^–1^ ZIF-8-equivalent) and only increased sharply at ≥0.144 μg mL^–1^ Zn_eq (≥0.50 μg mL^–1^ ZIF-8-equivalent). In contrast, ZIF-8 itself induced substantial 48 h immobilization and mortality at considerably lower Zn_eq, demonstrating that dissolved Zn^2+^ alone cannot account for the observed acute effects and that particulate ZIF-8 and/or its transformation products provide additional toxic pressure. Visual MINTEQ speciation calculations (Figure S11) support this interpretation, predicting that in BHW and ASW, most Zn released from ZIF-8 is partitioned into hydroxo-, carbonato-, and phosphato-complexes and frequently approaches saturation with respect to basic Zn carbonates and Zn phosphates, rather than existing predominantly as free Zn^2+^. Consistently, Zn K-edge XANES comparison with Zn references (Zn­(NO_3_)_2_, ZnO, ZnSO_4_, ZnS, Zn_3_(PO_4_)_2_, ZnF_2_; Figure S6) shows that 7 day-aged ZIF-8 spectra do not converge on any single crystalline reference. Instead, observed spectral changes indicate increased local disorder around Zn atoms upon exposure to various liquid environments. To probe the contribution of the organic linker, we also exposed *D. magna* to mIm at linker-equivalent concentrations corresponding to 0.01 μg mL^–1^–10 μg mL^–1^ ZIF-8; across this range, no immobilization or mortality was observed up to 48 h, indicating that free mIm is essentially nontoxic under the test conditions. Taken together, these component-specific assays, speciation modeling, and XAS data indicate that the acute toxicity of ZIF-8 is dominated by Zn—present as a dynamic mixture of dissolved complexes, poorly crystalline Zn-rich phases and residual (transformed) particulates—rather than by the imidazolate linker or by simple Zn^2+^ release alone.

The low μg mL^–1^ LC_50_ and EC_50_ values place ZIF-8 among the more toxic Zn-containing particulates tested in *D. magna*, broadly comparable to the effects reported for nanoscale ZnO or soluble Zn salts in standard immobilization assays, where 48 h EC_50_ values often lie in the tens to hundreds of μg L^–1^ range depending on water chemistry and particle size. Previous studies have shown that ZnO nanoparticle toxicity in *D. magna* arises from a combination of dissolved Zn^2+^ and nanoparticle–organism interactions,^40,41^ including oxidative stress and physical interference with the filtering apparatus. Similar mixed-mode behavior is likely for ZIF-8: aqueous aging data demonstrate that the framework undergoes partial hydrolysis and develops Zn–O­(H) coordination in natural and synthetic waters, yielding both dissolved Zn species and partially transformed particles. These species act together to drive the rapid immobilization and mortality observed within 48 h. The higher sensitivity of immobilization relative to mortality is consistent with classical *D. magna* toxicology, where impaired swimming due to physical binding of particles to the appendages can interfere with the use of immobilization as a measure of mortality, with reduced feeding considered a better proxy for acute impacts that precedes death.^42^ Importantly, because gas-phase aging leaves ZIF-8 largely unchanged, the acute effects seen here can be attributed primarily to transformations occurring in the test medium and within the organisms, rather than to prior atmospheric degradation.

At the chronic exposure level (EC_10_), ZIF-8 exerts a clear, persistent inhibition of reproduction. The delay and reduction in later broods suggest cumulative, sublethal impacts on energy allocation and reproductive physiology: resources that would normally be invested into offspring may instead be diverted to detoxification, repair or maintenance in the presence of a continuous, low-level Zn and linker load. Comparable patterns—minimal effects on adult survival but substantial reductions in reproduction—have been reported for several metal-oxide and metal–organic nanomaterials in *D. magna* and other cladocerans, where chronic end points are often more sensitive than acute immobilization. In our previous work on UiO-66, a structurally distinct Zr-MOF, we similarly observed that in vivo transformation to disordered Zr–hydroxide phases in the gut coincided with chronic reproductive toxicity at concentrations close to acute EC_10_ values.^6^

Dissolution measurements in BHW show that ZIF-8 releases Zn slowly but persistently over time (Figure S15a). Notably, dissolved Zn in BHW approaches a plateau (Figure S15a), consistent with a solubility/speciation ceiling imposed possibly by a carbonate/phosphate chemistry, such that dissolved Zn does not scale linearly with the available ZIF-8 mass.^43,44^ No dissolved Zn was detected over the first 5 h at either loading, after which concentrations increased steadily to ∼5.6 μg mL^–1^ (200 μg mL^–1^) and ∼3.0 μg mL^–1^ (100 μg mL^–1^) by 48 h and then approached plateaus of ∼13.6 and ∼11.9 μg mL^–1^ by 336 h. In fractional terms, this corresponds to ∼6–7% dissolution at 200 μg mL^–1^ and ∼12% at 100 μg mL^–1^, indicating that dissolution is constrained by a solubility/speciation ceiling rather than by the available solid mass. This behavior is consistent with Visual MINTEQ predictions that dissolved Zn in BHW is strongly complexed as hydroxo-, carbonato-, and phosphato-species and can approach saturation with respect to secondary Zn carbonates/phosphates, and with XANES/EXAFS evidence for disordered Zn–O/N environments rather than complete loss of the framework. Importantly, these partial but sustained releases help explain why ZIF-8 produces marked acute and chronic effects in *D. magna*: over ecologically relevant time scales, organisms are exposed to a dynamic mixture of residual particles, dissolved Zn complexes, and secondary Zn-rich phases, rather than to an inert, fully crystalline MOF.

Here, the hierarchical transformation framework helps rationalize why a nominally low exposure can still impair reproduction (Figure 7). In the aqueous tier, ZIF-8 is partially hydrolyzed and coated with an eco-corona of carbonates, phosphates, and organic macromolecules; in the tertiary, organism tier, ingestion converts the framework into Zn-rich biominerals and complexes that colocalize with Ca and P in the carapace and gut.^45–47^ These transformed phases are likely to have different solubility and interaction profiles than both pristine ZIF-8 and simple Zn^2+^, potentially interfering with mineralization, osmoregulation, or endocrine signaling over time. Because *D. magna* is a keystone grazer in freshwater food webs, even a 20–30% reduction in brood production at environmentally realistic concentrations could translate into significant population-level consequences. The uptake–depuration data provide a mechanistic bridge between structural transformation and toxicity. The fact that total Zn per neonate remains elevated after 12 h depuration indicates that a fraction of ZIF-8-derived Zn is sequestered into slowly exchangeable pools rather than being readily excreted. This interpretation is supported by micro-XRF/XANES and HAADF-STEM/EDS, which show Zn associated with P, S, and Ca in internal tissues and in excreted aggregates. Such sequestration can perturb Zn homeostasis: *D. magna* relies on tight regulation of essential metals, and disruption of this balance is known to impair moulting and reproduction even in the absence of high mortality.^20^

**Figure 7:**
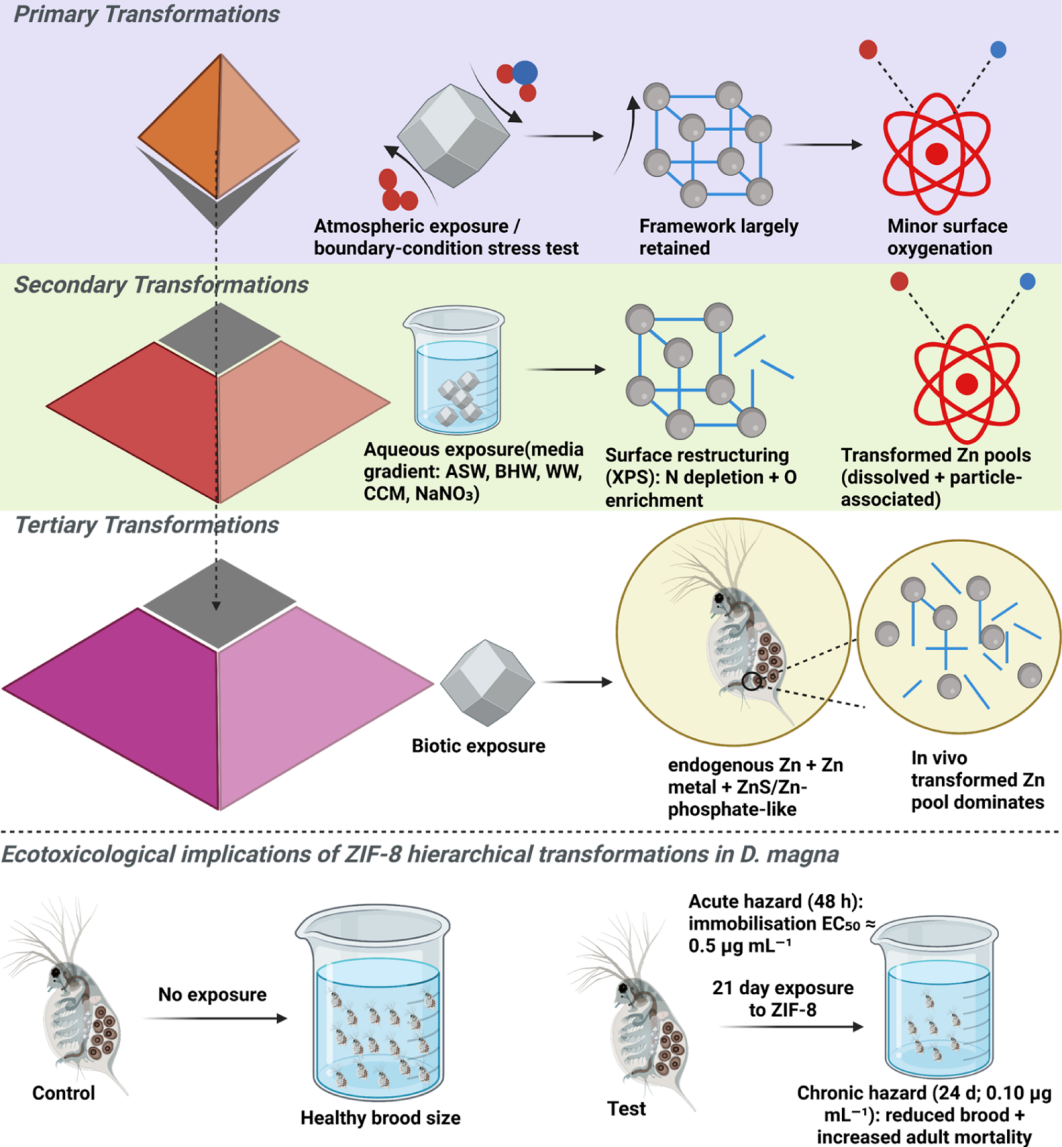
Evidence-anchored schematic of hierarchical ZIF-8 transformation and hazard across an exposure cascade (air → water → biota). The schematic summarizes outcomes supported by the experimental data sets (dissolution/XPS, in vivo μ-XRF and Zn K-edge XANES/LCF, and ecotoxicology including fractionated toxicity). Created in BioRender. Chakraborty, S. (2026) https://BioRender.com/x7f0rcy.

Our BHW results highlight that ZIF-8 hazard is likely to be context-dependent because natural freshwater chemistry (carbonate/bicarbonate alkalinity, phosphate availability, ionic strength, and natural organic matter) can shift Zn between dissolved and particle-associated forms and promote secondary Zn phases. In complex ecosystems, this has two key implications. First, even when dissolved Zn is moderated by precipitation/complexation, particle-associated Zn and transformed phases may remain bioavailable through ingestion and gut processing in filter feeders, which can alter exposure routes relative to dissolved-only scenarios. Second, once accumulated in prey organisms, metal-containing particulates can, in principle, be transferred through food webs; trophic-transfer studies with metal-based nanoparticles (including Zn-containing systems) show that body burdens are not solely explained by dissolved ions and that dietary exposure can be an efficient route to predators.^48,49^ Finally, we note that natural waters commonly contain cocontaminants (metals, nutrients, organic pollutants). Because MOFs and their transformation products can interact with other solutes via adsorption/complexation, mixture contexts may modify both contaminant transport and biological effects, warranting future cascade studies that explicitly incorporate realistic chemical mixtures and trophic transfer designs.^49,50^

Taken together, the acute, chronic, and depuration experiments demonstrate that toxicity of ZIF-8 to *D. magna* emerges from its full hierarchical transformation trajectory rather than from any single step. Gas-phase exposure leaves the MOF intact; aqueous aging initiates hydrolysis and possible eco-corona formation; and biological processing completes respeciation into Zn-rich biominerals and complexes, some of which are retained following depuration. This multitier perspective clarifies why ZIF-8 can show both relatively strong acute toxicity at μg mL^–1^ concentrations and more subtle but ecologically meaningful reproductive (chronic) effects at its acute EC_10_. It also underscores a broader design implication for MOFs in aquatic environments: assessments based solely on pristine materials or short-term dissolution tests are likely to underestimate long-term, life-cycle risks unless in vivo transformation pathways and chronic end points in sentinel species such as *D. magna* are explicitly considered.

## Conclusions and Outlook

This work shows that the environmental behavior and hazard of ZIF-8 cannot be inferred from its pristine properties alone but instead emerge from a sequence of hierarchical transformations. Primary, gas-phase aging in air and reactive gases (O_3_, NO_2_) leaves the Zn–imidazolate framework essentially intact: Zn K-edge XANES/EXAFS spectra of activated and gas-exposed powders are nearly indistinguishable, with only minor changes in second-shell amplitude and no evidence of Zn oxidation or framework collapse. Given that ambient O_3_/NO_2_ levels are typically in the tens to low hundreds of ppb (and standards/guidelines are set at comparable scales), the minimal changes observed here even at ppm-level stress tests support the conclusion that dry atmospheric exposure is unlikely to be the dominant driver of ZIF-8 transformation relative to aqueous/biotic processing. This “priming” tier therefore has limited toxicological relevance on its own but defines the starting state for subsequent interactions. Secondary transformations in aqueous media are much more pronounced. Across natural BHW, ASW, WW, and CCM; XAS, FTIR, and XPS collectively reveal progressive hydrolysis of Zn–N bonds, growth of Zn–O­(H)/Zn–OH features, and the accumulation of organic and inorganic corona species on the particle surface. These changes are matrix-dependent: complex, organic-, and phosphate-rich media promote faster framework degradation and more extensive Zn–O/P coordination than simple electrolyte solutions. Tertiary, organism-level processing in *D. magna* completes the respeciation cascade. Whole-organism micro-XRF shows strong Zn enrichment in the gut and carapace, while micro-XAS spectra from these regions no longer resemble ZIF-8 or aqueous-aged references. Excreted particles, following depuration into fresh BHW, lack Bragg diffraction and display Zn colocalized with P and O in HAADF–STEM-EDS maps, consistent with formation of Zn-rich biominerals and Zn-phosphate-like phases rather than intact MOF.

The inclusion of a 24 day single-generation chronic reproduction experiment allows assessment of sustained life-history impacts and within-generation acclimation/compensatory responses (e.g., shifts in growth–reproduction allocation) across multiple broods. However, multigenerational acclimation or genetic adaptation was not evaluated here. Previous studies demonstrate that exposure history can modulate tolerance in *D. magna* under metal stress, including zinc, and that transgenerational effects can persist beyond the directly exposed generation.^51–53^ A targeted follow-on study could therefore extend the exposure cascade across generations (e.g., F0–F2/F3) and include rechallenge designs to test whether tolerance develops, persists, or trades off against reproduction and growth under environmentally relevant Zn-phase transformation scenarios.^54^

The ecotoxicological outcomes mirror this speciation trajectory. ZIF-8 causes time and concentration-dependent acute immobilization and mortality of neonates at low μg mL^–1^, and a 0.10 μg mL^–1^ chronic exposure (48 h EC_10_) significantly suppresses cumulative brood size and later broods despite minimal adult mortality. These effects likely arise from a combination of transient particulate exposure, released Zn^2+^, and newly formed Zn–O/P phases that differ in bioavailability from either pristine ZIF-8 or dissolved Zn alone. Looking forward, our hierarchical transformation framework highlights several Safe-and-Sustainable-by-Design levers for MOFs, such as stabilizing linkers against hydrolysis, moderating metal release, or engineering benign transformation products. Methodologically, the combination of multitier aging, synchrotron spectroscopy, and life-cycle bioassays provides a transferable template for assessing other advanced materials along realistic environmental trajectories.

## Experimental Section

### Materials

All materials and reagents used for the studies were of analytical grade. The composition of all the media utilized is discussed in Table S1a. BHW was collected from the University of Birmingham borehole, coarse filtered (0.45 μm) to remove debris and stored at 4 °C in the dark until use. All glassware and plasticware used for trace-metal analysis were acid-washed (10% v/v HNO_3_) and rinsed thoroughly with Milli-Q water prior to use. Certified single-element Zn standard solution (1000 μg mL^–1^ in 2% HNO_3_, Sigma-Aldrich) and yttrium standard (1000 μg mL^–1^, Sigma-Aldrich) were used to prepare calibration and internal-standard solutions for Inductively Coupled Plasma-Mass Spectrometry (ICP–MS). This study used *D. magna* (Bham-2 strain), a freshwater noncephalopod invertebrate crustacean, for standard ecotoxicological assays. No human participants, human samples, vertebrate animals, or cephalopods were used. Under the UK Animals (Scientific Procedures) Act 1986, protected animals are defined as living vertebrates other than humans and living cephalopods; therefore, the *D. magna* assays reported here did not require a UK Home Office project license or institutional animal ethics approval/case number. The culture was maintained in-house in BHW under standard culture conditions (20 ± 1 °C; 16 h light/8 h dark photoperiod). Only healthy neonates (<24 h old) from third–fifth brood adults were used for all toxicity tests. All cultures and assays were conducted under University of Birmingham laboratory procedures and followed OECD TG 202 and OECD TG 211-based test principles.

### Synthesis and Characterization of ZIF-8 MOFs

ZIF-8 nanoparticles were synthesized following the established room-temperature precipitation route reported by Cravillon et al., with minor modifications.^22^ Briefly, Zn­(NO_3_)_2_·6H_2_O (0.05 M) and 2-methylimidazole (0.4 M) were prepared in methanol and rapidly mixed under vigorous stirring at room temperature for 1 h. The precipitate was collected, washed three times with methanol, dried (60 °C, vacuum), and activated under vacuum (120 °C, 4–6 h). Full step-by-step synthesis details (including workup) are provided in the Supporting Information (Section S2). Particle morphology and size were assessed by TEM and Scanning Electron Microscopy (SEM); crystallinity and phase purity were confirmed by PXRD; and linker coordination was verified by FTIR. Representative baseline data sets and full measurement parameters are provided in the Supporting Information (Section S1).

### Hierarchical Transformation Studies

Hierarchical transformation experiments were designed to follow a plausible exposure sequence for water-facing MOFs (air → aqueous media → biota), with the rationale for each tier summarized in Box [Boxed-text box1] and the detailed medium compositions provided in Tables S1a,b.

### Primary Transformation (Air and Reactive Gases)

Before exposure to any atmosphere, ZIF-8 samples were thoroughly activated (solvent-removed) under vacuum at 120 °C for 4–6 h and handled under an inert environment. Approximately 100 mg of activated ZIF-8 was placed in a round-bottom flask continuously purged with N_2_ gas. All sample transfers were conducted in a N_2_-filled glovebox or under flowing N_2_ to avoid contact with ambient air or moisture. The ZIF-8 powder was then exposed to controlled low concentrations of reactive gases (oxidants) in order to evaluate its structural and chemical stability under oxidative atmospheric conditions. In separate experiments, NO_2_ and O_3_ gases were introduced into the flask at mixing ratios of ∼5 ppm and ∼10 ppm for fixed exposure durations of 30, 60, or 120 min. These concentrations exceed typical environmental levels but were intentionally chosen to stress-test the framework’s robustness under aggressive conditions. During gas exposure, the MOF powder was spread evenly across the flask’s inner surface to ensure uniform contact with the gases.

After each exposure period, the flask was flushed with N_2_ to remove residual reactive gases, and the sample was immediately sealed in an airtight container under an inert atmosphere. All subsequent handling of the exposed samples was carried out under N_2_. In a glovebox, the gas-exposed ZIF-8 powder was loaded into a 6 mm-diameter Kapton washer (170 μm thickness) and sealed with Kapton tape. The sealed washer was mounted on a holder and enclosed in an aluminized Mylar bag under N_2_ to prevent any ambient air contact during storage, transport, and analysis. Both the gas-exposed samples and an air-exposed control (activated ZIF-8 left in dry air for 7 days) were preserved for comparative analysis. The samples were transported to the synchrotron facility (Diamond Light Source, UK) for zinc K-edge XAS measurements (beamline B18, as detailed in following sections).

### Secondary Transformation (Environmental and Biological Media)

Following the primary (air/gas) exposure, the stability of ZIF-8 was further evaluated in various aqueous media to simulate environmental and biological fluid conditions. For each test, approximately 100 mg of air-exposed ZIF-8 (from the primary stage) was dispersed in 10 mL of the chosen solution (yielding ∼10,000 μg mL^–1^ suspension). The dispersions were gently stirred at room temperature for 7 days to allow any gradual transformations (e.g., hydrolysis or ion exchange) to occur. Because the objective of this tier was to recover sufficient aged material for ex situ structural/speciation characterization (XAS/XPS/TEM) and to sensitively detect early secondary phase restructuring, the multimedia aging experiments were conducted at high solid loading. This loading is therefore analytical/mechanistic and is not intended to represent ambient environmental concentrations. Environmental relevance is addressed through (i) testing across environmentally plausible media chemistries (Table S1a) and (ii) ecotoxicology spanning 0.01–10 μg mL^–1^, including low-dose chronic testing at 0.10 μg mL^–1^. Five different aqueous media were selected to represent a broad range of environmentally and biologically relevant chemistries. One mM NaNO_3_ in deionized water was used as a low-ionic-strength, near-neutral baseline (pH ∼7.0) with no added phosphate. ASW was used as a high-ionic-strength saline medium (pH ∼7.5) dominated by chloride and sulfate, again with no added phosphate. University of Birmingham BHW was used as a realistic hard freshwater matrix (pH ∼7.0–7.5) containing inorganic carbon, low dissolved organic carbon, and trace phosphate. WW was used as a nutrient- and organic-rich medium (pH ∼7.0–7.4) containing phosphate from KH_2_PO_4_ together with bicarbonate and other wastewater-relevant components. CCM was used as a protein-rich biological medium (pH ∼7.2–7.4) containing inorganic phosphate from the DMEM formulation as well as abundant biomolecular components from serum. A detailed chemistry summary of all five aqueous media, including pH, approximate ionic strength, organic matter proxy, phosphate level, and major anions, is provided in Table S1b.

After the 7 day aging period, the ZIF-8 solids were recovered from each suspension by centrifugation (typically 8000 rpm for 10 min). Post aging, total Zn release in each media was measured using ICPMS (Nexion 350, PerkinElmer, USA) in Helium KED mode to avoid potential interferences. The recovered particles were thoroughly rinsed with deionized water to remove any soluble species, residual salts, or medium components adhering to the surface. The washed powders were then dried in air at ambient temperature. These dried, “aged” ZIF-8 samples (one from each medium) were subjected to a comprehensive characterization to identify any chemical or structural transformations resulting from aqueous exposure. FTIR spectroscopy (in ATR—Attenuated Total Reflection mode) was used to detect changes in functional groups, such as the appearance of O–H stretching bands (indicative of Zn–OH or adsorbed water) or changes in the C–N stretching and ring vibration bands of the imidazolate linker. XPS was performed to examine surface elemental composition and bonding states—for example, detecting any formation of Zn–O bonds or reduction in N content on the surface after aging. TEM imaging enabled direct observation of any morphological alterations, such as particle etching, rounding of edges, or aggregation into clusters after exposure to each medium. To quantify the extent of surface restructuring across media, aged ZIF-8 solids recovered from each medium were analyzed by XPS and the atomic percentages of Zn, C, N, and O were determined. We further derived operational indices including O/N, ΔN (N depletion relative to pristine), and ΔO (O enrichment relative to pristine) to enable a quantitative comparison of transformation severity across media (Table S14). These indices are surface-sensitive proxies for conversion away from Zn–imidazolate chemistry toward oxygen-rich environments (e.g., hydroxylated/oxo-bridged or anion-associated Zn pools); XPS is not used here to assign specific bonding environments but provides robust quantitative measures of N loss and oxygenation.

To estimate the aqueous speciation of Zn released from ZIF-8 under our exposure conditions, equilibrium calculations were performed using Visual MINTEQ v3.1. Model inputs and background electrolyte compositions for ASW, 1 mM NaNO_3_, and BHW were taken from Table S2. ZIF-8 was represented by its constituent Zn fraction (25 wt % Zn), derived from the stoichiometric composition of ZIF-8 (C_8_H_12_N_4_Zn). Total ZIF-8 concentrations of 100 and 200 μg mL^–1^ were used as thermodynamic upper-bound scenarios to bracket the experimental exposure range and evaluate limiting speciation behavior. The organic linker fraction (75 wt % of ZIF-8 mass) was entered as a DOC-equivalent term, calculated by mass balance under the conservative assumption of complete ZIF-8 dissolution. Ionic strength was set to “calculated”, allowing Visual MINTEQ to derive ionic strength internally from the defined electrolyte compositions. pH was fixed to the measured values for each medium (ASW pH 7.5; 1 mM NaNO_3_ pH 7.0; BHW pH 7.0–7.5). No secondary Zn solids were predefined, enabling the model to compute saturation indices and evaluate potential precipitation reactions (e.g., Zn­(OH)_2_(s), ZnC*O*_3_(*s*), hopeite (Zn_3_(PO_4_)_2_·4H_2_O)). For BHW simulations, activity guesses were not imposed (activity guess option unchecked) to minimize convergence artifacts in this multicomponent system.

Furthermore, the aqueous-aged samples were prepared for Zn K-edge XAS analysis to probe changes in the local zinc coordination environment. Each sample was loaded into a Kapton washer (similar to the gas-exposed samples) and sealed and then measured in ambient conditions at Diamond Light Source (using transmission or fluorescence XAS as appropriate). The XANES and EXAFS spectra of the aged ZIF-8 were compared against those of the pristine material to detect subtle shifts in Zn’s oxidation state or coordination geometry after different medium treatments. This high-sensitivity XAS approach was used to reveal, for instance, changes in Zn’s local coordination environment or changes in bond distances due to defect formation.^55^

To quantify medium-dependent aqueous transformation, time-resolved dissolved Zn release from ZIF-8 was measured in BHW, ASW, CCM (DMEM + 10% FBS), and 1 mM NaNO_3_ at initial ZIF-8 loadings of 100 and 200 μg mL^–1^. At predefined time points, aliquots were collected and operationally separated into the dissolved fraction using the same dissolved-fraction workflow described above; dissolved Zn concentrations were quantified using ICP–MS. Here, “dissolved Zn” refers to total dissolved Zn in the operationally defined dissolved fraction (not free Zn^2+^). Dispersions were prepared from freshly sonicated ZIF-8 stocks and incubated at 20 ± 1 °C under gentle agitation. These loadings in BHW were selected to provide time-resolved dissolved Zn profiles with strong signal-to-noise while remaining within the concentration regime used for acute hazard testing (0.01–10 μg mL^–1^) and depuration assays. At selected time points (0–336 h), aliquots were withdrawn and separated into dissolved and particulate fractions by centrifugal ultrafiltration using 3.5 kDa Molecular weight cut off (MWCO) filters (Amicon Ultra, Merck, UK) prerinsed with ultrapure water. Filtrates were immediately acidified to 2% (v/v) trace-metal grade HNO_3_ and stored at 4 °C. Dissolved Zn was quantified by ICP–MS (NexION 350D, PerkinElmer, USA) operated in He KED mode with external calibration from a certified Zn standard and yttrium as an internal standard; procedural blanks and matrix-matched QC samples were included to verify data quality.

### Tertiary Transformation (*D. magna*Exposure)

To investigate ZIF-8 transformations under biologically active conditions, acute exposure experiments were performed with the freshwater crustacean *D. magna*. *D. magna* was selected as the tertiary tier because it is the OECD standard freshwater test species (TG 202/211) and, as a filter-feeder, provides a mechanistically relevant model for particle ingestion and gut-mediated transformation in freshwater exposure cascades. *D. magna* neonates (<24 h old, Bham2 laboratory strain) were used as a model organism due to their sensitivity to waterborne contaminants and their widespread use in nanomaterial ecotoxicology. For the exposure, groups of 5 neonates were placed in 20 mL of BHW (the same natural water matrix used in the aging studies) in small glass beakers. The medium for the treatment group was dosed with a suspension of ZIF-8 (which had been preaged in BHW for 7 days as part of the hierarchical study) at a concentration of 10 μg mL^–1^ and 25 μg mL^–1^. A control group (*D. magna* in clean BHW without ZIF-8) was run in parallel. The exposures were carried out for 12 h under well-controlled conditions: the beakers were gently aerated to maintain dissolved oxygen, kept at a constant temperature (20 ± 1 °C), and subjected to a 16:8 h light:dark photoperiod, in line with standard *D. magna* acute toxicity assay protocols. No food was provided during this period to ensure that the daphnids would actively filter-feed on the ZIF-8 particles suspended in the water. Throughout the 12 h exposure, the *D. magna* were observed to swim freely, thereby encountering and ingesting ZIF-8 particles dispersed in the medium.

After 12 h, the organisms were carefully removed from the suspension and briefly rinsed in fresh BHW to dislodge any ZIF-8 particles adhering to their exoskeletons externally. The daphnids were then immediately frozen and lyophilized (freeze-dried). Lyophilization is commonly used in microspectroscopic sample preparation because rapid freezing immobilizes tissues and subsequent water removal helps minimize deformation and reduces liquid-phase diffusion during drying. It helps to preserve spatial patterns relative to air-drying, although redistribution of highly mobile soluble species cannot be fully excluded.^56–58^ The dry, preserved *D. magna* specimens were mounted on Kapton tape for microscopic X-ray analysis. Synchrotron-based XRF mapping and Zn K-edge micro-XAS were employed at the Diamond Light Source I18 beamline (microfocus spectroscopy beamline, experiment number SP40242-1) to examine the biotransformation of ZIF-8 in vivo. Each daphnid was scanned with a focused X-ray beam (∼2 μm spot size, energy tuned around the Zn K-edge at ∼9.65 keV). Two-dimensional XRF maps were first collected by raster-scanning the daphnid, which revealed the spatial distribution of zinc and other relevant elements (e.g., phosphorus, calcium) within the organism. Next, XANES spectra at the Zn K-edge were acquired from selected regions of interest identified in the maps—for example, from the areas with high Zn concentration. By comparing the Zn K-edge XANES from the exposed daphnids to reference spectra (control *D. magna*, pristine ZIF-8, and likely Zn-containing transformation products such as ZnO, ZnSO_4_, ZnNO_3_, ZnS, Zn_3_(PO_4_)_2_, and ZnF_2_) measured at the Diamond B18 beamline (Experiment number SP40080-2 and SP41674-2), we could determine whether the ZIF-8 remained intact inside the organism or had chemically transformed. This approach allowed us to directly probe the speciation of the internalized zinc, providing molecular-level evidence of any biotransformation processes occurring within the 12 h exposure period.

In a further step, a subset of the ZIF-8-exposed *D. magna* was transferred to clean BHW after the 12 h exposure for a depuration phase. The daphnids were kept in particle-free medium for an additional 12 h to allow them to egest (excrete) any ingested ZIF-8 remnants. Fecal pellets and excreta from these depuration tanks were collected, and the particles therein were recovered by gentle centrifugation of the water. The aim was to isolate ZIF-8 particles that had passed through the gut, for ex situ analysis of how the daphnid digestive process altered them. The recovered particles (postdigestion ZIF-8) and, for comparison, pristine ZIF-8 particles were analyzed by TEM to visualize morphological and structural differences. TEM grids were prepared by depositing a drop of a dilute particle suspension onto ultrathin carbon films. Because MOF like ZIF-8 can be highly sensitive to electron beam damage, all electron microscopy was performed under low-dose conditions. Specifically, a low beam current (on the order of 0.05–0.1 nA measured at the sample) was used, achieved by selecting a small condenser aperture (e.g., 10 μm) and a large spot size setting on the microscope. This minimized radiolysis and structural collapse of the MOF during imaging and diffraction. SAED patterns were obtained from both the pristine and the daphnid-processed ZIF-8 particles. To complement the TEM imaging, STEM coupled with EDS was performed on the postdepuration samples to analyze their elemental composition. STEM-EDS mapping (using a Talos F200X G2 TEM at 200 kV) provided spatial distribution of zinc and other elements in the digested particles. A moderate probe current (∼1 nA) and a short pixel dwell time (∼100 μs) were used during mapping to reduce beam exposure per area while still obtaining sufficient X-ray counts for elemental identification.

### Ecotoxicity Testing of ZIF-8

#### Acute Immobilization Assay

The acute toxicity of ZIF-8 to *D. magna* was evaluated using a 48 h immobilization test based on OECD Test Guideline 202, with minor modifications to enhance environmental relevance.^19^ Neonates (<24 h old) from healthy laboratory cultures of the Bham-2 clone were used throughout. Test vessels (acid-washed glass beakers) contained 5 neonates in 10 mL BHW, which was selected as the exposure medium to remain consistent with the aging experiments performed on ZIF-8. A concentration series spanning environmentally realistic to clearly effect-level exposures was prepared freshly in BHW on the day of the test (0, 0.01, 0.05, 0.10, 0.50, 1.0, 5.0, and 10.0 μg mL^–1^ BHW preaged ZIF-8). Each concentration, including the control, was distributed across four replicate vessels (20 daphnids per treatment). Tests were conducted in a temperature-controlled incubator at 20 ± 1 °C, under a 16:8 h light:dark cycle. No food was provided during the 48 h exposure, as specified by the guideline. Immobilization was scored after 24 and 48 h. An individual was considered immobilized if it was unable to swim within 15 s of gentle swirling of the beaker. The control group (BHW only) consistently showed ≤10% immobilization, confirming test validity. Concentration–response data (pooled across replicates) were fitted with a four-parameter logistic function to obtain 24 and 48 h EC_50_ values. The dose–response model used was:

1 $$Y=A_{1}+\frac{A_{2}-A_{1}}{1+10^{\left(\log{\;X}_{0}-X\right)p}}$$

where *A*_1_ and *A*_2_ are the minimum and maximum responses, respectively, *X* is the log_10_ [ZIF-8 concentration], log *X*_0_ is the inflection point (log EC_50_), and *p* is the Hill slope. From the 48 h fit, an EC_10_ was also derived and subsequently used to inform the chronic exposure concentration. As an extension of OECD Test Guideline 202, and reflecting the fact that when testing nanomaterials immobilization as per the OECD test definition does not always equate to mortality as physical adhesion of particles to daphnids may impede swimming without killing the organisms, mortality was also determined on the basis of whether or not the daphnids were able to swim upward in the tubes or not and was recorded alongside OECD immobilization at each ZIF-8 concentration after 24 and 48 h. These data were used to estimate LC_50_ values at both time points by fitting the same four-parameter sigmoidal (logistic) dose–response model described above.

#### Fractionation of Aged Suspensions for Mechanistic Toxicity Testing

ZIF-8 suspensions (100 μg mL^–1^) were aged for 7 days using the same secondary aging protocol as for whole-suspension exposures. The aged suspension was then split into three matched exposure fractions: (i) a particle-free filtrate collected using 3 kDa ultracentrifugation tubes, (ii) washed/resuspended aged particles/precipitates obtained by centrifugation and washing the solids three times prior to resuspension, and (iii) the whole aged suspension (unfractionated). Nominal exposure concentrations (μg mL^–1^) are defined with respect to the parent whole aged suspension from which these fractions were derived. For comparability, equal exposure volumes of filtrate and washed/resuspended particle fractions were used relative to the whole aged suspension at each nominal concentration. Acute immobilization assays were performed for all three fractions under identical conditions (OECD TG 202 style; 48 h end point).

#### Chronic Reproduction Assay

Long-term, sublethal effects of ZIF-8 were assessed using a 24 day chronic exposure (OECD TG 211-based, extended from the standard 21 days to capture delayed broods due to ZIF-8 exposure).^18^ The main deviation from the guideline was the use of natural BHW rather than synthetic hard water, to maintain continuity with the aging and acute toxicity experiments. Neonates (<24 h old) were individually transferred into 100 mL glass beakers containing 50 mL of either control medium (BHW only) or BHW amended with 0.10 μg mL^–1^ ZIF-8 that had been pre-exposed for 7 days to BHW, corresponding to the 48 h EC_10_ from the acute assay. A 100 μg mL^–1^ stock suspension of BHW preaged ZIF-8 was prepared, from which all exposures at 0.10 μg mL^–1^ were freshly diluted to ensure that the particles added were always the same age as those being replaced when the daphnid medium is renewed. Each treatment comprised four replicate beakers per experiment (one daphnid per beaker). Daphnids were maintained at 20 ± 1 °C under a 16 h light/8 h dark cycle. Organisms were fed every second day with a standard freshwater algal suspension at approximately 0.1–0.2 mg C daphnid^–1^ per feed. Medium was renewed every 48 h, immediately after feeding, to sustain ZIF-8 exposure and water quality. During renewal, adults were gently transferred to fresh medium with wide-bore glass pipettes to minimize mechanical stress. At each renewal, all live offsprings were counted and removed. End points assessed included adult survival, cumulative number of neonates per female, and time to first brood. Brood size was recorded separately for each brood (B1–B5), enabling assessment of whether reproductive effects intensified as exposure progressed. At the end of the test, adults (females) from both control and test samples were observed under a stereomicroscope for body length and visible anatomical abnormalities, and representative individuals from control and ZIF-8 treatments were imaged. Reproductive data were analyzed using OriginPro. Differences in cumulative brood size between control and exposed groups at each time point were evaluated with two-sample *t* tests; *p* <0.05 was taken as statistically significant. After 24 day chronic exposure (OECD TG 211-based, extended to capture delayed broods), each female individual from both control and test samples was imaged using an optical microscope to visualize their anatomy.

#### Uptake-Depuration Assay for ZIF-8

Short-term uptake and depuration of ZIF-8 by *D. magna* neonates were studied in a separate 12 h assay. Neonates (<24 h old, Bham-2 clone) were exposed for 12 h to 5.0 μg mL^–1^ ZIF-8 (preaged in BHW for 7 days) in BHW under static, nonfeeding conditions. Experiments were conducted in triplicate using acid-cleaned glass vials, each containing 10 neonates in 5 mL of the ZIF-8 suspension. After 12 h, organisms were gently rinsed three times in fresh BHW to remove loosely attached particles. For depuration, the rinsed neonates were transferred into ZIF-8-free BHW and maintained for a further 12 h under identical temperature and light conditions, again without feeding. At the end of the accumulation and depuration phases, neonates were collected, briefly blotted to remove excess water, and frozen at −80 °C until digestion. Exposure and depuration media were retained for chemical analysis and stored at 4 °C.

TEM was carried out on a JEOL JEM 2100 operated at 200 kV. Pristine ZIF-8, as well as particulate materials recovered from the depuration medium, was drop-cast onto ultrathin continuous carbon films on 200-mesh gold grids and allowed to air-dry. Owing to the beam sensitivity of ZIF-8, SAED was acquired under low-dose conditions, using the 50 μm condenser aperture and a high numerical number of spot size (i.e., spot size 3), yielding on-sample electron fluence of 14 pA/cm^2^. For compositional mapping, it was done on the Talos F200X G2 TEM operated at 200 kV. The microscope was operated under STEM mode with HAADF imaging coupled to EDS to map Zn and major light elements (C, O, P, S, Ca, Mg, Cl). STEM–EDS maps were collected with a probe current of ∼0.47 nA, a convergence semiangle of 10 mrad, and a dwell time of 50 μs pixel^–1^. For ICP–MS analysis of Zn, exposure and depuration media were mixed thoroughly by pipetting immediately prior to subsampling. Aliquots (2 mL) were combined with ashing mixture (H_2_O_2_ and HNO_3_) and digested using a two-step protocol adapted from previous work on engineered nanomaterials: samples were heated for 20 min at boiling temperature to promote complete breakdown of ZIF-8 while limiting thermal degradation of dissolved organics and then passed through 0.22 μm PTFE syringe filters to remove residual particulates and debris. Zinc concentrations were quantified by ICP–MS (NexION 350, PerkinElmer, USA) operated in kinetic energy discrimination mode with He as the collision gas to minimize polyatomic interferences. Calibration standards were prepared from a certified Zn stock solution (1000 μg mL^–1^) diluted in deionized water. Yttrium was used as an internal standard to correct for matrix effects and instrumental drift. Procedural blanks and quality-control solutions were analyzed every 10 samples to verify stability of the instrument and reproducibility of the digestion procedure. Total Zn per neonate was calculated from the measured concentrations, allowing estimation of ZIF-8 uptake after 24 h exposure and the fraction eliminated during depuration.

All ecotoxicity data are presented as mean ± SD. Statistical comparisons shown in Figure 6 were performed using unpaired two-tailed Student’s *t* tests: replicate beakers versus control for the acute 48 h data sets (Figure 6a,b), day 24 cumulative brood size and brood-wise comparisons for the chronic assay (Figure 6c,d), and control, retained, and depurated fractions for the uptake–depuration assay (Figure 6f). A *p* value <0.05 was considered statistically significant.

## Supplementary Material



## Notes

Additional experimental details for ZIF-8 synthesis, hierarchical aging, Zn K-edge XAS, XPS, TEM/STEM–EDS, visual MINTEQ modeling, ICP–MS analysis, *D. magna* acute/chronic ecotoxicity, uptake–depuration assays, supplementary figures, and supplementary tables (PDF)

## References

[1] Singh S., Sivaram N., Nath B., Khan N. A., Singh J., Ramamurthy P. C. (2024). Metal Organic
Frameworks for Wastewater Treatment,
Renewable Energy and Circular Economy Contributions. npj Clean Water.

[2] Burtch N. C., Jasuja H., Walton K. S. (2014). Water Stability
and Adsorption in
Metal–Organic Frameworks. Chem. Rev..

[3] Rieth A. J., Wright A. M., Dincă M. (2019). Kinetic Stability
of Metal–Organic
Frameworks for Corrosive and Coordinating Gas Capture. Nat. Rev. Mater..

[4] Bužek D., Demel J., Lang K. (2018). Zirconium Metal-Organic
Framework
UiO-66: Stability in an Aqueous Environment and Its Relevance for
Organophosphate Degradation. Inorg. Chem..

[5] Kandiah M., Nilsen M. H., Usseglio S., Jakobsen S., Olsbye U., Tilset M., Larabi C., Quadrelli E. A., Bonino F., Lillerud K. P. (2010). Synthesis and Stability
of Tagged
UiO-66 Zr-MOFs. Chem. Mater..

[6] Chakraborty S., Dhumal P., Mikulska I., Pham S., Ellis Bradford L. J., Menon D., Misra S. K., Lynch I. (2025). Biotic Transformation
of Abiotically Stable Nanoscale UiO-66 Metal–Organic Framework
by Daphnia Magna Results in Chronic Reproductive Toxicity. ACS Nano.

[7] Zhang H., Zhao M., Lin Y. S. (2019). Stability
of ZIF-8 in Water under
Ambient Conditions. Microporous Mesoporous Mater..

[8] Velásquez-Hernández M. D. J., Ricco R., Carraro F., Limpoco F. T., Linares-Moreau M., Leitner E., Wiltsche H., Rattenberger J., Schröttner H., Frühwirt P., Stadler E. M., Gescheidt G., Amenitsch H., Doonan C. J., Falcaro P. (2019). Degradation of ZIF-8
in Phosphate Buffered Saline Media. CrystEngComm.

[9] Luzuriaga M. A., Benjamin C. E., Gaertner M. W., Lee H., Herbert F. C., Mallick S., Gassensmith J. J. (2019). ZIF-8 Degrades
in Cell Media, Serum,
and Some—But Not All—Common Laboratory Buffers. Supramol. Chem..

[10] Qiu X., Liu L., Xu W., Chen C., Li M., Shi Y., Wu X., Chen K., Wang C. (2022). Zeolitic Imidazolate Framework-8
Nanoparticles Exhibit More Severe Toxicity to the Embryo/Larvae of
Zebrafish (Danio Rerio) When Co-Exposed with Cetylpyridinium Chloride. Antioxidants.

[11] Menon D., Chakraborty S. (2023). How Safe Are
Nanoscale Metal-Organic Frameworks?. Front.
Toxicol..

[12] Goyal P., Soppina P., Misra S. K., Valsami-Jones E., Soppina V., Chakraborty S. (2022). Toxicological
Impact and in Vivo
Tracing of Rhodamine Functionalised ZIF-8 Nanoparticles. Front. Toxicol..

[13] Zhang X., Hu X., Wu H., Mu L. (2021). Persistence and Recovery of ZIF-8
and ZIF-67 Phytotoxicity. Environ. Sci. Technol..

[14] Jiang J. Q., Yang C. X., Yan X. P. (2013). Zeolitic
Imidazolate Framework-8
for Fast Adsorption and Removal of Benzotriazoles from Aqueous Solution. ACS Appl. Mater. Interfaces.

[15] Gottschalk F., Sonderer T., Scholz R. W., Nowack B. (2009). Modeled Environmental
Concentrations of Engineered Nanomaterials (TiO2, ZnO, Ag, CNT, Fullerenes)
for Different Regions. Environ. Sci. Technol..

[16] Gottschalk F., Sun T., Nowack B. (2013). Environmental
Concentrations of Engineered Nanomaterials:
Review of Modeling and Analytical Studies. Environ.
Pollut..

[17] Sun T. Y., Conroy G., Donner E., Hungerbühler K., Lombi E., Nowack B. (2015). Probabilistic Modelling
of Engineered
Nanomaterial Emissions to the Environment: A Spatio-Temporal Approach. Environ. Sci.:Nano.

[18] Test No. 211: Daphnia Magna Reproduction Test. 2012. 10.1787/9789264185203-EN.

[19] Test No. 202: Daphnia Sp. Acute Immobilisation Test. 2004. 10.1787/9789264069947-EN.

[20] Reilly K., Ellis L. J. A., Davoudi H. H., Supian S., Maia M. T., Silva G. H., Guo Z., Martinez D. S. T., Lynch I. (2023). Daphnia as
a Model Organism to Probe Biological Responses to Nanomaterials—from
Individual to Population Effects via Adverse Outcome Pathways. Front. Toxicol..

[21] Zhu X., Wang J., Zhang X., Chang Y., Chen Y. (2010). Trophic Transfer
of TiO2 Nanoparticles from Daphnia to Zebrafish in a Simplified Freshwater
Food Chain. Chemosphere.

[22] Cravillon J., Münzer S., Lohmeier S. J., Feldhoff A., Huber K., Wiebcke M. (2009). Rapid Room-Temperature
Synthesis and Characterization
of Nanocrystals of a Prototypical Zeolitic Imidazolate Framework. Chem. Mater..

[23] Phan A., Doonan C. J., Uribe-Romo F. J., Knobler C. B., Okeeffe M., Yaghi O. M. (2009). Synthesis, Structure, and Carbon Dioxide Capture Properties
of Zeolitic Imidazolate Frameworks. Acc. Chem.
Res..

[24] Park K. S., Ni Z., Côté A. P., Choi J. Y., Huang R., Uribe-Romo F. J., Chae H. K., O’Keeffe M., Yaghi O. M. (2006). Exceptional Chemical
and Thermal Stability of Zeolitic
Imidazolate Frameworks. Proc. Natl. Acad. Sci.
U.S.A..

[25] Papurello R. L., Lozano L. A., Ramos-Fernández E. V., Fernández J. L., Zamaro J. M. (2019). Post-Synthetic Modification of ZIF-8 Crystals and Films
through UV Light Photoirradiation: Impact on the Physicochemical Behavior
of the MOF. ChemPhysChem.

[26] Taheri M., Tsuzuki T. (2021). Photo-Accelerated Hydrolysis of Metal
Organic Framework
ZIF-8. ACS Mater. Lett..

[27] Zhang H., Zhao M., Yang Y., Lin Y. S. (2019). Hydrolysis and Condensation
of ZIF-8 in Water. Microporous Mesoporous Mater..

[28] Wang H., Jian M., Qi Z., Li Y., Liu R., Qu J., Zhang X. (2018). Specific Anion Effects
on the Stability of Zeolitic
Imidazolate Framework-8 in Aqueous Solution. Microporous Mesoporous Mater..

[29] Tanaka S., Tanaka Y. (2019). A Simple Step toward
Enhancing Hydrothermal Stability
of ZIF-8. ACS Omega.

[30] Butonova S. A., Ikonnikova E. V., Sharsheeva A., Chernyshov I. Y., Kuchur O. A., Mukhin I. S., Hey-Hawkins E., Vinogradov A. V., Morozov M. I. (2021). Degradation Kinetic Study of ZIF-8
Microcrystals with and without the Presence of Lactic Acid. RSC Adv..

[31] Chakraborty S., Mikulska I., Dhumal P., Langford N., Nehzati S., Boseley R., Pham S., Pfrang C., Kaur M., Valsami-Jones E., Ignatyev K., Menon D., Misra S. K., Lynch I. (2026). Mapping the
Hierarchical Environmental Transformations of Nanoscale
UiO-66 Metal–Organic Framework. Environ.
Sci. Technol..

[32] Bůžek D., Adamec S., Lang K., Demel J. (2021). Metal–Organic
Frameworks vs. Buffers: Case Study of UiO-66 Stability. Inorg. Chem. Front..

[33] Zhang Y., Jia Y., Li M., Hou L. (2018). Influence of the 2-Methylimidazole/Zinc
Nitrate Hexahydrate Molar Ratio on the Synthesis of Zeolitic Imidazolate
Framework-8 Crystals at Room Temperature. Sci.
Rep..

[34] Wu R., Hong B., Xue C., Chen Z., Chen Z. (2024). ZIF-8 Used
for the Selective Recovery of Heavy Rare Earth Elements from Mining
Wastewater. Environ. Sci. Technol..

[35] Frankcombe T. J., Liu Y. (2023). Interpretation of Oxygen
1s X-Ray Photoelectron Spectroscopy of ZnO. Chem. Mater..

[36] Zhou L., Li N., Owens G., Chen Z. (2019). Simultaneous Removal of Mixed Contaminants,
Copper and Norfloxacin, from Aqueous Solution by ZIF-8. Chem. Eng. J..

[37] Soliman A. I. A., Abdel-Wahab A.-M. A., Abdelhamid H. N. (2022). Hierarchical
porous zeolitic imidazolate frameworks (ZIF-8) and ZnO@N-doped carbon
for selective adsorption and photocatalytic degradation of organic. RSC Adv..

[38] Chakraborty S., Misra S. K. (2019). A Comparative Analysis
of Dialysis Based Separation
Methods for Assessing Copper Oxide Nanoparticle Solubility. Environ. Nanotechnol. Monit. Manag..

[39] Cheng M. L.-H., Tsui M. T. K., Monteverde M. R., Kwon S. Y., Hoang T. C. (2025). Trophic
Transfer of Trace Elements within and between Aquatic and Terrestrial
Food Webs in a Forested Watershed. Front. Environ.
Sci..

[40] Lopes S., Ribeiro F., Wojnarowicz J., Lojkowski W., Jurkschat K., Crossley A., Soares A. M. V. M., Loureiro S. (2013). Zinc Oxide Nanoparticles Toxicity to Daphnia Magna:
Size-dependent Effects and Dissolution. Environ.
Toxicol. Chem..

[41] Santos-Rasera J. R., Monteiro R. T. R., de Carvalho H. W. P. (2022). Investigation of Acute Toxicity,
Accumulation, and Depuration of ZnO Nanoparticles in Daphnia Magna. Sci. Total Environ..

[42] Lovern S. B., Strickler J. R., Klaper R. (2007). Behavioral and Physiological Changes
in Daphnia Magna When Exposed to Nanoparticle Suspensions (Titanium
Dioxide, Nano-C60, and C60HxC70Hx). Environ.
Sci. Technol..

[43] Herrmann R., García-García F. J., Reller A. (2014). Rapid Degradation of
Zinc Oxide Nanoparticles by Phosphate Ions. Beilstein J. Nanotechnol..

[44] Reed R. B., Ladner D. A., Higgins C. P., Westerhoff P., Ranville J. F. (2012). Solubility of Nano-Zinc Oxide in Environmentally and
Biologically Important Matrices. Environ. Toxicol.
Chem..

[45] Chakraborty S., Lynch I. (2025). Biomolecular Transformations
Shape the Environmental Fate of Nanoscale
and Emerging Materials. Acc. Chem. Res..

[46] Abdolahpur
Monikh F., Chupani L., Karkossa I., Gardian Z., Arenas-Lago D., von Bergen M., Schubert K., Piackova V., Zuskova E., Jiskoot W., Vijver M. G., Peijnenburg W. J. G. M. (2021). An
Environmental Ecocorona Influences the Formation and Evolution of
the Biological Corona on the Surface of Single-Walled Carbon Nanotubes. NanoImpact.

[47] Wheeler K. E., Chetwynd A. J., Fahy K. M., Hong B. S., Tochihuitl J. A., Foster L. A., Lynch I. (2021). Environmental
Dimensions of the Protein
Corona. Nat. Nanotechnol..

[48] Tangaa S. R., Selck H., Winther-Nielsen M., Khan F. R. (2016). Trophic Transfer
of Metal-Based Nanoparticles in Aquatic Environments: A Review and
Recommendations for Future Research Focus. Environ.
Sci.:Nano.

[49] Skjolding L. M., Winther-Nielsen M., Baun A. (2014). Trophic Transfer of Differently Functionalized
Zinc Oxide Nanoparticles from Crustaceans (Daphnia Magna) to Zebrafish
(Danio Rerio). Aquat. Toxicol..

[50] Liu X., Wang X., Kapteijn F. (2020). Water and
Metal–Organic Frameworks:
From Interaction toward Utilization. Chem. Rev..

[51] Bossuyt B. T. A., Janssen C. R. (2003). Acclimation of Daphnia
Magna to Environmentally Realistic
Copper Concentrations. Comp. Biochem. Physiol.,
Part C:Toxicol. Pharmacol..

[52] Jeremias G., Muñiz-González A. B., Mendes Gonçalves F. J., Martínez-Guitarte J. L., Asselman J., Luísa
Pereira J. (2024). History of Exposure to Copper Influences Transgenerational
Gene Expression Responses in Daphnia Magna. Epigenetics.

[53] Tsui M. T. K., Wang W. X. (2007). Biokinetics and
Tolerance Development of Toxic Metals
in Daphnia Magna. Environ. Toxicol. Chem..

[54] Chakraborty S. (2025). Environmental
Hierarchy as the Third Dimension of Nanomaterial Transformation Science. Eco-Environ. & Health.

[55] Chakraborty S., Britto S., Gomez-Gonzalez M., Buzanich A. G., Mikulska I. (2025). Synchrotrons,
Neutron Sources, and XFELs Guiding the Future of Safe and Sustainable
Nanomaterials. Cell Rep. Phys. Sci..

[56] Molnar A., Lakat T., Hosszu A., Szebeni B., Balogh A., Orfi L., Szabo A. J., Fekete A., Hodrea J. (2021). Lyophilization
and Homogenization of Biological Samples Improves Reproducibility
and Reduces Standard Deviation in Molecular Biology Techniques. Amino Acids.

[57] Pushie M. J., Pickering I. J., Korbas M., Hackett M. J., George G. N. (2014). Elemental
and Chemically Specific X-Ray Fluorescence Imaging of Biological Systems. Chem. Rev..

[58] De
Jonge M. D., Vogt S. (2010). Hard X-Ray Fluorescence Tomography
— an Emerging Tool for Structural Visualization. Curr. Opin. Struct. Biol..

