# Supplementary material for: Hierarchical Environmental Exposure Transforms Zeolitic Imidazolate Framework‑8 and Increases Toxicity in Daphnia magna
Source: ACS Nano. 2026 May 26;20(22):16096–117. doi: 10.1021/acsnano.6c01107 (PMC13255527; doi:10.1021/acsnano.6c01107)
Supplement: Supplementary file 1 [file nn6c01107_si_001.pdf]

Supplementary Information for

Hierarchical environmental exposure transforms zeolitic imidazolate framework-8 and increases toxicity in *Daphnia magna*

Swaroop Chakraborty*[a], Iuliia Mikulska[b], Rhiannon Boseley[b], Sang Pham[c], Prathmesh Bhadane[d], Pankti Dhumal[a], Santanu Majumder[e], Jajati Mandal[f], Tina Geraki[b], Superb K. Misra[d], Christian Pfrang[a], Iseult Lynch[a]

[a] School of Geography, Earth & Environmental Sciences, University of Birmingham, Edgbaston, B15 2TT, UK

[b] Diamond Light Source, Harwell Science and Innovation Campus, Didcot, OX11 0DE UK.

[c] Facility of Electron Microscopy, University of Birmingham, Edgbaston, B15 2TT, UK

[d] Materials Engineering, Indian Institute of Technology, Gandhinagar, 382355, India.

[e]School of Life and Environmental Sciences, Bournemouth University (Talbot Campus), Fern Barrow, Poole BH125BB, UK

[f]School of Science, Engineering and Environment, University of Salford, Salford M5 4WT, United Kingdom

*s.chakraborty@bham.ac.uk, i.lynch@bham.ac.uk

S1. Synthesis and Physicochemical characterization of nanoscale ZIF-8 MOFs.

ZIF-8 (Zn(2-methylimidazole) $_2$) MOF nanoparticles were synthesized following the room-temperature precipitation method of Cravillon *et al.* (2009), with slight modifications. Zinc nitrate hexahydrate [Zn $(NO_3)_2 \cdot 6H_2O$] was dissolved in methanol (0.05 M, 100 mL), and 2-methylimidazole (mIm) was dissolved in a separate methanol volume (0.4 M, 100 mL). Each solution was stirred until all solids fully dissolved, forming clear precursor solutions. The Zn $(NO_3)_2$ solution was dropwise poured into the mIm solution under vigorous stirring at room temperature. This rapid mixing ensured supersaturation and instant nucleation of ZIF-8 nanocrystals. The combined mixture was continuously stirred for 1 hour at ambient conditions. Within minutes, the solution turned turbid, indicating the formation of ZIF-8 particles as a milky-white suspension. Prolonged stirring for 60 minutes allowed the nanocrystals to grow to the desired size distribution. The ZIF-8 suspension was collected and centrifuged (or allowed to settle) to separate the white precipitate from the supernatant. The solid product was washed three times with fresh methanol to remove unreacted ligands or salts, each time re-dispersing the solid and centrifuging again. The washed ZIF-8 precipitate was dried in a vacuum oven at 60 °C for 4 hours. This gentle drying removed residual solvent without decomposing the framework. The resulting dry powder (yield: a few

grams) was a fluffy white material. To ensure open porosity by removing any occluded guest molecules, the dry ZIF-8 powder was activated under vacuum at 120 °C for 4–6 hours. This activation step evacuates residual methanol from the pores, yielding fully activated ZIF-8.

The pristine activated ZIF-8 nanoparticles were characterized using a suite of analytical techniques to verify their structure, composition, and surface properties. Transmission Electron Microscopy (TEM, Jeol 2100, Japan) and Scanning Electron Microscopy (JEOL JSM-7900F) were used to examine the particle size, morphology, and crystallinity of ZIF-8. Aliquots of the nanoparticle suspension were deposited on carbon-coated grids and silicon wafers for TEM and SEM, respectively. Fourier Transform Infrared Spectroscopy (FTIR, Perkin Elmer Spectrum, USA) spectra were recorded (600–4000 cm^{-1}) to identify characteristic functional groups and confirm the framework's composition. Synchrotron based X-ray Absorption Spectroscopy (XAS) at the Zn K-edge (≈9659 eV) was employed to probe the local zinc coordination environment in ZIF-8, especially after various exposures (see below for details). X-ray Absorption Near Edge Structure (XANES) provided information on Zn oxidation state and bonding, while Extended X-ray Absorption Fine Structure (EXAFS) analysis gave qualitative insight into the Zn–N/C coordination number and bond distances. The crystallinity and phase purity of synthesized ZIF-8 was confirmed by Powder X-ray Diffraction (PXRD, Malvern Panalytical, UK).

S2. Baseline characterization of nanoscale ZIF-8 MOFs.

TEM and SEM **(Figures S1a, b)** confirm that the pristine ZIF-8 is obtained as well-defined nanoparticles. TEM reveals loosely agglomerated faceted crystallites with dimensions on the order of a few tens of nanometres, typical of nanoscale ZIF-8 prepared by room-temperature routes[1]. SEM provides a clearer view of individual particles: using ImageJ software (using the 100 nm scale bar for calibration) yields an average particle diameter of 47 ± 4 nm (mean ± standard deviation). The size distribution is relatively narrow, with most particles falling in the ~44–54 nm range and only a small fraction of larger aggregates. This size regime is consistent with previously reported nano-ZIF-8 syntheses, which typically produce particles between ~40 and 200 nm depending on modulator content and synthesis conditions.[1,2] The combination of sub-100-nm size and high colloidal density is particularly relevant for *D. magna* exposure, as these particles are readily ingestible and can interact efficiently with the filtering apparatus. The PXRD pattern of the as-synthesised material **(Figure S1c)** matches well with the simulated and experimental patterns of sodalite-type ZIF-8 reported in the literature.[1,2] Sharp reflections at low angles (around 7.3°, 10.4°, 12.7°, 14.7° and 16.4° 2θ) and the absence of additional peaks attributable to ZnO, $Zn(OH)_2$ or other crystalline by-products indicate that the sample is phase-pure ZIF-8 with good long-range order. The retention of this diffraction pattern after drying confirms that the synthesis and work-up do not induce observable framework collapse.

FT-IR spectroscopy **(Figure S1d)** further substantiates successful formation of the ZIF-8 framework. The spectrum shows the expected aromatic and aliphatic C–H stretching vibrations of the mIm linker in the 3180–2900 cm^{-1} region, together with intense C=N and C–N stretching and ring-breathing bands between ~1600 and 1000 cm^{-1}, in excellent agreement with previous reports on ZIF-8 vibrational spectra[3]. No prominent peaks corresponding to free 2-methylimidazole or nitrate are detected,

suggesting efficient coordination of the linker to Zn^{2+} and effective removal of unreacted precursors during washing. TEM/SEM, PXRD and FT-IR data demonstrate that the starting material used in the ageing, transformation and ecotoxicity experiments is a nanoscale, phase-pure ZIF-8 with well-defined morphology and crystallinity. This provides a robust baseline for interpreting subsequent changes in structure, coordination environment and biological response as genuine transformations rather than artefacts of synthesis.

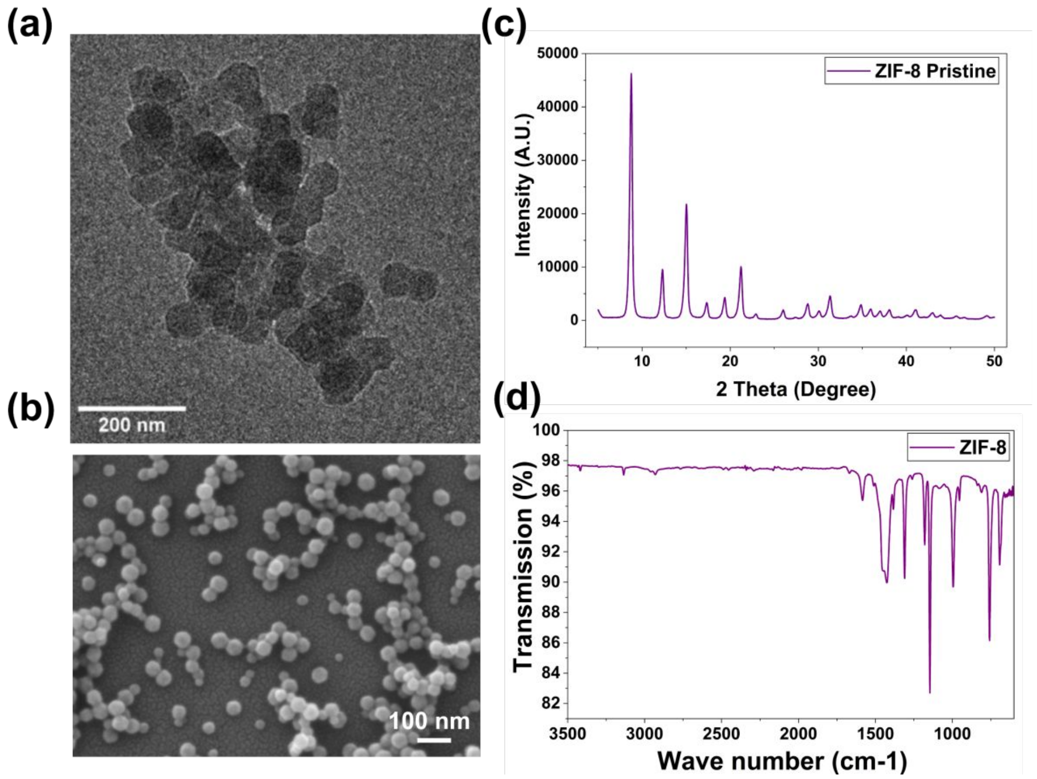

Figure S1. Morphology and structural characterisation of pristine ZIF-8 (a) Bright-field TEM image of pristine ZIF-8 showing aggregates of nano-crystals (scale bar: 200 nm).(b) SEM image of dried ZIF-8 powder displaying a dense population of nearly spherical nanoparticles (scale bar: 100 nm). (c) PXRD pattern of pristine ZIF-8 collected over 5–50° 2θ, showing the reflections characteristic of the sodalite-type ZIF-8 framework and confirming phase purity. (d) FT-IR spectrum of pristine ZIF-8, with bands assigned to the coordinated 2-methylimidazolate linker (C–H stretching and C=N/C–N stretching and ring vibrations), consistent with successful framework formation.

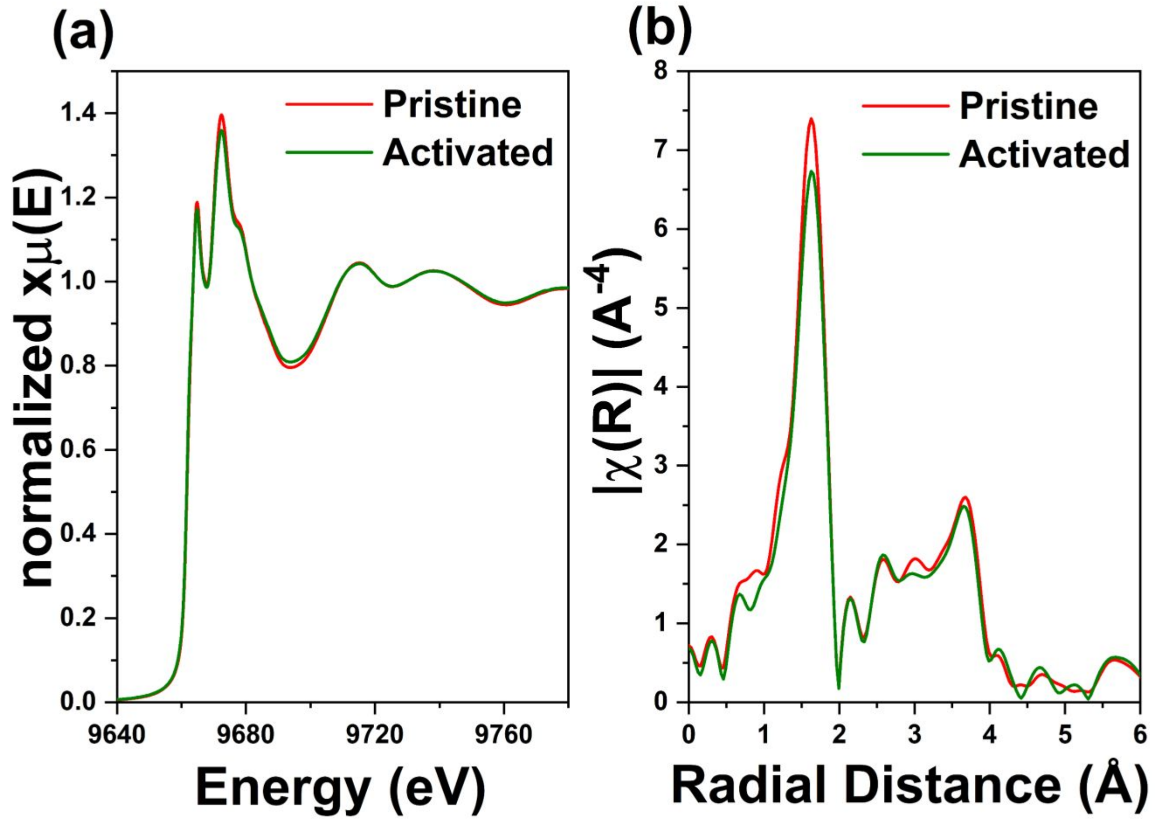

Figure S2. Effect of activation on the local Zn environment in ZIF-8. (a) Normalised Zn K-edge XANES spectra of pristine (red) and activated (green) ZIF-8. The nearly identical edge position and spectral shape indicate that vacuum activation does not change the Zn (II) oxidation state or overall Zn–N coordination geometry. (b) Corresponding Fourier-transformed EXAFS (|χ(R)|, phase-uncorrected) showing a dominant first-shell peak at ~2 Å and similar higher-shell features for both samples; the slightly reduced amplitude for the activated material is consistent with minor increases in static disorder and removal of guest molecules rather than framework degradation.

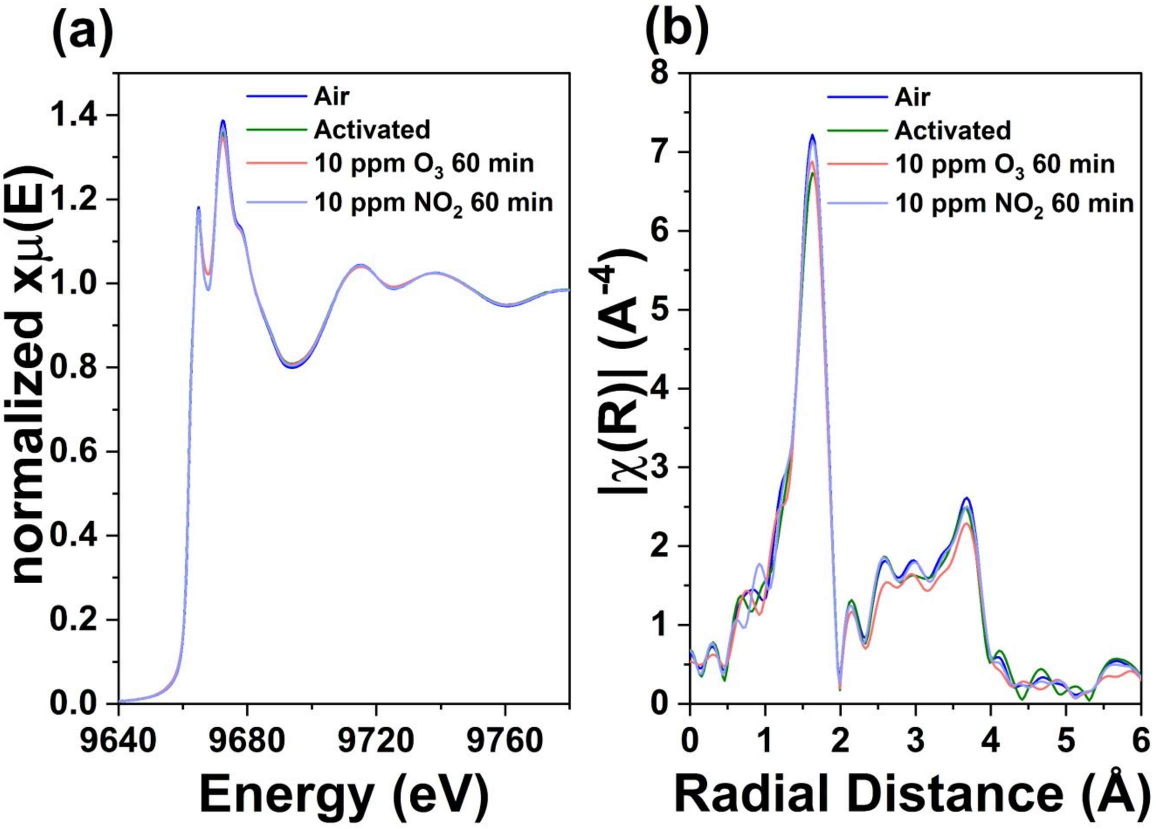

Figure S3. Comparison of air- and gas-exposed ZIF-8 at 10 ppm O_3/NO_2. (a) Normalised Zn K-edge XANES spectra of ZIF-8 stored in air (blue), vacuum-activated (green), and exposed for 60 min to 10 ppm O_3 (red) or 10 ppm NO_2 (light blue). All spectra exhibit closely overlapping edge positions and overall line shapes, indicating retention of the ZIF-8-like Zn(II) coordination environment under these gas-phase conditions. (b) Corresponding |χ(R)| spectra showing a strong first-shell peak and similar medium-range features for all samples; only subtle changes in amplitude and fine structure are observed after O_3 or NO_2 exposure, consistent with surface-limited perturbations of the Zn coordination sphere without loss of framework integrity.

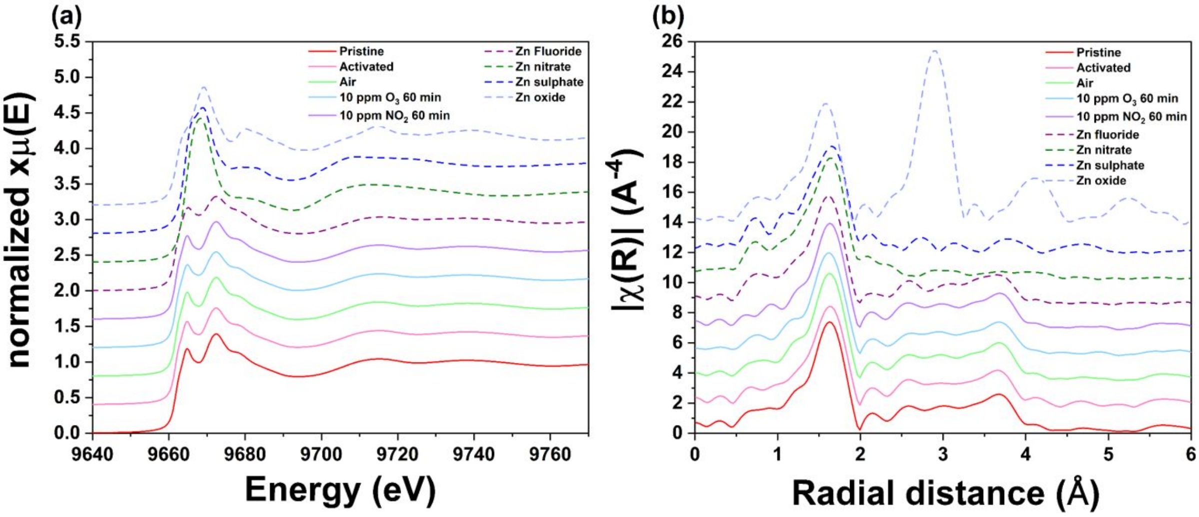

Figure S4. XAS comparison of ZIF-8 with Zn reference compounds and gas-aged samples. (a) Normalised Zn K-edge XANES spectra of pristine ZIF-8, Zn nitrate, Zn oxide, Zn sulphate, Zn fluoride, activated ZIF-8, air-exposed ZIF-8, and ZIF-8 exposed to 10 ppm NO_2 or O_3 for 60 min. The ZIF-8-derived spectra (pristine, activated, air, gas-aged) form a distinct family that is clearly different from those of simple Zn salts and ZnO, confirming that gas exposure does not convert ZIF-8 into these phases. (b) Corresponding |χ(R)| spectra (phase-uncorrected). Reference compounds show characteristic first-shell distances and higher-shell patterns for Zn–O/F environments, whereas all ZIF-8-based samples retain the same ZIF-8-like EXAFS fingerprint with a dominant peak at ~2 Å and similar higher-shell structure, demonstrating preservation of the Zn–imidazolate framework after activation, air storage and short-term exposure to 10 ppm O_3 or NO_2.

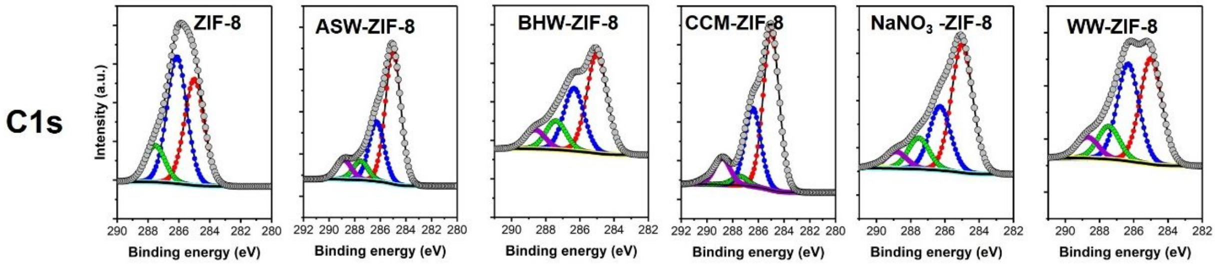

Figure S5. **High-resolution C 1s XPS spectra of pristine and aqueous-aged ZIF-8.** Deconvoluted C 1s envelopes for pristine ZIF-8 and samples aged 7 d in artificial seawater (ASW-ZIF-8), borehole water (BHW-ZIF-8), cell-culture medium (CCM-ZIF-8), 1 mM $NaNO_3$ ($NaNO_3$-ZIF-8) and simulated wastewater (WW-ZIF-8). For all samples, the main component at ~284.7–285.0 eV (blue) is assigned to C–C/C=C/C–N from the 2-methylimidazolate linker and adventitious carbon; higher-binding-energy components at ~286.0–286.5 eV (green) and ~287.5–288.8 eV (red) correspond to C–O/C–N and carbonyl/Carboxyl/O–C=O species, respectively, while a minor low-BE contribution at ~283.5–284.0 eV (yellow) is attributed to more reduced carbon environments. Ageing in natural and complex media leads to an overall increase in the oxidised C–O/C=O components, particularly in CCM-, and WW-ZIF-8, consistent with adsorption of carbonate and organic molecules and the build-up of an eco/bio-corona on the ZIF-8 surface.

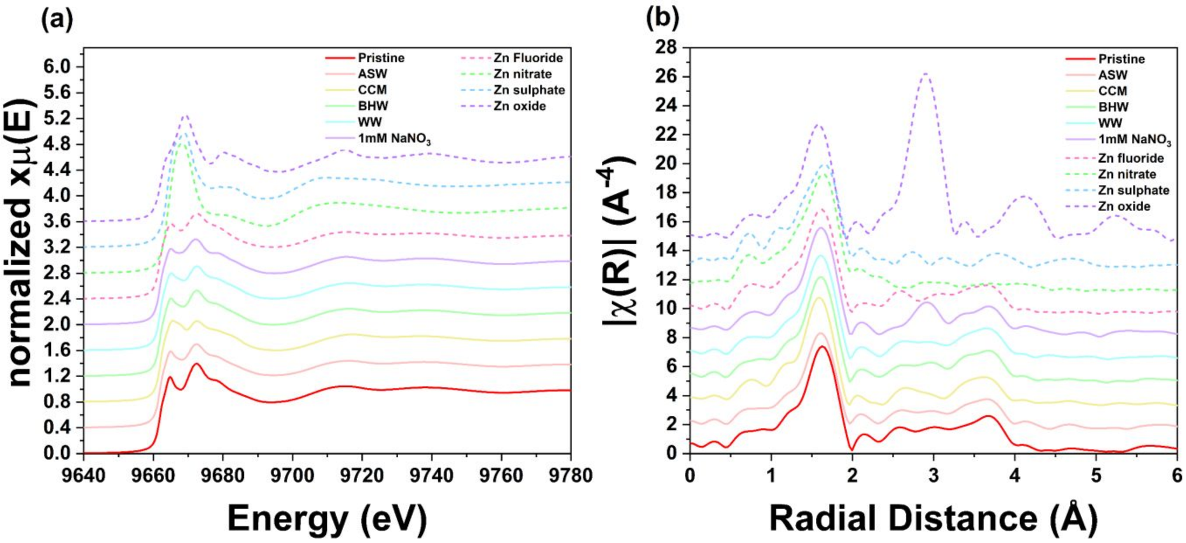

Figure S6. Comparison of Zn K-edge XAS for air aged ZIF-8 aged in aqueous media with dissolved Zn reference compounds. (a) Normalised Zn K-edge XANES spectra of pristine ZIF-8, ZIF-8 aged for 7 d in ASW, CCM, BHW, WW and 1 mM $NaNO_3$, overlaid with aqueous Zn reference spectra for $Zn(NO_3)_2$, ZnO, $ZnSO_4$ and ZnF_2. (b) Corresponding Fourier-transformed EXAFS magnitude |χ(R)|.

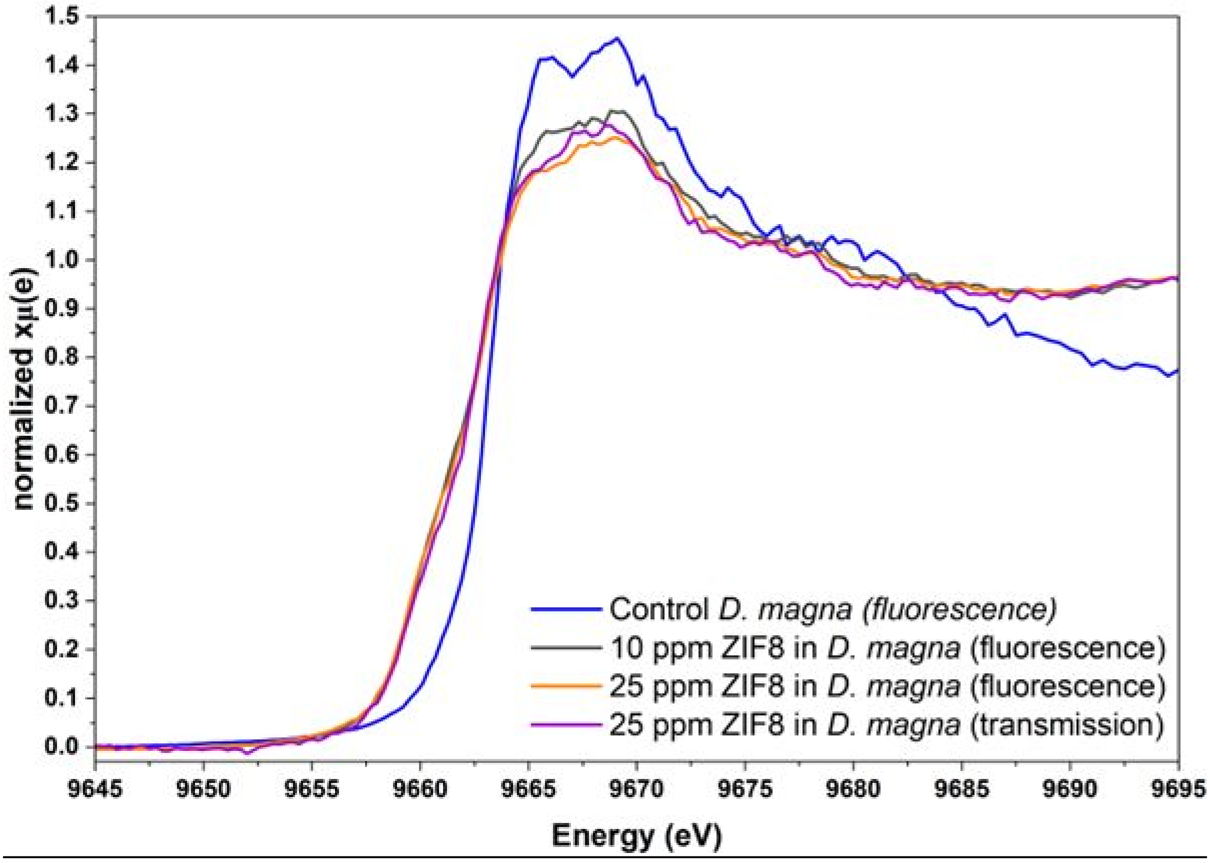

Figure S7. Zn K-edge XANES spectra for unexposed (control) *D. magna* and for daphnids exposed to 10 ppm (10 μg mL^{-1}) and 25 ppm (25 μg mL^{-1}) BHW-aged ZIF-8. The exposed organisms show nearly identical XANES profiles, while the control exhibits a clearly distinct spectrum, confirming that the Zn signal in exposed daphnids originates from ZIF-8 rather than endogenous Zn. Minor differences in white-line intensity between the exposed samples-specifically the reduced intensity at 25 ppm arise from self-absorption effects at higher Zn concentrations, as supported by the strong transmission edge jump (~0.1) and comparison of fluorescence and transmission spectra for this specimen.

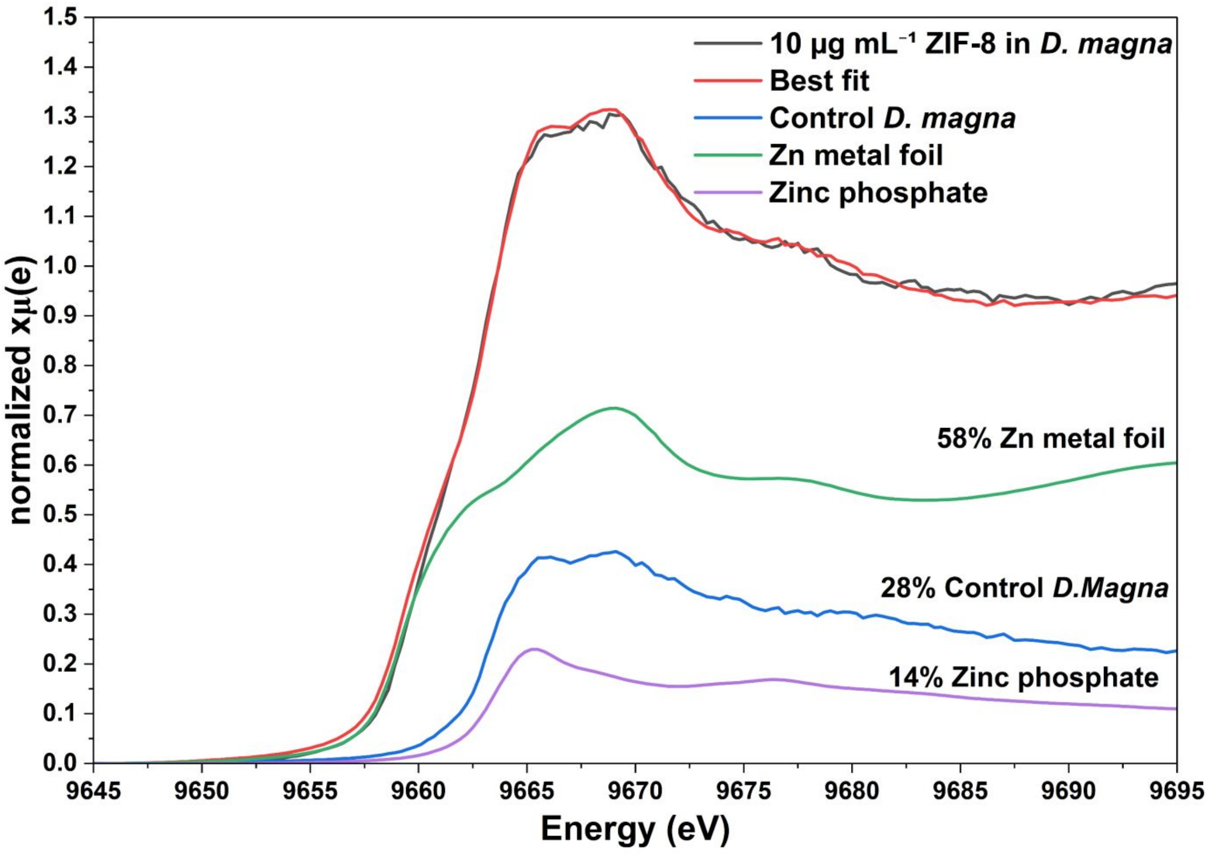

Figure S8. Linear combination fitting of the Zn K edge XANES spectrum collected on *D. magna* exposed to 10 µg mL⁻¹ BHW-aged ZIF-8 using Zn metal foil, unexposed *D. magna*, and zinc phosphate as reference components. Substituting zinc phosphate with zinc sulfide yields an equivalently good fit, indicating that the data do not allow us to discriminate between these two species within the current resolution. Major components remain the same: metallic zinc and endogenous Zn from unexposed *D. magna.*

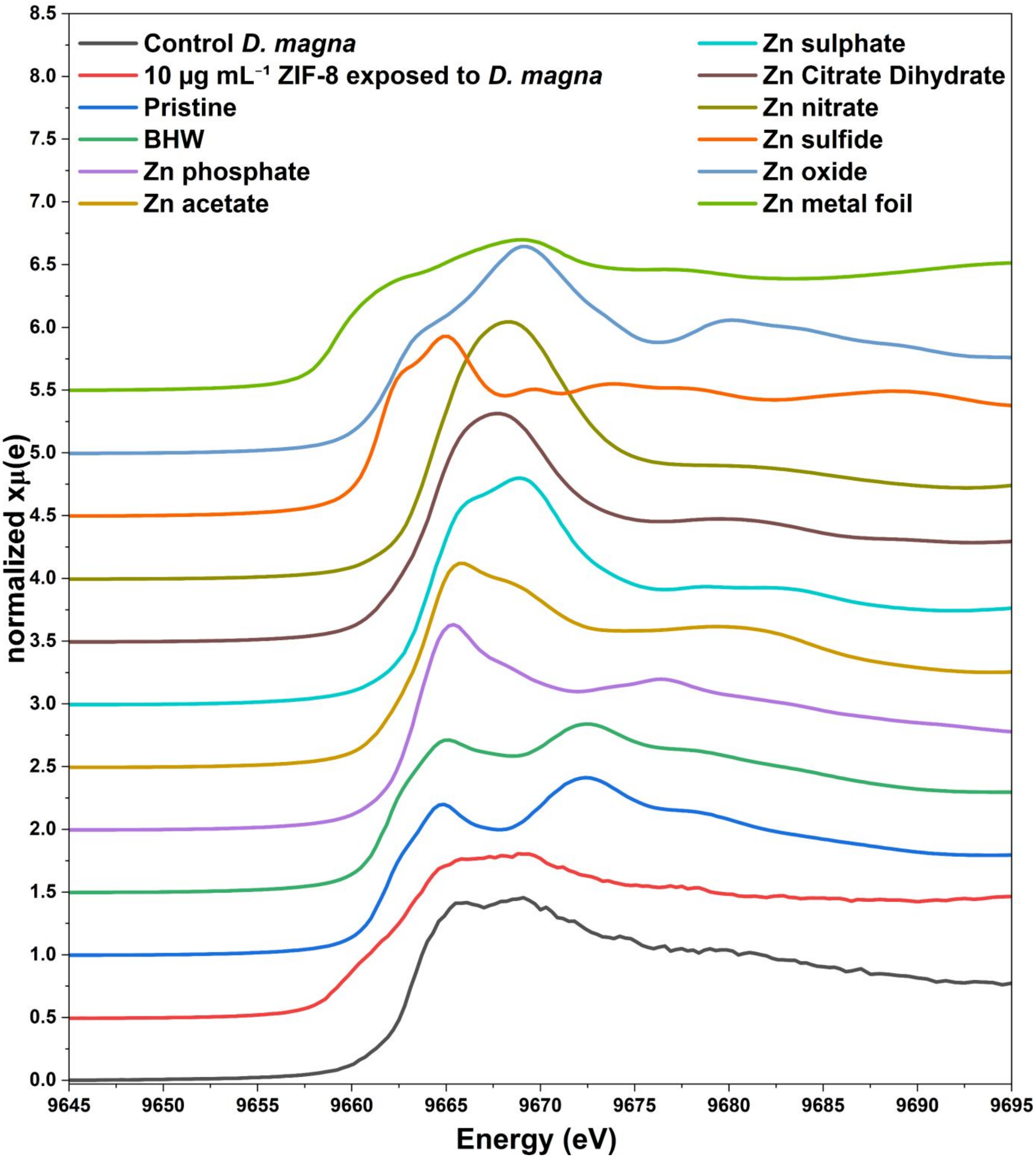

Figure S9. Comparison of Zn K-edge XANES spectra collected on unexposed (control) *D. magna* and on daphnid that was exposed to 10 µg mL^{-1} BHW-aged ZIF-8 with air exposed pristine and BHW-aged ZIF-8 and reference compounds.

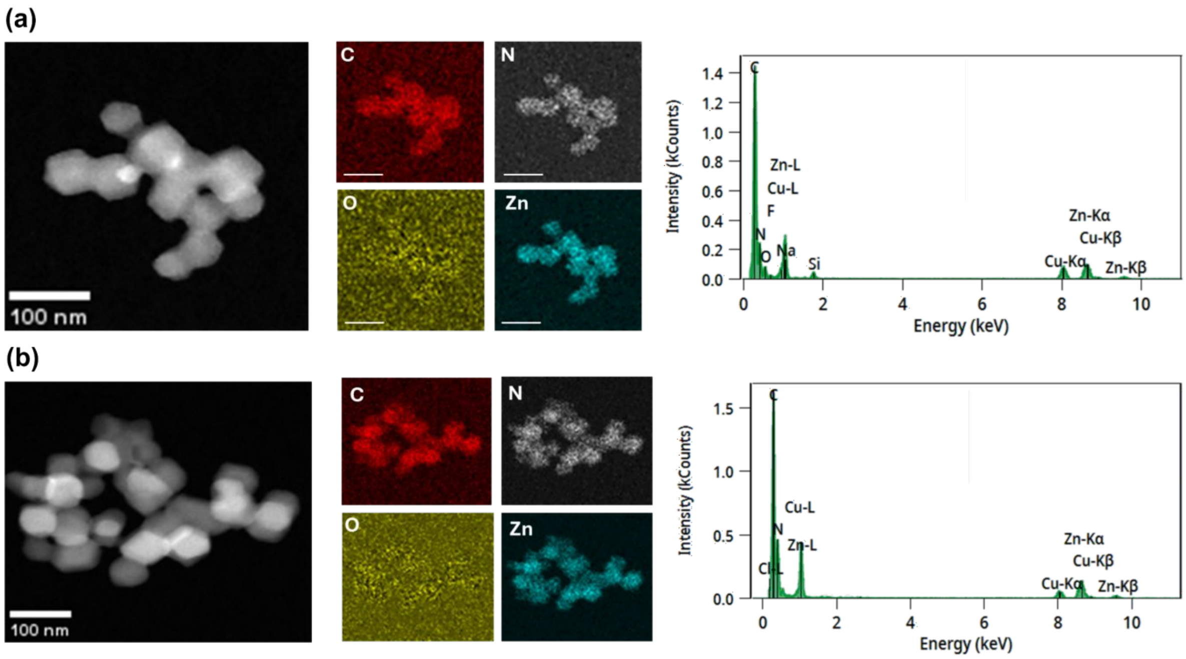

Figure S10. HAADF–STEM and EDS comparison of pristine and BHW-aged ZIF-8. HAADF–STEM image of pristine ZIF-8 nanoparticles (left; scale bar = 100 nm), element-specific STEM–EDS maps for C, N, O and Zn (centre; identical scale bars), and the corresponding EDS spectrum (right). Zn and N signals are strongly co-localised with the bright HAADF contrast, confirming a Zn–imidazolate framework with only minor O contribution, whereas C is more broadly distributed due to the carbon support film. Bottom row: equivalent HAADF–STEM, elemental maps and EDS spectrum for ZIF-8 aged 7 d in BHW. Nanoparticles remain faceted and Zn-rich, but the relative O signal increases while N/Zn intensity is slightly reduced, consistent with partial surface hydrolysis and formation of Zn–O(H) species at the expense of Zn–N, while retaining an overall ZIF-8-like composition.

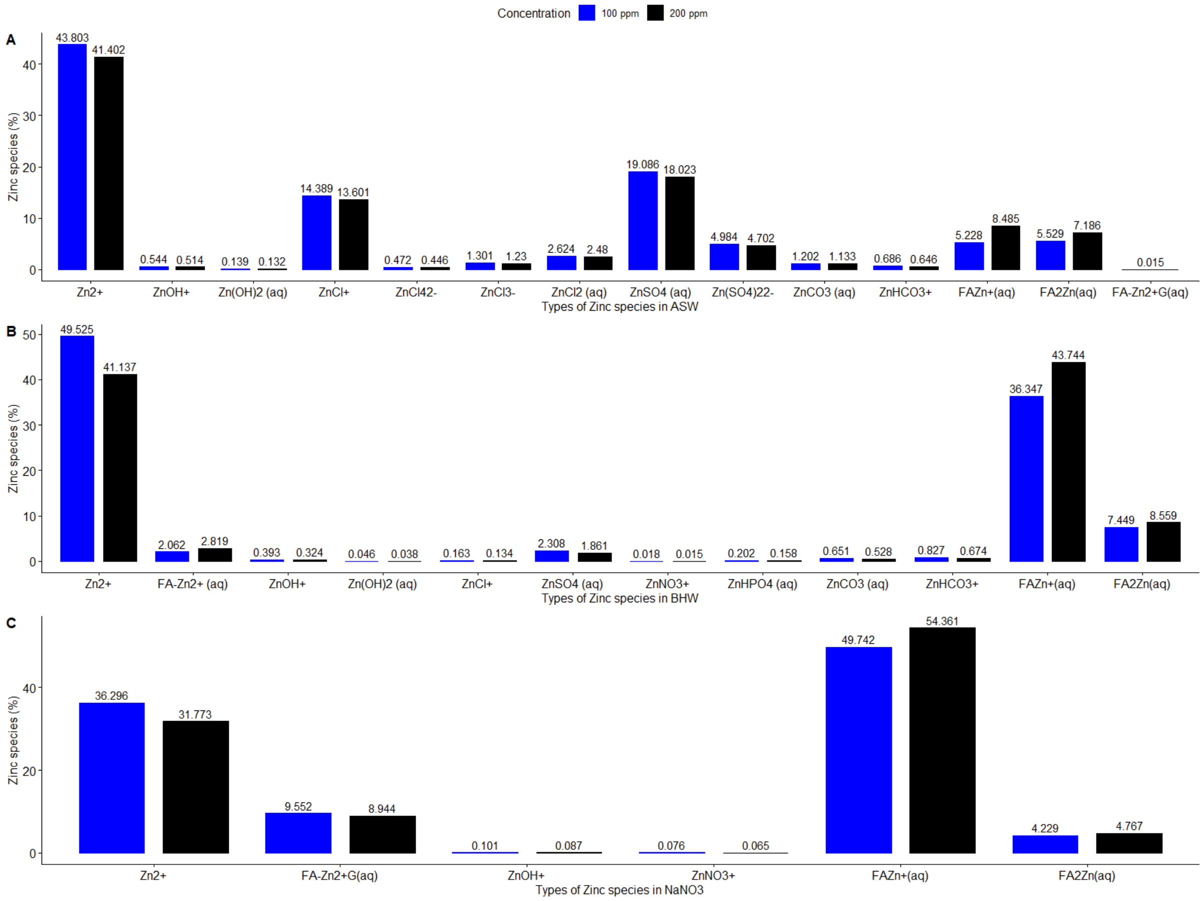

Figure S11. Equilibrium Zn speciation predicted using Visual MINTEQ 3.1 in (A) ASW, (B) 1 mM $NaNO_3$ (0.085 g L^{-1}), and (C) BHW; composition in Table S2. Bars show the percentage distribution of total dissolved Zn among the dominant inorganic species (e.g., Zn^{2+}, hydroxide-, chloride-, sulfate-, carbonate- and phosphate-complexes) and fulvic-acid-associated complexes ($FAZn^{+}$, FA_2Zn, FA–Zn^{2+}) at two total Zn loadings: 100 ppm (blue) and 200 ppm (black), where 1 ppm = 1 µg mL^{-1}.

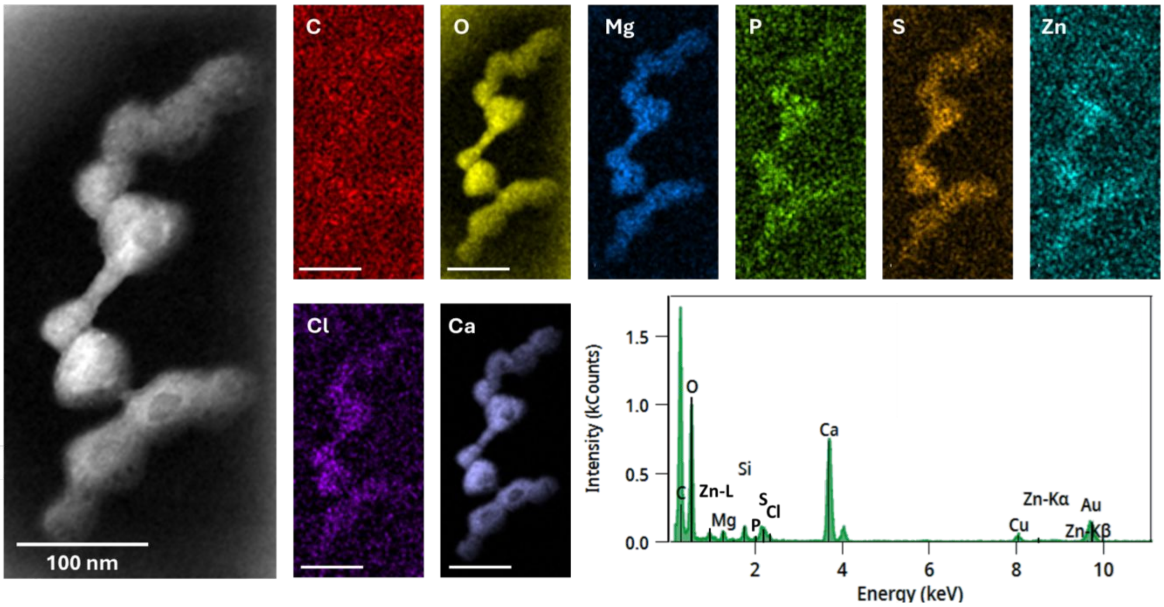

Figure S12. HAADF-STEM image of a representative excreted aggregate recovered after depuration, with corresponding STEM-EDS elemental maps (C, O, Mg, P, S, Zn, Cl, Ca) and an EDS spectrum (scale bars: 100 nm). Zn co-localises with P, S and Ca, consistent with the formation of mixed Zn-phosphate/carbonate biominerals or Zn bound to phosphorus- and sulphur-containing organic matrices rather than intact ZIF-8.

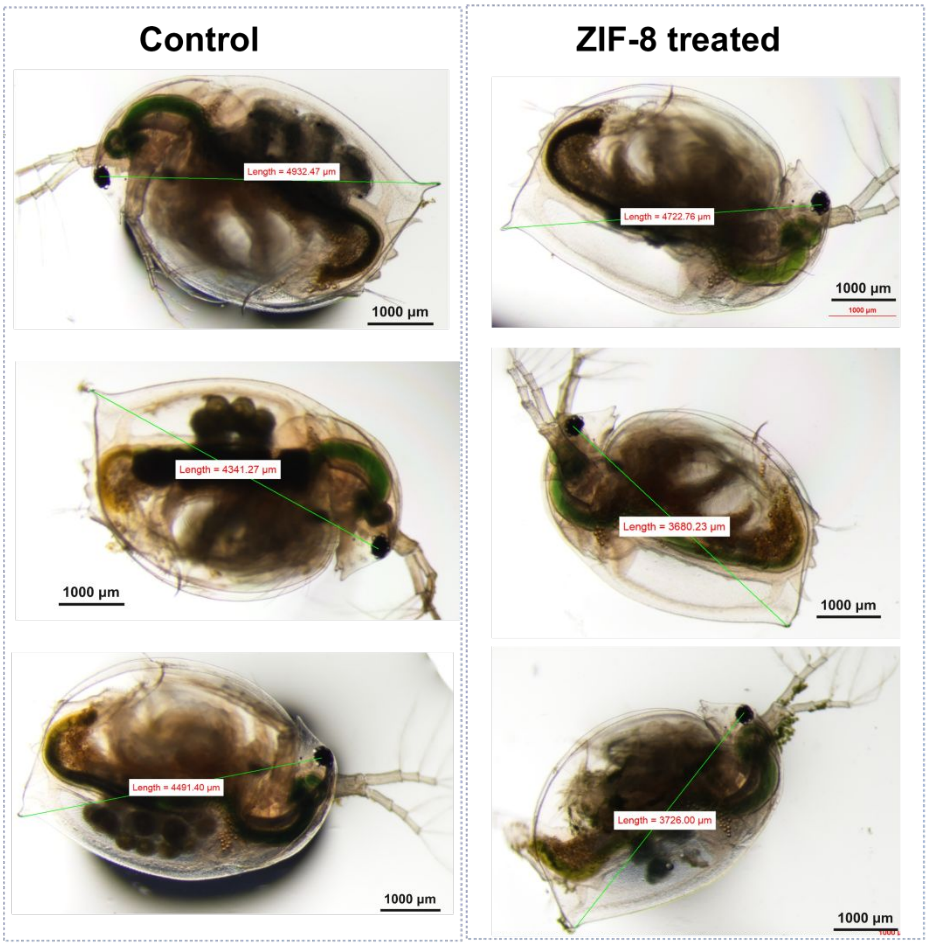

Figure S13. Representative bright-field micrographs of adult *D. magna* at the end of the 24-day chronic exposure (OECD TG 211-based, extended to capture delayed broods). Left panel: control organisms maintained in borehole water (BHW). Right panel: organisms exposed throughout the test to 0.10 µg mL^{-1} ZIF-8 (EC_{10} from the acute assay). The green line indicates body length measured from the top of the head to the base of the carapace; values (µm) are shown in the red labels. Control adults have lengths of 4932, 4341 and 4491 µm (mean ≈ 4.6 mm), whereas ZIF-8–exposed adults measure 4723, 3680 and 3726 µm (mean ≈ 4.0 mm), indicating a tendency towards reduced body size. In the ZIF-8 group, two females also displayed partial loss of the posterior tail spine (arrows, where visible), and one adult died on Day 18, pointing to subtle morphological and survival effects in addition to impaired reproduction.

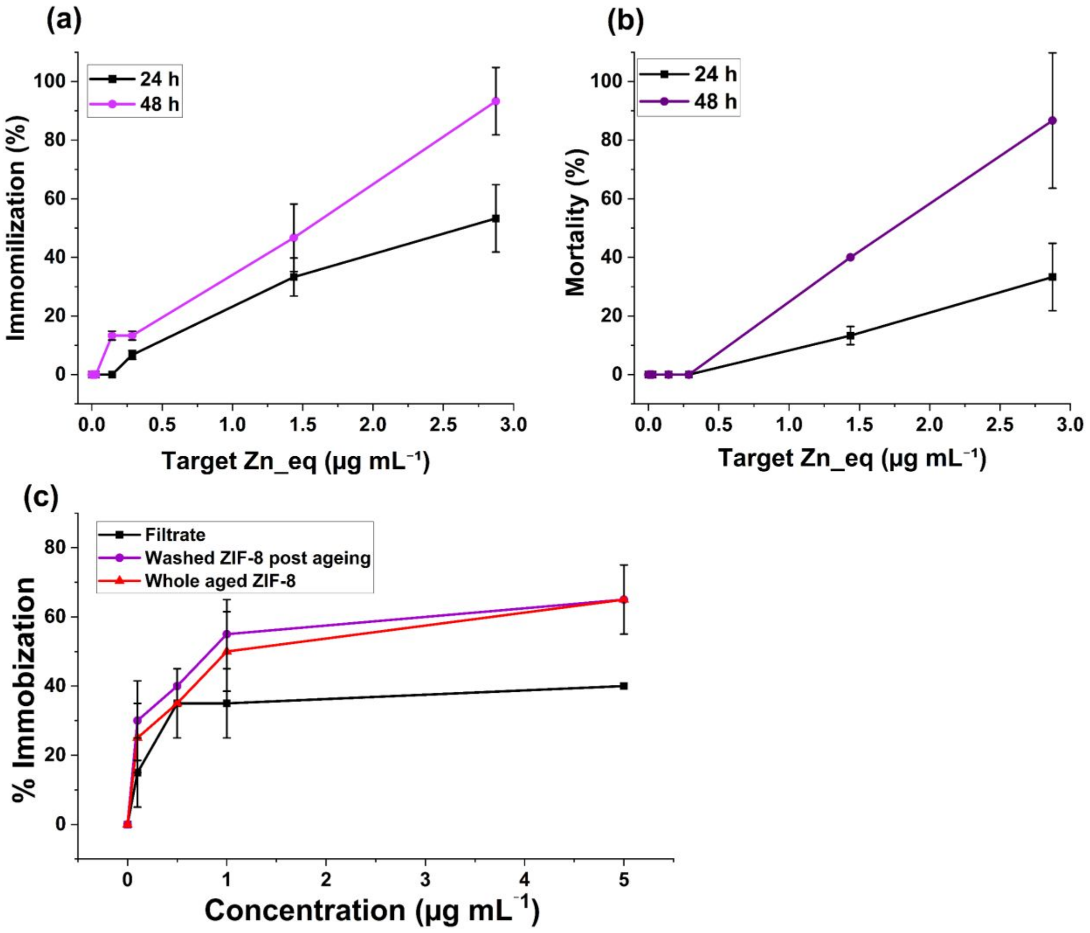

Figure S14. Acute toxicity of dissolved Zn and fractionated aged ZIF-8 to *D. magna*. (a) Mean immobilisation (%) ± SD after 24 h and 48 h for neonates exposed to $Zn(NO_3)_2{\cdot}6H_2O$, expressed as target Zn_eq (µg mL^{-1}) matching the Zn content of the ZIF-8 treatments (0.00287, 0.01437, 0.02873, 0.14365, 0.28731, 1.43654 and 2.87309 µg mL^{-1} Zn_eq, equivalent to 0.01, 0.05, 0.10, 0.50, 1.0, 5.0 and 10.0 µg mL^{-1} ZIF-8, respectively). (b) Corresponding mortality (%) ± SD at 24 h and 48 h. No immobilisation or mortality was observed up to 0.02873 µg mL^{-1} Zn_eq (≤0.10 µg mL^{-1} ZIF-8-equivalent); marked, concentration-dependent effects appeared only at ≥0.14365 µg mL^{-1} Zn_eq (≥0.50 µg mL^{-1} ZIF-8-equivalent), with 48 h immobilisation and mortality reaching ~93% and ~87% at 2.87309 µg mL^{-1} Zn_eq (10.0 µg mL^{-1} ZIF-8-equivalent). (c) 48 h fractionated acute immobilisation assay comparing three exposure fractions derived from the same 7-day aged ZIF-8 suspension (aged at 100 µg mL^{-1}): (i) particle-free filtrate collected using 3 kDa ultracentrifugation tubes, (ii) washed ZIF-8 post-ageing (aged particles/precipitates collected by centrifugation and washed three times before resuspension to minimise dissolved carryover), and (iii) the whole aged ZIF-8 suspension (unfractionated). Values are mean ± SD (n = 4 replicates, 5 neonates per replicate). The washed and whole fractions exhibit higher immobilisation than the filtrate, consistent with a major contribution from particle-associated transformed Zn pools.

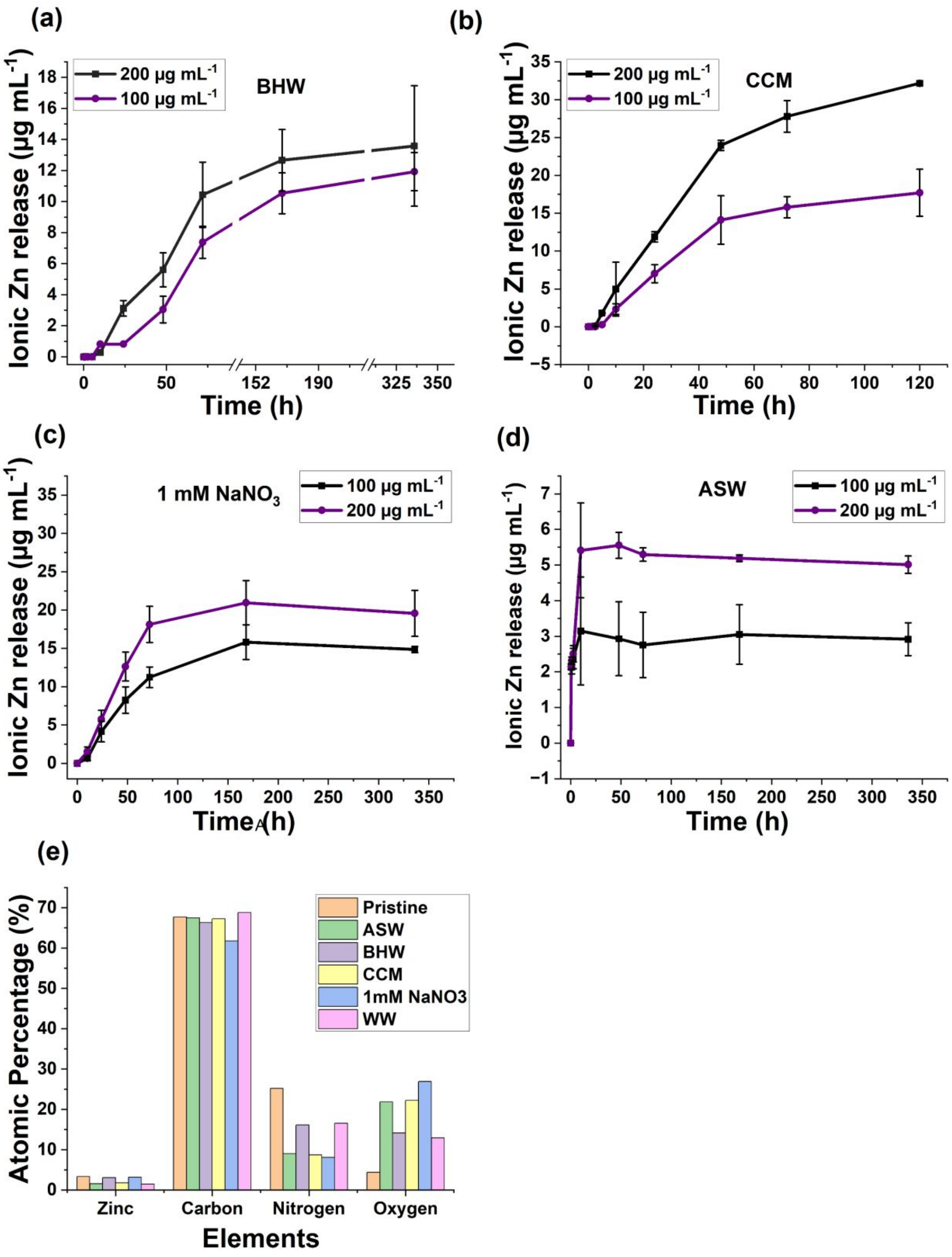

Figure S15. Time-resolved dissolved Zn release from ZIF-8 in aqueous media and corresponding XPS atomic composition after secondary ageing. (a–d) Dissolved Zn release (μg mL^{-1}; mean ± SD) from ZIF-8 at initial loadings of 100 and 200 μg mL^{-1} in (a) borehole water (BHW), (b) complete cell culture medium (CCM; DMEM + 10% FBS), (c) 1 mM $NaNO_3$, and (d) artificial seawater (ASW). (e) XPS atomic percentages (Zn, C, N, O) for pristine and aged ZIF-8 solids across media, highlighting medium-dependent N depletion and O enrichment consistent with surface restructuring. Quantitative timepoint values are summarised in Table S13 and XPS indices are provided in Table S14.

Table S1a. Exposure environments used in the hierarchical cascade: composition/conditions, pH (aligned with Visual MINTEQ where applicable), and relevance. The portion of table discussing the composition of BHW is reproduced from REFERENCE [4]. Copyright 2025 American Chemical Society.

Tier	Medium / condition	Key components / composition (as used here)	pH used in this study†	Relevance
Primary	Air exposure (7 d)	Ambient laboratory air exposure (no added gases)	—	Dry handling/storage analogue prior to water contact
Primary (stress test)	O_3 / NO_2 (ppm)	O_3 (10 ppm) and NO_2 (5 ppm) in air (accelerated oxidative stress test)	—	Boundary-condition stress test (not intended to represent ambient)
Secondary	1 mM $NaNO_3$	Sodium nitrate (1 mM) in deionised water	~7.0	Low-complexity electrolyte baseline
Secondary	ASW (Artificial seawater)	NaCl (24.60 g/L); $NaHCO_3$ (0.180 g/L); KCl (0.670 g/L); $CaCl_2$ (1.360 g/L); $MgSO_4·7H_2O$ (6.290 g/L)	~7.5	High ionic strength / major ions representative of marine conditions
Secondary	BHW (University of Birmingham EcoLab borehole water)	Ca^{2+} (~60 mg/L), Mg^{2+} (~30 mg/L), Na^+, Cl^-, HCO_3^-, NO_3^- (<10 mg/L), trace metals (Fe, Mn), low DOC	~7.0–7.5	Realistic hard freshwater matrix (carbonate/bicarbonate + trace phosphate)
Secondary	WW (Simulated wastewater)	Milk powder: 150 mg/L (COD 156 mg/L); starch: 80 mg/L (COD 80.6 mg/L); sodium acetate: 103 mg/L (COD 39.6 mg/L); yeast: 24 mg/L (COD 24 mg/L); NH_4Cl: 21.7 mg/L (6 mg/L N); urea: 12.8 mg/L (6 mg/L N); KH_2PO_4: 13.2 mg/L (3 mg/L P); $NaHCO_3$: 600 mg/L (300 mg/L as $CaCO_3$ alkalinity)	~7.0–7.4	Organic-/nutrient-rich effluent proxy (not used for MINTEQ speciation)

Secondary	CCM (Complete culture medium)	DMEM high glucose (**Sigma D6429**, 4.5 g/L glucose; L-glutamine; sodium pyruvate; sodium bicarbonate; phenol red) + **10% fetal bovine serum (FBS)**	~7.2–7.4	Protein-rich biological proxy to promote biomolecule/corona interactions (MilliporeSigma)
Tertiary	*Daphnia magna (D. magna)* (biotic tier)	*D. magna* exposures conducted in the relevant test medium (as specified in Methods; OECD-anchored acute/chronic endpoints)	—	Freshwater grazer model; links transformation to OECD TG 202/211 endpoints (OECD)

Table S1b. Summary of the aqueous media used in the secondary transformation experiments, showing approximate pH, ionic strength, organic matter content, dominant ionic species, phosphate, and major anion concentrations. Values are given as nominal preparation concentrations for defined media/recipes and as representative source-water values for University of Birmingham EcoLab[5] borehole water (BHW).

**Medium**	**pH**	**Approx. ionic strength**	**NOM / organics**	**Dominant ionic species**	**Major anions in the medium (mg L⁻¹)**	**Phosphate**
1 mM $NaNO_3$	7.0	~0.001 M	Negligible	Na^+, NO_3^-	NO_3^-: 62.01	Not added
ASW (artificial seawater)[6]	7.5	~0.7 M	Negligible	Na^+, Mg^{2+}, Ca^{2+}, K^+, Cl^-, SO_4^{2-}, carbonate species	Cl^-: 15697.6; SO_4^{2-}: 5519.2; carbonate species: 130.7	Not added
BHW (University of Birmingham EcoLab borehole water)	7.0-7.5	~0.005 M	Low DOC (1 mg L^{-1})	Ca^{2+}, Mg^{2+}, Na^+, K^+, HCO_3^-, Cl^-, SO_4^{2-}, NO_3^-, silicate, borate, low phosphate	Cl^-: 55.88; SO_4^{2-}: 44.23; NO_3^-: 12.40; HCO_3^-: 45.76; silicate: 6.01; borate: 24.00	~0.34 mg L^{-1} as PO_4-P (≈ 1.04 mg L^{-1} as PO_4^{3-} equivalent)[5]
WW (simulated wastewater)[7]	7.0-7.4	≥0.009 M	High; milk powder, starch, yeast, acetate, urea	NH_4^+, Na^+, K^+, HCO_3^-, phosphate, chloride, acetate + complex organics	HCO_3^-: 435.8; PO_4^{3-} equivalent: 9.2; Cl^-: 14.38; acetate: 74.13	~9.2 mg L^{-1} as PO_4^{3-} equivalent

CCM (DMEM + 10% FBS)	7.2-7.4	~0.17 M	Very high; proteins ~3–4.5 mg mL^{-1} + amino acids and other organics	Na^{+}, K^{+}, Ca^{2+}, Mg^{2+}, Cl^{-}, HCO_3^{-}, SO_4^{2-}, phosphate + serum biomolecules	Cl^{-}: ~4200.6; HCO_3^{-}: ~2687.4; SO_4^{2-}: ~77.95; PO_4^{3-} equivalent: ~85.8	~0.916 mM phosphate in DMEM

Table S2. Minteq 3.1 Model input parameters

Parameter	Concentration (mg L^{-1})
**Artificial Sea Water**	
pH	7.5
Na^+	9731.00
K^+	351.30
Ca^{2+}	491.03
Mg^{2+}	1397.03
Cl^-	15697.60
CO_3^{2-}	130.74
SO_4^{2-}	5519.16
**1mM $NaNO_3$**	
pH	7.0
Na^+	22.99
NO_3^-	62.01
**BHW**	
pH	7.0
Ca	29.99
Mg	11.19
K	3.91
Na	26.44
Cl	55.88
SO_4^{2-}	44.23
HPO_4^{2-}	0.98
NO_3^-	12.40
SiO_3^{2-}	6.01
HBO_3^{2-}	24.00
HCO_3^-	45.76
DOC	75.00 when Zn^{2+} was 25.00 and 150.00 when Zn^{2+} was 150.00

Table S3. Concentrations and activities of aqueous inorganic species of ZIF-8 MOF in ASW from visual MinteQ

Species	Concentration of ZIF-8 MOF 100 µg mL^{-1}			Concentration of ZIF-8 MOF 200 µg mL^{-1}		
	Concentration	Activity	Log activity	Concentration	Activity	Log activity
FA-(aq)	0.00043297	0.00043087	-3.366	0.00085327	0.00084919	-3.071
FA_2Ca(aq)	0.0000016	0.0000016	-5.796	2.8392E-06	2.8392E-06	-5.547
FA_2Zn(aq)	0.000021145	0.000021145	-4.675	0.000054964	0.000054964	-4.26
FACa+(aq)	0.000037707	0.000037524	-4.426	0.000070864	0.000070525	-4.152
FA-Ca^{2+} G(aq)	1.3243E-06	1.3243E-06	-5.878	2.5638E-06	2.5638E-06	-5.591
FA-H^+ G(aq)	1.2974E-11	1.2974E-11	-10.887	2.518E-11	2.518E-11	-10.599
FA-K^+ G(aq)	2.3579E-06	2.3579E-06	-5.627	4.5757E-06	4.5757E-06	-5.34
FAMg+ (aq)	0.00016995	0.00016912	-3.772	0.00031943	0.00031791	-3.498
FA-Mg^{2+} G(aq)	5.9689E-06	5.9689E-06	-5.224	0.000011557	0.000011557	-4.937
FA-Na+ G(aq)	0.00011224	0.00011224	-3.95	0.00021781	0.00021781	-3.662

FAZn+ (aq)	0.000019995	0.000019899	-4.701	0.000064903	0.000064593	-4.19
FA-Zn^{2+} G(aq)	3.1786E-08	3.1786E-08	-7.498	1.1664E-07	1.1664E-07	-6.933
HFA	0.00016262	0.00016262	-3.789	0.00031338	0.00031338	-3.504
Ca^{2+}	0.0069796	0.002034	-2.692	0.006961	0.0020288	-2.693
$CaCl^{+}$	0.0019969	0.0014672	-2.834	0.0019917	0.0014634	-2.835
$CaCO_3$ (aq)	5.5237E-06	6.2455E-06	-5.204	5.4945E-06	0.000006213	-5.207
$CaHCO_3^{+}$	0.000043998	0.000032327	-4.49	0.000043768	0.000032159	-4.493
$CaOH^{+}$	1.7286E-08	1.27E-08	-7.896	1.7239E-08	1.2666E-08	-7.897
$CaSO_4$ (aq)	0.0031846	0.0036007	-2.444	0.0031731	0.003588	-2.445
Cl^{-}	0.39086	0.28717	-0.542	0.39082	0.28716	-0.542
CO_3^{2-}	0.000006349	1.8502E-06	-5.733	6.3314E-06	1.8453E-06	-5.734
DOC (SHM)	0.00625	0.0070667	-2.151	0.0125	0.014135	-1.85
H^{+}	4.304E-08	3.1623E-08	-7.5	4.3039E-08	3.1623E-08	-7.5
$H_2CO_3^*$ (aq)	0.000078502	0.000088761	-4.052	0.000078288	0.000088525	-4.053
HCO_3^{-}	0.0016986	0.001248	-2.904	0.0016941	0.0012447	-2.905
HFA-dum	0.000018567	0.000018567	-4.731	0.000035724	0.000035724	-4.447
HSO_4^{-}	3.2502E-08	2.388E-08	-7.622	3.247E-08	2.3857E-08	-7.622
K^{+}	0.0078223	0.0057473	-2.241	0.0078208	0.0057464	-2.241
KCl (aq)	0.00073159	0.0008272	-3.082	0.00073137	0.00082701	-3.082
KOH (aq)	2.7644E-09	3.1256E-09	-8.505	2.7634E-09	3.1247E-09	-8.505
KSO_4^{-}	0.00042793	0.00031442	-3.502	0.00042744	0.00031406	-3.503
Mg^{2+}	0.031457	0.0091672	-2.038	0.031378	0.0091451	-2.039
$Mg_2CO_3^{2+}$	2.0758E-06	6.0491E-07	-6.218	2.0601E-06	6.0041E-07	-6.222
$MgCl^{+}$	0.014264	0.01048	-1.98	0.014229	0.010455	-1.981
$MgCO_3$ (aq)	0.000012477	0.000014108	-4.851	0.000012413	0.000014036	-4.853
$MgHCO_3^{+}$	0.00015971	0.00011734	-3.931	0.0001589	0.00011675	-3.933
$MgOH^{+}$	1.4845E-06	1.0907E-06	-5.962	1.4807E-06	0.000001088	-5.963
$MgSO_4$ (aq)	0.011401	0.012891	-1.89	0.011362	0.012847	-1.891
Na^{+}	0.37236	0.27358	-0.563	0.37228	0.27354	-0.563
NaCl (aq)	0.034826	0.039377	-1.405	0.034815	0.039367	-1.405
$NaCO_3^{-}$	0.000012829	9.4257E-06	-5.026	0.000012792	0.000009399	-5.027
$NaHCO_3$ (aq)	0.00015135	0.00017112	-3.767	0.00015091	0.00017064	-3.768
NaOH (aq)	9.5329E-08	1.0779E-07	-6.967	9.5293E-08	1.0775E-07	-6.968
$NaSO_4^{-}$	0.015813	0.011618	-1.935	0.015794	0.011605	-1.935
OH^{-}	4.2593E-07	3.1295E-07	-6.505	4.2587E-07	3.1291E-07	-6.505
SO_4^{2-}	0.026517	0.0077276	-2.112	0.026488	0.0077201	-2.112
Z-(6) (aq)	-0.00020532	-0.00020532	0	-0.00039807	-0.00039807	0
$Zn(CO_3)_2^{2-}$	1.1442E-08	3.3344E-09	-8.477	2.1516E-08	6.2707E-09	-8.203
$Zn(OH)_2$ (aq)	5.3233E-07	6.019E-07	-6.22	1.0061E-06	1.1377E-06	-5.944
$Zn(OH)_3^{-}$	8.1071E-11	5.9565E-11	-10.225	1.5322E-10	1.1258E-10	-9.949
$Zn(OH)_4^{2-}$	1.0138E-15	2.9544E-16	-15.53	1.9156E-15	5.583E-16	-15.253
$Zn(SO_4)_2^{2-}$	0.000019061	5.5548E-06	-5.255	0.000035963	0.000010482	-4.98
Zn^{2+}	0.00016752	0.000048818	-4.311	0.00031668	0.000092296	-4.035
Zn_2OH^{3+}	1.1953E-09	7.4582E-11	-10.127	4.2708E-09	2.6655E-10	-9.574
$ZnCl^{+}$	0.00005503	0.000040432	-4.393	0.00010403	0.000076437	-4.117
$ZnCl_2$ (aq)	0.000010035	0.000011347	-4.945	0.000018969	0.00002145	-4.669
$ZnCl_3^{-}$	4.9761E-06	3.6561E-06	-5.437	9.4059E-06	0.000006911	-5.16
$ZnCl_4^{2-}$	1.8057E-06	5.2622E-07	-6.279	3.4127E-06	9.9463E-07	-6.002
$ZnCO_3$ (aq)	4.5969E-06	5.1976E-06	-5.284	8.6672E-06	9.8006E-06	-5.009
$ZnHCO_3^{+}$	2.6223E-06	1.9267E-06	-5.715	4.9444E-06	3.6329E-06	-5.44
$ZnOH^{+}$	2.0793E-06	1.5278E-06	-5.816	3.9306E-06	0.000002888	-5.539
ZnSO4 (aq)	0.000072994	0.000082532	-4.083	0.00013786	0.00015588	-3.807

Table S4. Saturation indices of ZIF-8 MOF in ASW from visual MinteQ

Mineral	Concentration of ZIF-8 MOF 100 μg mL^{-1}		Concentration of ZIF-8 MOF 200 μg mL^{-1}	
	**log IAP**	**Sat. index**	log IAP	Sat. index
Anhydrite	-4.804	-0.444	-4.805	-0.445

Aragonite	-8.424	-0.088	-8.427	-0.091
Artinite	5.152	-4.448	5.149	-4.451
Bianchite	-6.471	-4.706	-6.195	-4.43
Brucite	12.946	-4.154	12.945	-4.155
$CaCO3xH_2O$(s)	-8.432	-1.288	-8.435	-1.29
Calcite	-8.424	0.055	-8.427	0.053
Dolomite (disordered)	-16.195	0.345	-16.199	0.341
Dolomite (ordered)	-16.195	0.895	-16.199	0.891
Epsomite	-4.205	-2.079	-4.207	-2.08
Goslarite	-6.479	-4.468	-6.203	-4.192
Gypsum	-4.82	-0.21	-4.821	-0.211
Halite	-1.105	-2.655	-1.105	-2.655
Huntite	-31.736	-1.768	-31.745	-1.777
Hydromagnesite	-18.168	-9.402	-18.178	-9.412
Hydrozincite	11.93	3.23	13.31	4.61
KCl(s)	-2.782	-3.682	-2.782	-3.682
Lime	12.3	-20.399	12.299	-20.4
Magnesite	-7.771	-0.311	-7.773	-0.313
$Mg(OH)_2$ (active)	12.946	-5.848	12.945	-5.849
$Mg2(OH)_3Cl:4H_2O$(s)	17.827	-8.173	17.825	-8.175
$MgCO_3:5H_2O$(s)	-7.81	-3.27	-7.813	-3.273
Mirabilite	-3.317	-2.203	-3.318	-2.204
Natron	-6.938	-5.627	-6.939	-5.628
Nesquehonite	-7.794	-3.124	-7.797	-3.127
Periclase	12.954	-8.63	12.953	-8.631
Portlandite	12.292	-10.412	12.291	-10.413
Smithsonite	-10.044	0.856	-9.769	1.131
Thenardite	-3.238	-3.559	-3.238	-3.56
Thermonatrite	-6.867	-7.504	-6.868	-7.505
Vaterite	-8.424	-0.511	-8.427	-0.513
Zincite	10.681	-0.549	10.957	-0.273
Zincosite	-6.423	-10.353	-6.147	-10.077
$Zn(OH)_2$ (am)	10.673	-1.801	10.949	-1.525
$Zn(OH)_2$ (beta)	10.673	-1.081	10.949	-0.805
$Zn(OH)_2$ (delta)	10.673	-1.171	10.949	-0.895
$Zn(OH)_2$ (epsilon)	10.673	-0.861	10.949	-0.585
$Zn(OH)_2$ (gamma)	10.673	-1.061	10.949	-0.785
$Zn_2(OH)_2SO_4$(s)	4.249	-3.251	4.802	-2.698
$Zn_2(OH)_3Cl$(s)	13.311	-1.88	13.865	-1.326
$Zn_3O(SO_4)_2$(s)	-2.166	-21.08	-1.337	-20.251
$Zn_4(OH)_6SO_4$(s)	25.595	-2.805	26.701	-1.699
$Zn_5(OH)_8Cl_2$(s)	37.296	-1.204	38.679	0.179
$ZnCl_2$(s)	-5.395	-12.445	-5.119	-12.169
$ZnCO_3$(s)	-10.044	0.756	-9.769	1.031
$ZnCO_3:1H_2O$(s)	-10.052	0.208	-9.777	0.483
$ZnSO_4:1H_2O$(s)	-6.431	-5.793	-6.155	-5.517

Table S5. Equilibrated mass distribution of **ZIF-8** MOF in Artificial Salt Water from visual MinteQ

**Concentration of ZIF-8 MOF 100 µg mL⁻¹**								
**Component**	**Dissolved inorganic**	**Bound to DOM**	**Total dissolved**	**% dissolved**	**Total sorbed**	**% sorbed**	**Total precipitated**	**% precipitated**
HFA(6)	0	0.000869	0.000869	100	0	0	0	0
Ca^{2+}	0.012211	4.06E-05	0.012251	100	0	0	0	0
Cl^{-}	0.44277	0	0.44277	100	0	0	0	0
CO_3^{2-}	0.002179	0	0.002179	100	0	0	0	0
H^{+}	0.002208	-0.00071	0.001502	100	0	0	0	0
K^{+}	0.008982	2.36E-06	0.008984	100	0	0	0	0
Mg^{2+}	0.0573	0.000176	0.057476	100	0	0	0	0
Na^{+}	0.42316	0.000112	0.42327	100	0	0	0	0
SO_4^{2-}	0.057454	0	0.057454	100	0	0	0	0
Zn^{2+}	0.000341	4.12E-05	0.000382	100	0	0	0	0
**Concentration of ZIF-8 MOF 200 µg mL⁻¹**								
HFA(6)	0	0.001737	0.001737	100	0	0	0	0
Ca^{2+}	0.012175	7.63E-05	0.012251	100	0	0	0	0
Cl^{-}	0.44277	0	0.44277	100	0	0	0	0
CO_3^{2-}	0.002179	0	0.002179	100	0	0	0	0
H^{+}	0.002201	-0.00142	0.000777	100	0	0	0	0
K^{+}	0.00898	4.58E-06	0.008984	100	0	0	0	0
Mg^{2+}	0.057145	0.000331	0.057476	100	0	0	0	0
Na^{+}	0.42306	0.000218	0.42327	100	0	0	0	0
SO_4^{2-}	0.057454	0	0.057454	100	0	0	0	0

Zn^{2+}	0.000645	0.00012	0.000765	100	0	0	0	0

Table S6. Concentrations and activities of aqueous inorganic species of ZIF-8 MOF in 1mM $NaNO_3$ from visual MinteQ

Species	Concentration of ZIF-8 MOF 100 µg mL⁻¹			Concentration of ZIF-8 MOF 200 µg mL⁻¹		
	Concentration	Activity	Log activity	Concentration	Activity	Log activity
FA-(aq)	0.00045345	0.00044672	-3.35	0.00087268	0.00085904	-3.066
FA_2Zn(aq)	0.000016174	0.000016174	-4.791	0.00003646	0.00003646	-4.438
FA-H^+ G(aq)	2.2403E-09	2.2403E-09	-8.65	3.1789E-09	3.1789E-09	-8.498
FA-Na^+ G(aq)	0.000006728	0.000006728	-5.172	9.4591E-06	9.4591E-06	-5.024
$FAZn^+$(aq)	0.00019024	0.00018741	-3.727	0.00041578	0.00040929	-3.388
FA-Zn^{2+} G(aq)	0.00003653	0.00003653	-4.437	0.000068409	0.000068409	-4.165
HFA	0.00019268	0.00019268	-3.715	0.00037607	0.00037607	-3.425
DOC (SHM)	0.00625	0.0062523	-2.204	0.0125	0.012506	-1.903
H^+	3.3066E-07	3.1623E-07	-6.5	3.328E-07	3.1623E-07	-6.5
HFA-dum	0.00010974	0.00010974	-3.96	0.00018266	0.00018266	-3.738
Na^+	0.00099302	0.00094968	-3.022	0.0009903	0.00094097	-3.026
$NaNO_3$ (aq)	2.5576E-07	2.5586E-07	-6.592	2.517E-07	2.5182E-07	-6.599
NaOH (aq)	3.805E-11	3.8064E-11	-10.419	3.7692E-11	3.7711E-11	-10.424
NO_3^-	0.00099953	0.00095591	-3.02	0.00099933	0.00094956	-3.022
OH^-	3.329E-08	3.1837E-08	-7.497	3.3503E-08	3.1834E-08	-7.497
Z-(6)(aq)	-0.00026322	-0.00026322	0	-0.00045689	-0.00045689	0
$Zn(NO_3)_2$ (aq)	5.316E-11	5.3179E-11	-10.274	8.9478E-11	8.9522E-11	-10.048
$Zn(OH)_2$ (aq)	1.4812E-08	1.4818E-08	-7.829	2.5261E-08	2.5273E-08	-7.597
$Zn(OH)_3^-$	1.5599E-13	1.4918E-13	-12.826	2.6776E-13	2.5442E-13	-12.594
$Zn(OH)_4^{2-}$	8.9987E-20	7.5276E-20	-19.123	1.5747E-19	1.2836E-19	-18.892
Zn^{2+}	0.00013881	0.00011612	-3.935	0.00024302	0.0001981	-3.703
Zn_2OH^{3+}	6.4148E-11	4.293E-11	-10.367	1.9787E-10	1.2493E-10	-9.903
$ZnNO_3^+$	2.9155E-07	2.7882E-07	-6.555	4.9728E-07	4.7251E-07	-6.326
$ZnOH^+$	3.8657E-07	3.697E-07	-6.432	6.6369E-07	6.3063E-07	-6.2

Table S8. Saturation indices of ZIF-8 MOF in 1mM $NaNO_3$ from visual MinteQ

Mineral	Concentration of ZIF-8 MOF 100 µg mL⁻¹		Concentration of ZIF-8 MOF 200 µg mL⁻¹	
	log IAP	Sat. index	log IAP	Sat. index
Zincite	9.065	-2.165	9.297	-1.933
Zn(NO3)2:6H2O(s)	-9.974	-13.29	-9.748	-13.064
Zn(OH)2 (am)	9.065	-3.409	9.297	-3.177
Zn(OH)2 (beta)	9.065	-2.689	9.297	-2.457
Zn(OH)2 (delta)	9.065	-2.779	9.297	-2.547
Zn(OH)2 (epsilon)	9.065	-2.469	9.297	-2.237
Zn(OH)2 (gamma)	9.065	-2.669	9.297	-2.437

Table S9. Equilibrated mass distribution of ZIF-8 MOF in 1mM $NaNO_3$ from visual MinteQ

Concentration of ZIF-8 MOF 100 µg mL⁻¹								
Component	Dissolved inorganic	Bound to DOM	Total dissolved	% dissolved	Total sorbed	% sorbed	Total precipitated	% precipitated

HFA(6)	0	0.000869	0.000869	100	0	0	0	0
H^+	-1.2E-07	-0.00068	-0.00068	100	0	0	0	0
Na^+	0.000993	6.73E-06	0.001	100	0	0	0	0
NO_3	0.001	0	0.001	100	0	0	0	0
Zn^{2+}	0.00014	0.000243	0.000382	100	0	0	0	0
**Concentration of ZIF-8 MOF 200 µg mL⁻¹**								
HFA(6)	0	0.001738	0.001738	100	0	0	0	0
H^+	-4.2E-07	-0.00136	-0.00136	100	0	0	0	0
Na^+	0.000991	9.46E-06	0.001	100	0	0	0	0
NO_3	0.001	0	0.001	100	0	0	0	0
Zn^{2+}	0.000244	0.000521	0.000765	100	0	0	0	0

Table S10. Concentrations and activities of aqueous inorganic species of ZIF-8 MOF in BHW from visual MinteQ

**Species**	**Concentration of ZIF-8 MOF 100 µg mL⁻¹**			**Concentration of ZIF-8 MOF 200 µg mL⁻¹**		
	**Concentration**	**Activity**	**Log activity**	**Concentration**	**Activity**	**Log activity**
FA-(aq)	0.00045119	0.00044466	-3.352	0.00086783	0.00085461	-3.068
FA_2Ca(aq)	2.5686E-07	2.5686E-07	-6.59	3.6777E-07	3.6777E-07	-6.434
FA_2Zn(aq)	0.000028489	0.000028489	-4.545	0.000065468	0.000065468	-4.184
$FACa^+$(aq)	0.000031363	0.00003091	-4.51	0.000044557	0.000043878	-4.358
FA-Ca^{2+} G(aq)	0.000027073	0.000027073	-4.567	0.000042763	0.000042763	-4.369
FA-H^+ G(aq)	3.5065E-10	3.5065E-10	-9.455	5.9944E-10	5.9944E-10	-9.222
FA-K^+ G(aq)	3.2193E-07	3.2193E-07	-6.492	5.4747E-07	5.4747E-07	-6.262
$FAMg^+$(aq)	0.000019469	0.000019188	-4.717	0.000027647	0.000027226	-4.565
FA-Mg^{2+} G(aq)	0.000016806	0.000016806	-4.775	0.000026534	0.000026534	-4.576
FA-Na^+ G(aq)	3.7034E-06	3.7034E-06	-5.431	6.2979E-06	6.2979E-06	-5.201
$FAZn^+$(aq)	0.00013901	0.000137	-3.863	0.00033458	0.00032949	-3.482
FA-Zn^{2+} G(aq)	7.8848E-06	7.8848E-06	-5.103	0.000021565	0.000021565	-4.666
HFA	0.00017022	0.00017022	-3.769	0.00033113	0.00033113	-3.48
$Ca(NO_3)_2$	5.0828E-16	5.0892E-16	-15.293	4.7873E-16	4.7938E-16	-15.319
Ca^{2+}	0.00065033	0.0004736	-3.325	0.00062395	0.00044893	-3.348
$CaCl^+$	1.8689E-06	1.7264E-06	-5.763	1.7714E-06	1.6314E-06	-5.787
$CaCO_3$ (aq)	2.4637E-07	2.4668E-07	-6.608	2.3117E-07	2.3149E-07	-6.635
$CaH_2BO_3^+$	6.5545E-08	6.0549E-08	-7.218	6.2325E-08	5.7401E-08	-7.241
$CaH_2PO_4^+$	4.905E-08	4.5312E-08	-7.344	4.451E-08	4.0994E-08	-7.387
$CaHCO_3^+$	4.3708E-06	4.0377E-06	-5.394	0.000004114	0.000003789	-5.421
$CaHPO_4$ (aq)	5.8568E-07	5.8642E-07	-6.232	5.2981E-07	5.3054E-07	-6.275
$CaNO_3^+$	2.9886E-07	2.7608E-07	-6.559	2.8325E-07	2.6088E-07	-6.584
$CaOH^+$	1.0298E-09	9.513E-10	-9.022	9.7901E-10	9.0166E-10	-9.045
$CaPO_4^-$	1.689E-08	1.5603E-08	-7.807	1.5327E-08	1.4116E-08	-7.85
$CaSO_4$ (aq)	0.000031729	0.00003177	-4.498	0.000029554	0.000029595	-4.529
Cl^-	0.001571	0.0014513	-2.838	0.0015708	0.0014467	-2.84
CO_3^{2-}	4.3097E-07	3.1385E-07	-6.503	4.3182E-07	3.107E-07	-6.508

DOC (SHM)	0.00625	0.0062579	-2.204	0.0125	0.012517	-1.902
H^+	1.0825E-07	0.0000001	-7	1.0858E-07	0.0000001	-7
$H_{10}(BO_3)_4^{2-}$	2.8348E-15	2.0644E-15	-14.685	2.8702E-15	2.0651E-15	-14.685
$H_2BO_3^-$	2.4273E-06	2.2423E-06	-5.649	2.4349E-06	2.2425E-06	-5.649
H_2CO_3* (aq)	0.00015037	0.00015056	-3.822	0.00014885	0.00014905	-3.827
$H_2PO_4^-$	4.6263E-06	4.2737E-06	-5.369	4.4287E-06	4.0788E-06	-5.389
$H_2SiO_4^{2-}$	2.6788E-13	1.9508E-13	-12.71	2.7116E-13	1.951E-13	-12.71
H_3BO_3	0.00038561	0.0003861	-3.413	0.0003856	0.00038613	-3.413
H_3PO_4	6.0014E-11	6.009E-11	-10.221	5.7272E-11	5.7351E-11	-10.241
$H_3SiO_4^-$	3.3469E-07	3.0918E-07	-6.51	3.3574E-07	3.0921E-07	-6.51
H_4SiO_4	0.00021363	0.0002139	-3.67	0.00021363	0.00021392	-3.67
$H_4SiO_4SO_4^{2-}$	2.4805E-08	1.8064E-08	-7.743	2.4675E-08	1.7754E-08	-7.751
$H_5(BO_3)_2^-$	7.9768E-10	7.3688E-10	-9.133	8.0023E-10	7.3701E-10	-9.133
$H_8(BO_3)_3^-$	3.0798E-11	2.8451E-11	-10.546	3.0899E-11	2.8458E-11	-10.546
HCO_3^-	0.0007247	0.00066946	-3.174	0.00071959	0.00066274	-3.179
HFA-dum	0.000080624	0.000080624	-4.094	0.00014339	0.00014339	-3.843
HPO_4^{2-}	3.7199E-06	0.000002709	-5.567	3.5934E-06	2.5855E-06	-5.587
HSO_4^-	3.0977E-09	2.8616E-09	-8.543	3.0534E-09	2.8121E-09	-8.551
K^+	0.000099386	0.000091811	-4.037	0.000099166	0.000091331	-4.039
K_2HPO_4 (aq)	3.0412E-13	3.045E-13	-12.516	2.872E-13	2.8759E-13	-12.541
K_2PO4^-	1.8968E-17	1.7522E-17	-16.756	1.7969E-17	1.6549E-17	-16.781
KCl (aq)	6.6695E-08	6.6779E-08	-7.175	6.6131E-08	6.6222E-08	-7.179
KH_2PO_4 (aq)	7.8189E-10	7.8288E-10	-9.106	7.4226E-10	7.4329E-10	-9.129
$KHPO_4^-$	2.0423E-09	1.8867E-09	-8.724	1.9449E-09	1.7913E-09	-8.747
KNO_3 (aq)	1.0914E-08	1.0927E-08	-7.961	1.0821E-08	1.0836E-08	-7.965
KOH (aq)	1.6042E-11	1.6062E-11	-10.794	1.5954E-11	1.5976E-11	-10.797
KPO_4^{2-}	3.8764E-14	2.8229E-14	-13.549	3.725E-14	2.6801E-14	-13.572
KSO_4^-	2.0603E-07	1.9033E-07	-6.72	2.0202E-07	1.8606E-07	-6.73
Mg^{2+}	0.00040371	0.000294	-3.532	0.00038715	0.00027856	-3.555
$Mg_2CO_3^{2+}$	1.4492E-10	1.0554E-10	-9.977	1.3036E-10	9.3795E-11	-10.028
$MgCl^+$	1.8387E-06	1.6986E-06	-5.77	0.000001742	1.6043E-06	-5.795
$MgCO_3$ (aq)	7.665E-08	7.6747E-08	-7.115	7.1889E-08	7.1988E-08	-7.143
$MgH_2BO_3^+$	2.4517E-08	2.2648E-08	-7.645	2.3302E-08	2.1461E-08	-7.668
$MgHCO_3^+$	2.1852E-06	2.0187E-06	-5.695	2.0559E-06	1.8935E-06	-5.723
$MgHPO_4$ (aq)	5.0187E-07	5.0251E-07	-6.299	4.5379E-07	4.5442E-07	-6.343
$MgOH^+$	1.2181E-08	1.1253E-08	-7.949	1.1575E-08	1.0661E-08	-7.972
$MgPO_4^-$	1.639E-10	1.5141E-10	-9.82	1.4866E-10	1.3692E-10	-9.864
$MgSO_4$ (aq)	0.000015646	0.000015666	-4.805	0.000014567	0.000014587	-4.836
Na^+	0.0011433	0.0010562	-2.976	0.0011408	0.0010506	-2.979
Na_2HPO_4 (aq)	2.6589E-11	2.6623E-11	-10.575	2.511E-11	2.5144E-11	-10.6
$Na_2PO_4^-$	5.3665E-15	4.9575E-15	-14.305	5.0837E-15	4.6821E-15	-14.33
NaCl (aq)	7.6723E-07	7.682E-07	-6.115	7.6074E-07	7.6178E-07	-6.118
$NaCO_3^-$	6.6817E-09	6.1724E-09	-8.21	6.5999E-09	6.0785E-09	-8.216
NaH_2BO_3 (aq)	3.7143E-09	3.719E-09	-8.43	3.6948E-09	3.6999E-09	-8.432
NaH_2PO_4 (aq)	8.9945E-09	9.0059E-09	-8.045	8.5387E-09	8.5504E-09	-8.068
$NaHCO_3$ (aq)	3.5392E-07	3.5437E-07	-6.451	3.485E-07	3.4898E-07	-6.457
$NaHPO_4^-$	3.6388E-08	3.3615E-08	-7.473	3.4652E-08	3.1915E-08	-7.496
$NaNO_3$ (aq)	5.4802E-08	5.4872E-08	-7.261	5.4338E-08	5.4413E-08	-7.264
NaOH (aq)	1.3369E-10	1.3386E-10	-9.873	1.3296E-10	1.3314E-10	-9.876
$NaPO_4^{2-}$	4.4592E-13	3.2474E-13	-12.488	4.285E-13	3.0831E-13	-12.511
$NaSO_4^-$	1.8398E-06	1.6995E-06	-5.77	0.000001804	1.6615E-06	-5.78
NO_3^-	0.00019955	0.00018434	-3.734	0.00019952	0.00018376	-3.736
OH^-	1.0898E-07	1.0067E-07	-6.997	1.093E-07	1.0066E-07	-6.997
PO_4^{3-}	2.3318E-11	1.1424E-11	-10.942	2.2867E-11	1.0903E-11	-10.962

SO_4^{2-}	0.0004021	0.00029282	-3.533	0.00039995	0.00028776	-3.541
Z-(6)(aq)	-0.00026135	-0.00026135	0	-0.00046104	-0.00046104	0
$Zn(CO_3)_2^{2-}$	3.7225E-10	2.7109E-10	-9.567	6.0605E-10	4.3606E-10	-9.36
$Zn(H_2BO_3)_2$ (aq)	8.1754E-12	8.1858E-12	-11.087	1.3419E-11	1.3438E-11	-10.872
$Zn(NO_3)_2$ (aq)	2.3461E-12	2.3491E-12	-11.629	3.8261E-12	3.8314E-12	-11.417
Zn(OH)2 (aq)	1.7576E-07	1.7599E-07	-6.755	2.8839E-07	2.8879E-07	-6.539
$Zn(OH)_3^-$	6.0649E-12	5.6026E-12	-11.252	9.9811E-12	9.1926E-12	-11.037
$Zn(OH)_4^{2-}$	1.2275E-17	8.9392E-18	-17.049	2.0383E-17	1.4666E-17	-16.834
$Zn(SO_4)_2^{2-}$	3.0946E-08	2.2536E-08	-7.647	4.9647E-08	3.5722E-08	-7.447
Zn^{2+}	0.0001894	0.00013793	-3.86	0.00031464	0.00022639	-3.645
Zn_2OH^{3+}	3.9095E-10	1.9153E-10	-9.718	1.082E-09	5.1591E-10	-9.287
$ZnCl^+$	6.2494E-07	5.7731E-07	-6.239	1.0256E-06	9.4458E-07	-6.025
$ZnCl_2$ (aq)	8.1772E-10	8.1876E-10	-9.087	1.3336E-09	1.3354E-09	-8.874
$ZnCl_3^-$	1.4432E-12	1.3332E-12	-11.875	2.3536E-12	2.1677E-12	-11.664
$ZnCl_4^{2-}$	1.3316E-15	9.6972E-16	-15.013	2.1845E-15	1.5717E-15	-14.804
$ZnCO_3$ (aq)	2.4879E-06	2.4911E-06	-5.604	4.0421E-06	4.0476E-06	-5.393
$ZnH_2BO_3^+$	8.3329E-09	7.6977E-09	-8.114	1.3719E-08	1.2636E-08	-7.898
$ZnHCO_3^+$	0.000003161	0.00000292	-5.535	5.1516E-06	4.7446E-06	-5.324
$ZnHPO_4$ (aq)	7.7075E-07	7.7173E-07	-6.113	1.2072E-06	1.2089E-06	-5.918
$ZnNO_3^+$	6.9138E-08	6.3868E-08	-7.195	1.1346E-07	1.045E-07	-6.981
$ZnOH^+$	1.5032E-06	1.3886E-06	-5.857	2.4743E-06	2.2789E-06	-5.642
$ZnSO_4$ (aq)	8.8251E-06	8.8363E-06	-5.054	0.000014233	0.000014253	-4.846

Table S11. Saturation indices of Zinc MOF in BHW from visual MinteQ

Mineral	Concentration of ZIF-8 MOF 100 μg mL⁻¹		Concentration of ZIF-8 MOF 200 μg mL⁻¹	
	log IAP	Sat. index	log IAP	Sat. index
Anhydrite	-6.858	-2.498	-6.889	-2.529
Aragonite	-9.828	-1.492	-9.855	-1.519
Artinite	0.433	-9.167	0.382	-9.218
Bianchite	-7.394	-5.629	-7.187	-5.422
Brucite	10.468	-6.632	10.445	-6.655
$Ca_3(PO_4)_2$ (am1)	-31.858	-6.358	-31.968	-6.468
$Ca_3(PO_4)_2$ (am2)	-31.858	-3.608	-31.968	-3.718
$Ca_3(PO_4)_2$ (beta)	-31.858	-2.938	-31.968	-3.048
$Ca_4H(PO_4)_3{:}3H_2O$(s)	-53.125	-5.175	-53.279	-5.329
$CaCO_3xH_2O$(s)	-9.828	-2.684	-9.856	-2.711
$CaHPO_4$(s)	-21.267	-1.992	-21.31	-2.035
$CaHPO_4{:}2H_2O$(s)	-21.267	-2.272	-21.31	-2.315
Calcite	-9.828	-1.348	-9.855	-1.376
Chalcedony	-3.67	-0.12	-3.67	-0.12
Chrysotile	24.065	-8.135	23.995	-8.205
Cristobalite	-3.67	-0.32	-3.67	-0.32
Dolomite (disordered)	-19.863	-3.323	-19.918	-3.378
Dolomite (ordered)	-19.863	-2.773	-19.918	-2.828
Epsomite	-7.066	-4.939	-7.097	-4.97
Goslarite	-7.394	-5.383	-7.187	-5.176
Gypsum	-6.858	-2.248	-6.889	-2.279
Halite	-5.815	-7.365	-5.818	-7.368
Huntite	-39.933	-9.965	-40.044	-10.076
Hydromagnesite	-29.672	-20.906	-29.807	-21.041
Hydroxyapatite	-42.45	1.883	-42.627	1.706
Hydrozincite	9.691	0.991	10.758	2.058
KCl(s)	-6.875	-7.775	-6.879	-7.779
Lime	10.675	-22.024	10.652	-22.047
Magnesite	-10.035	-2.575	-10.063	-2.603
$Mg(OH)_2$ (active)	10.468	-8.326	10.445	-8.349
$Mg_2(OH)_3Cl{:}4H_2O$(s)	11.098	-14.902	11.05	-14.95
$Mg_3(PO_4)_2$(s)	-32.479	-9.199	-32.59	-9.31
$MgCO_3{:}5H_2O$(s)	-10.035	-5.495	-10.063	-5.523

$MgHPO_4{:}3H_2O(s)$	-21.474	-3.299	-21.518	-3.343
Mirabilite	-9.487	-8.373	-9.499	-8.385
Natron	-12.457	-11.146	-12.466	-11.155
Nesquehonite	-10.035	-5.365	-10.063	-5.393
Periclase	10.468	-11.116	10.445	-11.139
Portlandite	10.675	-12.029	10.652	-12.052
Quartz	-3.67	0.33	-3.67	0.33
Sepiolite	9.927	-5.833	9.881	-5.879
Sepiolite (A)	9.927	-8.853	9.881	-8.899
SiO2 (am,gel)	-3.67	-0.96	-3.67	-0.96
SiO2 (am,ppt)	-3.67	-0.93	-3.67	-0.93
Smithsonite	-10.364	0.536	-10.153	0.747
Thenardite	-9.486	-9.808	-9.498	-9.82
Thermonatrite	-12.456	-13.093	-12.465	-13.102
Vaterite	-9.828	-1.914	-9.855	-1.942
Zincite	10.14	-1.09	10.355	-0.875
Zincosite	-7.394	-11.323	-7.186	-11.116
$Zn(BO_2)_2(s)$	3.313	-4.977	3.529	-4.761
$Zn(NO_3)_2{:}6H_2O(s)$	-11.33	-14.645	-11.117	-14.432
$Zn(OH)_2$ (am)	10.14	-2.334	10.355	-2.119
$Zn(OH)_2$ (beta)	10.14	-1.614	10.355	-1.399
$Zn(OH)_2$ (delta)	10.14	-1.704	10.355	-1.489
$Zn(OH)_2$ (epsilon)	10.14	-1.394	10.355	-1.179
$Zn(OH)_2$ (gamma)	10.14	-1.594	10.355	-1.379
$Zn_2(OH)_2SO_4(s)$	2.746	-4.754	3.169	-4.331
$Zn_2(OH)_3Cl$ (s)	10.441	-4.75	10.87	-4.321
$Zn_3(PO_4)_2{:}4H_2O(s)$	-33.466	1.954	-32.861	2.559
$Zn_3O(SO_4)_2(s)$	-4.648	-23.561	-4.017	-22.931
$Zn_4(OH)_6SO_4(s)$	23.025	-5.375	23.878	-4.522
$Zn_5(OH)_8Cl_2(s)$	31.021	-7.479	32.094	-6.406
$ZnCl_2(s)$	-9.537	-16.587	-9.324	-16.374
$ZnCO_3(s)$	-10.364	0.436	-10.153	0.647
$ZnCO_3{:}H_2O(s)$	-10.364	-0.104	-10.153	0.107
$ZnSO_4{:}H_2O(s)$	-7.394	-6.756	-7.186	-6.548

Table S12. Equilibrated mass distribution of **ZIF-8** MOF in BHW from visual MinteQ

**Concentration of ZIF-8 MOF 100 μg mL⁻¹**								
**Component**	**Dissolved inorganic**	**Bound to DOM**	**Total dissolved**	**% dissolved**	**Total sorbed**	**% sorbed**	**Total precipitated**	**% precipitated**
HFA(6)	0	0.000869	0.000869	100	0	0	0	0
Ca^{2+}	0.00069	5.87E-05	0.000748	100	0	0	0	0
Cl^-	0.001576	0	0.001576	100	0	0	0	0
CO_3^{2-}	0.000888	0	0.000888	100	0	0	0	0
H^+	0.001046	-0.0007	0.000347	100	0	0	0	0
H_3BO_3	0.000388	0	0.000388	100	0	0	0	0
H_4SiO_4	0.000214	0	0.000214	100	0	0	0	0
K^+	9.97E-05	3.22E-07	1E-04	100	0	0	0	0
Mg^{2+}	0.000424	3.63E-05	0.00046	100	0	0	0	0
Na^+	0.001146	3.7E-06	0.00115	100	0	0	0	0
NO_3^-	0.0002	0	0.0002	100	0	0	0	0

PO_4^{3-}	1.03E-05	0	1.03E-05	100	0	0	0	0
SO_4^{2-}	0.00046	0	0.00046	100	0	0	0	0
Zn^{2+}	0.000207	0.000175	0.000382	100	0	0	0	0
**Concentration of ZIF-8 MOF 200 μg mL⁻¹**								
HFA(6)	0	0.001737	0.001737	100	0	0	0	0
Ca^{2+}	0.000661	8.77E-05	0.000748	100	0	0	0	0
Cl^-	0.001576	0	0.001576	100	0	0	0	0
CO_3^{2-}	0.000885	0	0.000885	100	0	0	0	0
H^+	0.001038	-0.00141	-0.00037	100	0	0	0	0
H_3BO_3	0.000388	0	0.000388	100	0	0	0	0
H_4SiO_4	0.000214	0	0.000214	100	0	0	0	0
K^+	9.94E-05	5.47E-07	1E-04	100	0	0	0	0
Mg^{2+}	0.000406	5.42E-05	0.00046	100	0	0	0	0
Na^+	0.001144	6.3E-06	0.00115	100	0	0	0	0
NO_3^-	0.0002	0	0.0002	100	0	0	0	0
PO_4^{3-}	1.03E-05	0	1.03E-05	100	0	0	0	0
SO_4^{2-}	0.00046	0	0.00046	100	0	0	0	0
Zn^{2+}	0.000343	0.000422	0.000765	100	0	0	0	0

Table S13. Dissolved Zn release (μg mL⁻¹; mean ± SD) from ZIF-8 in aqueous media at harmonised timepoints. *Notes:* For CCM, the time series ends at 120 h. For ASW, measurements were not made at 24 h and 120 h (see Figure S15). For BHW and $NaNO_3$, 120 h was not sampled. WW dissolved Zn quantification was not reliable due to matrix interference (see text).

100 and 200 denote initial ZIF-8 loadings in μg mL⁻¹.

**Medium**	**24 h (100)**	**24 h (200)**	**72 h (100)**	**72 h (200)**	**120 h (100)**	**120 h (200)**	**336 h (100)**	**336 h (200)**
**BHW**	**0.82 ± 0.00**	**3.12 ± 0.50**	**7.38 ± 1.03**	**10.44 ± 2.10**	**—**	**—**	**11.93 ± 1.23**	**13.59 ± 3.88**
**CCM**	**7.01 ± 1.20**	**11.89 ± 0.68**	**15.80 ± 1.40**	**27.80 ± 2.09**	**17.70 ± 3.10**	**32.18 ± 0.19**	**—**	**—**
**ASW**	**—**	**—**	**2.76 ± 0.92**	**5.29 ± 0.19**	**—**	**—**	**2.92 ± 0.46**	**5.01 ± 0.24**
**1 mM $NaNO_3$**	**4.16 ± 1.34**	**5.76 ± 1.17**	**11.24 ± 1.34**	**18.12 ± 2.36**	**—**	**—**	**14.86 ± 0.40**	**19.58 ± 3.01**

Table S14. XPS atomic percentages and derived restructuring indices for ZIF-8 aged in aqueous media. Indices quantify oxygenation and N depletion relative to pristine ZIF-8 (surface-sensitive operational proxies).

Medium	Zn (at%)	C (at%)	N (at%)	O (at%)	O/N	ΔN vs pristine (at%)	ΔO vs pristine (at%)
**Pristine**	3.33	67.70	25.19	4.41	0.175	0.00	0.00
**ASW**	1.60	67.49	9.06	21.84	2.41	−16.13	+17.43
**BHW**	3.07	66.29	16.12	14.15	0.88	−9.07	+9.74
**CCM**	1.81	67.22	8.71	22.27	2.56	−16.48	+17.86
**$NaNO_3$**	3.22	61.77	8.11	26.89	3.32	−17.08	+22.48
**WW**	1.49	68.80	16.59	12.93	0.78	−8.60	+8.52

Table S15. 48 h fractionated acute immobilisation of *D. magna* exposed to aged ZIF-8 suspensions (mean ± SD).

* **Nominal concentration refers to the parent whole aged ZIF-8 suspension** from which fractions were generated. For comparability, **equal exposure volumes** of (i) particle-free filtrate and (ii) washed/resuspended particles/precipitates were used relative to the whole aged suspension at each nominal concentration.

**Fraction**	**Nominal concentration* (µg mL^{-1})**	**n (replicates)**	**Immobilisation at 48 h (%) mean ± SD**
Filtrate (particle-free)	0 (control)	4	0.0 ± 0.0
Filtrate (particle-free)	0.1	4	15.0 ± 10.0
Filtrate (particle-free)	0.5	4	35.0 ± 10.0
Filtrate (particle-free)	1.0	4	35.0 ± 10.0
Filtrate (particle-free)	5.0	4	40.0 ± 0.0
Washed ZIF-8 post-ageing (particles/precipitates)	0.1	4	30.0 ± 11.5
Washed ZIF-8 post-ageing (particles/precipitates)	0.5	4	40.0 ± 0.0
Washed ZIF-8 post-ageing (particles/precipitates)	1.0	4	55.0 ± 10.0
Washed ZIF-8 post-ageing (particles/precipitates)	5.0	4	65.0 ± 10.0
Whole aged ZIF-8 suspension	0.1	4	25.0 ± 10.0
Whole aged ZIF-8 suspension	0.5	4	35.0 ± 10.0
Whole aged ZIF-8 suspension	1.0	4	50.0 ± 11.5
Whole aged ZIF-8 suspension	5.0	4	65.0 ± 10.0

References

(1) Cravillon, J.; Münzer, S.; Lohmeier, S. J.; Feldhoff, A.; Huber, K.; Wiebcke, M. Rapid Room-Temperature Synthesis and Characterization of Nanocrystals of a Prototypical Zeolitic Imidazolate Framework. *Chemistry of Materials* 2009, *21* (8), 1410–1412. https://doi.org/10.1021/CM900166H.

(2) Pan, Y.; Liu, Y.; Zeng, G.; Zhao, L.; Lai, Z. Rapid Synthesis of Zeolitic Imidazolate Framework-8 (ZIF-8) Nanocrystals in an Aqueous System. *Chemical Communications* 2011, *47* (7), 2071–2073. https://doi.org/10.1039/C0CC05002D.

(3) Zhang, Y.; Jia, Y.; Li, M.; Hou, L. Influence of the 2-Methylimidazole/Zinc Nitrate Hexahydrate Molar Ratio on the Synthesis of Zeolitic Imidazolate Framework-8 Crystals at Room Temperature. *Sci. Rep.* 2018, *8* (1). https://doi.org/10.1038/S41598-018-28015-7.

(4) Chakraborty, S.; Dhumal, P.; Mikulska, I.; Pham, S.; (Bradford), L.-J. E.; Menon, D.; Misra, S. K.; Lynch, I. Biotic Transformation of Abiotically Stable Nanoscale UiO-66 Metal–Organic Framework by Daphnia Magna Results in Chronic Reproductive Toxicity. *ACS Nano* 2025. https://doi.org/10.1021/ACSNANO.5C16532.

(5) Howard, B. C.; Baker, I.; Kettridge, N.; Ullah, S.; Krause, S. Increasing the Scope of the Resazurin-Resorufin Smart Tracer System in Hydrologic and Biogeochemical Sciences: The Effects of Storage Duration and Temperature on Preservation. *Limnol. Oceanogr. Methods* 2022, *20* (11), 701–709. https://doi.org/10.1002/LOM3.10514.

(6) Zakowski, K.; Narozny, M.; Szocinski, M.; Darowicki, K. Influence of Water Salinity on Corrosion Risk - The Case of the Southern Baltic Sea Coast. *Environ. Monit. Assess.* 2014, *186* (8), 4871–4879. https://doi.org/10.1007/S10661-014-3744-3.

(7) Kayranli, B.; Ugurlu, A. Effects of Temperature and Biomass Concentration on the Performance of Anaerobic Sequencing Batch Reactor Treating Low Strength Wastewater. *Desalination* 2011, *278* (1–3), 77–83. https://doi.org/10.1016/J.DESAL.2011.05.011.
